# Modeling spatiotemporal abundance of mobile wildlife in highly variable environments using boosted GAMLSS hurdle models

**DOI:** 10.1002/ece3.4738

**Published:** 2019-02-14

**Authors:** Adam Smith, Benjamin Hofner, Juliet S. Lamb, Jason Osenkowski, Taber Allison, Giancarlo Sadoti, Scott R. McWilliams, Peter Paton

**Affiliations:** ^1^ Department of Natural Resources Science University of Rhode Island Kingston Rhode Island; ^2^ Department of Medical Informatics, Biometry and Epidemiology Friedrich‐Alexander‐University Erlangen‐Nuremberg Erlangen Germany; ^3^ Rhode Island Department of Environmental Management West Kingston Rhode Island; ^4^ American Wind Wildlife Institute Washington District of Columbia; ^5^ Department of Geography University of Nevada Reno Nevada; ^6^ Present address: United States Fish and Wildlife Service, National Wildlife Refuge System Inventory and Monitoring Branch Athens Georgia; ^7^ Present address: Section Biostatistics Paul‐Ehrlich‐Institut Langen Germany

**Keywords:** distribution, GAM, gradient descent boosting, hurdle models, Nantucket Sound, sea ducks, surveys

## Abstract

Modeling organism distributions from survey data involves numerous statistical challenges, including accounting for zero‐inflation, overdispersion, and selection and incorporation of environmental covariates. In environments with high spatial and temporal variability, addressing these challenges often requires numerous assumptions regarding organism distributions and their relationships to biophysical features. These assumptions may limit the resolution or accuracy of predictions resulting from survey‐based distribution models. We propose an iterative modeling approach that incorporates a negative binomial hurdle, followed by modeling of the relationship of organism distribution and abundance to environmental covariates using generalized additive models (GAM) and generalized additive models for location, scale, and shape (GAMLSS). Our approach accounts for key features of survey data by separating binary (presence‐absence) from count (abundance) data, separately modeling the mean and dispersion of count data, and incorporating selection of appropriate covariates and response functions from a suite of potential covariates while avoiding overfitting. We apply our modeling approach to surveys of sea duck abundance and distribution in Nantucket Sound (Massachusetts, USA), which has been proposed as a location for offshore wind energy development. Our model results highlight the importance of spatiotemporal variation in this system, as well as identifying key habitat features including distance to shore, sediment grain size, and seafloor topographic variation. Our work provides a powerful, flexible, and highly repeatable modeling framework with minimal assumptions that can be broadly applied to the modeling of survey data with high spatiotemporal variability. Applying GAMLSS models to the count portion of survey data allows us to incorporate potential overdispersion, which can dramatically affect model results in highly dynamic systems. Our approach is particularly relevant to systems in which little a priori knowledge is available regarding relationships between organism distributions and biophysical features, since it incorporates simultaneous selection of covariates and their functional relationships with organism responses.

## 1 INTRODUCTION

Understanding how the spatial distribution and abundance of an organism responds to biophysical features is fundamental to many aspects of ecology and conservation (Schröder & Seppelt, 2006). Since continuous sampling of the entire range or population of a species is usually impossible, distribution mapping typically involves a series of steps that include surveying a representative subset of the area or population of interest at various time periods, fitting models to represent the relationships of observed data to environmental covariates, using these models to predict utilization of un‐sampled areas or time periods based on biophysical habitat features, and finally validating predictions with on‐the‐ground observations (Borchers, Buckland, & Zucchini, 2002; Certain & Bretagnolle, 2008; Kinlan, Menza, & Huettmann, 2012; Nur et al., 2011). Although such model‐based approaches are widely used, their implementation requires addressing complex statistical challenges (Guisan & Thuiller, 2005), particularly for mobile organisms whose distributions and habitat requirements may vary in space and time (Runge, Martin, Possingham, Willis, & Fuller, 2014).

Spatiotemporal variability and uncertainty surrounding both the distribution of a species and key environmental covariates represent frequent challenges to the development of predictive models. Landscape‐ or population‐scale occupancy models may lack sufficient resolution to accurately address small‐scale spatial variation in habitat use; conversely, small‐scale models may be too precise to apply across landscapes (Johnson, 1980; Johnson, Nielsen, Merrill, McDonald, & Boyce, 2006; Johnson, Seip, & Boyce, 2004). Error can be introduced by spatial or temporal mismatches between occurrence estimates, environmental variables, and questions of interest (Austin & Van Niel, 2011; Guisan & Thuiller, 2005; Mainali et al., 2015). In addition to variability, uncertainty surrounding the biotic and abiotic factors determining the distribution of a species can also limit the development and implementation of model‐based distribution estimates (Thuiller, 2004). Because a priori knowledge of how occupancy and abundance relate to biophysical features is often lacking, survey data themselves can be used to select key environmental covariates (Guisan & Thuiller, 2005). This selection process requires choosing appropriate habitat variables from among a suite of intercorrelated covariates while avoiding overfitting (Hoeting, 2009; Merow et al., 2014). Most predictive models involve assumptions about the form of the response function between the occurrence or abundance of an organism and individual biophysical features. However, the information needed to inform these assumptions is typically unknown prior to analysis, which may lead to poor model performance (Austin, 2007; Mainali et al., 2015). Temporal variation in both the distribution and habitat preferences of a species can introduce further uncertainty, because organisms’ responses to changes in habitat conditions may not be instantaneous and may vary across the annual cycle (Selonen, Varjonen, & Korpimäki, 2015; Yamanaka, Akasaka, Yamaura, Kaneko, & Nakamura, 2015). Furthermore, both occupancy and abundance may respond not only to biophysical habitat features, but also to the distribution of other organisms such as conspecifics, competitors, predators, or prey (Blackburn, Cassey, & Gaston, 2006; Guisan & Thuiller, 2005).

Aside from their ecological complexity, survey data can present several statistical challenges to modeling and interpretation. Surveys can be modeled using occupancy (presence/absence) or abundance (numerical) approaches, which measure different aspects of habitat use and have different distributions and response functions. Given the additional statistical complexity involved in interpreting count data, abundance estimates are often overlooked when mapping the distribution of organisms (He & Gaston, 2000); however, occupancy estimates alone may provide incomplete or misleading information about habitat quality (Pulliam, 2000). Variation in abundance is often a key component of a species’ response to habitat quality and is crucial for accurately predicting species distributions (Howard, Stephens, Pearce‐Higgins, Gregory, & Willis, 2014; Johnston et al., 2015). The statistically challenging features of count data—particularly overdispersion, in which the variance of the data exceeds the mean—may in fact represent important biological responses to environmental features and conditions (McMahon, Purvis, Sheridan, Siriwardena, & Parnell, 2017; Richards, 2008). Modeling count data also often requires accounting for zero‐inflation (Martin et al., 2005), non‐linear responses to covariates (Cunningham & Lindenmayer, 2005), and spatial and temporal autocorrelation (Hoeting, 2009), which require a highly flexible modeling approach with few assumptions about either underlying distribution or response functions.

Generalized additive models (GAMs: Hastie & Tibshirani, 1990) and their extension, GAMs for location, scale, and shape (GAMLSS: Rigby & Stasinopoulos, 2005) offer several features that make them well‐suited for modelling complex, uncertain, or variable relationships between survey data and environmental covariates. GAMs do not assume linear effects on the response but flexibly adapt to the observed data, which makes them especially applicable to systems in which the form of the relationship between species occupancy, abundance, and underlying environmental conditions is often non‐linear and unknown (Guisan & Zimmermann, 2000). Moreover, unlike other generalized modeling approaches, GAMLSS allow both the mean and dispersion of the response to be modeled as a function of environmental covariates (Rigby & Stasinopoulos, 2005), which incorporates additional information about count data not reflected by mean values alone (McMahon et al., 2017). Despite these promising features, although GAM has recently gained popularity as a predictive distribution modeling approach (Miller, Burt, Rexstad, & Thomas, 2013), GAMLSS have yet to be widely adopted for modeling the spatial distribution of species based on biophysical features.

We propose a powerful, iterative modeling approach that combines GAM and GAMLSS in a gradient descent boosting framework (Hofner, Mayr, & Schmid, 2016; Hothorn, Bühlmann, Kneib, Schmid, & Hofner, 2010; Mayr, Fenske, Hofner, Kneib, & Schmid, 2012) to address the challenges of predicting occupancy and abundance from survey data in highly variable environments. Our approach independently evaluates environmental covariates for both occupancy and abundance, while allowing response functions to vary. We generate a single distribution estimate based on both occupancy and abundance that can be applied to environments with high levels of spatiotemporal variation and uncertainty. As a case study, we applied our proposed modeling framework to sea ducks (tribe Mergini) in Nantucket Bay, MA, USA. Understanding the winter habitat use and distribution of sea ducks in southern New England is crucial for the siting of proposed offshore wind farms (Bradbury et al., 2014; Langston, 2013); however, the implicitly high spatial and temporal variability of sea duck distributions, as well as poor understanding of habitat factors driving temporal and spatial variation in their distribution, has previously limited fine‐scale prediction of habitat use in the proposed construction area (Bowman, Silverman, Gilliland, & Leirness, 2015). We demonstrate an application of our modeling framework to informing conservation planning in the face of both high variability and ecological uncertainty by developing models from systematic aerial survey data of sea ducks in this system.

## 2 MATERIALS AND METHODS

**Figure 1:**
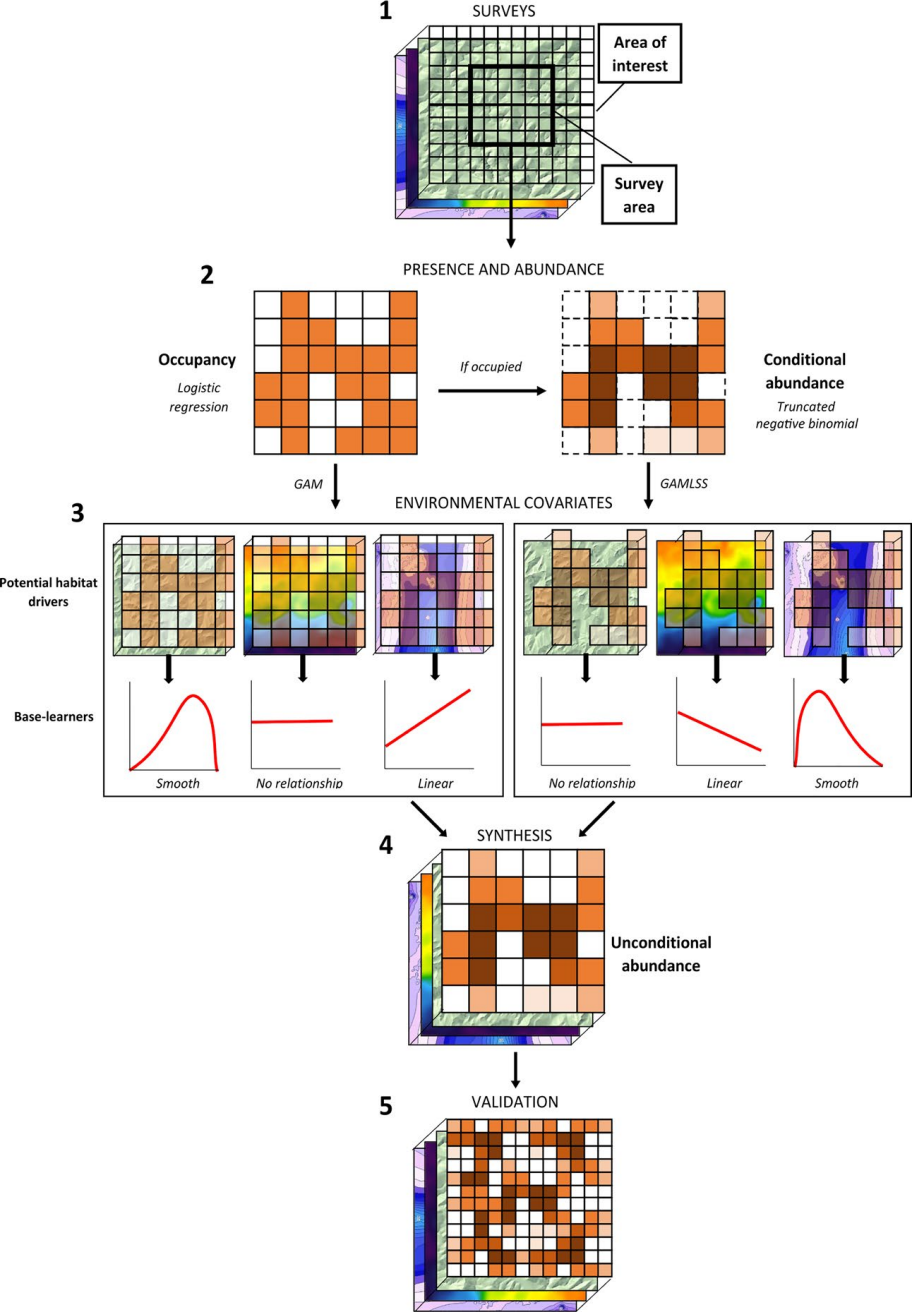
Schematic diagram of our modeling approach: (1) conducting initial transect surveys; (2) extrapolating occupancy probability and conditional abundance for each grid cell; (3) modeling relationships between occupancy, abundance, and habitat variables, (4) estimating unconditional abundance based on habitat characteristics, and (5) generating predictive estimates of occupancy and abundance over the full region. Detailed methodology for each step is provided in the corresponding numbered subsections in Section 2

Our predictive approach incorporates five distinct methodological steps: (a) survey data collection, (b) separation of presence from abundance, (c) integration of environmental covariates, (d) synthesis of presence and abundance models, and (e) validation, which correspond to numbered sections in the model schematic (Figure 1). We describe these five steps sequentially below, along with details of how we applied the modeling process to our case study of sea ducks in Nantucket Sound. All analyses were conducted in R (R Core Team, 2018) with the add‐on packages gamboostLSS (Hofner et al., 2017), mboost (Hothorn, Buehlmann, Kneib, Schmid, & Hofner, 2017), and stabs (Hofner & Hothorn, 2017).

### 2.1 Surveys

#### 2.1.1 Study system

**Figure 2:**
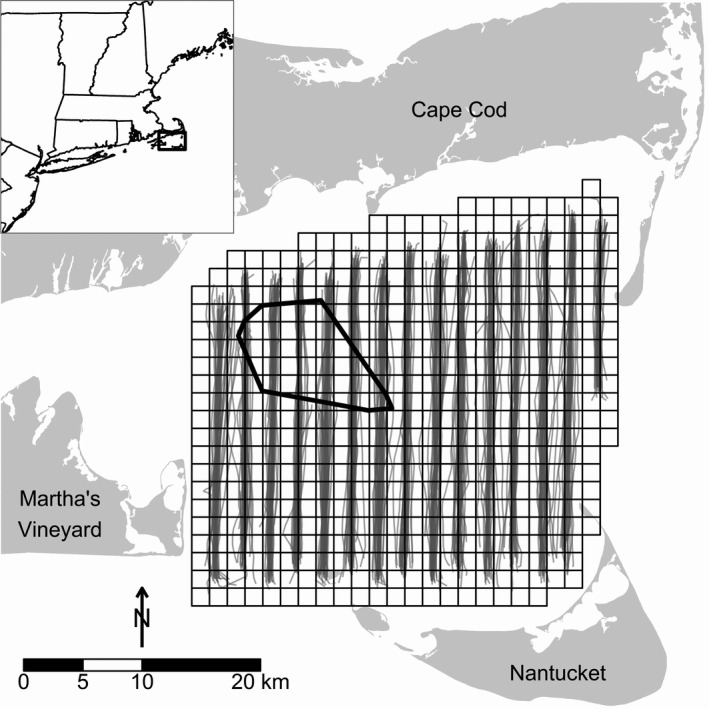
Aerial strip transect tracks (gray lines) conducted during winter (October–April, 2003–2005) sea duck surveys (*n* = 30) in Nantucket Sound, Massachusetts, USA. The grid indicates the extent of the 1,100 km^2^ study area and its division into 504 2.25 km^2^ segments. The polygon (thick black line) in northwest Nantucket Sound indicates a 62 km^2^ permitted wind energy development on Horseshoe Shoal

We conducted fieldwork throughout Nantucket Sound in Massachusetts, USA (Figure 2). Our study area encompassed ca. 1,500 km^2^ of Nantucket Sound, was relatively shallow (generally <20 m deep), and included some of the most important sea duck wintering habitat in the western Atlantic (Silverman, Saalfeld, Leirness, & Koneff, 2013; White, Veit, & Perry, 2009). The primary species of sea ducks found in Nantucket Sound were Common Eider (*Somateria mollissima*; hereafter eider), Black Scoter (*Melanitta. americana*), Surf Scoter (*M. perspicillata*), White‐winged Scoter (*M. deglandi*), and Long‐tailed Duck (*Clangula hyemalis*). Approximately 62 km^2^ of Horseshoe Shoal in northwestern Nantucket Sound is fully permitted for offshore wind energy development (OWED) (Figure 2; Santora, Hade, & Odell, 2004), although the proposed development was recently withdrawn.

#### 2.1.2 Survey design

During the boreal winters of 2003–2005, we conducted 30 (2003/2004:13, 2004/2005:10, 2005/2006:7) standardized aerial strip‐transect surveys (Flanders et al., 2015) (Figure 2). Surveys occurred primarily from November–March (*n* = 27), with occasional October (*n* = 1) or April (*n* = 2) surveys. During each survey, we flew along 15 parallel (ca. 2.5 km apart), roughly north‐south transects (Figure 2) using a high‐wing, twin‐engine Cessna Skymaster 337. We flew at an average altitude of 152 m and speed of 167 km/hr (90 kts), the slowest speed at which the aircraft could safely fly. This altitude allowed us to identify most birds at the sea surface and reduced disturbance (i.e., flushing birds to another part of the study area and potential double counting). We conducted surveys only on days with wind speeds ≤15 kts and good visibility (>15 km). Surveys had an average duration of ~2.5 hr and occurred between 0900 to 1600 hours to ensure that birds had completed any post‐dawn movements (Davis, 1997) but had yet to initiate pre‐sunset movements from feeding to roosting areas; this time window also reduced glare for observers due to low sun angles.

On each survey flight, two observers used their unaided eyes to continuously detect individuals or flocks, identified sea ducks to species with the aid of binoculars as needed, and communicated the number, species, and behavior (on the water or flying) of observed ducks to a recorder who entered georeferenced locations using dLOG (Ford, 1999). Observers monitored the sea surface on their side of the plane in a ca. 91 m‐wide transect between ca. 56 and 147 m from the plane. The narrow strip width ensured birds were detectable and identifiable with the naked eye and limited situations in which ducks were too abundant or spread over too wide an area to count accurately. We attempted to limit perception bias (i.e., to miss few individuals present to be counted; Marsh & Sinclair, 1989) by using low flight altitudes and narrow transect widths (Buckland et al., 2012; Certain & Bretagnolle, 2008); however, our survey methods did not address potential undercounting of individuals that were diving during flyovers, and therefore may not have been present to be detected. Transect dimensions resulted in the sampling of ~6% (median; 68.4 km^2^) of the study area during a survey.

Due to the difficulties associated with identifying to species the three species of scoters, we pooled all scoter observations (hereafter, scoters), while we modeled Common Eider and Long‐tailed Duck as separate species. While using pooled data from multiple scoter species reduces our ability to make inferences about species‐specific ecology and habitat use, scoters overlap broadly in shared wintering habitat across the study region and are generally subject to common conservation and management regimes. We subsequently consolidated counts for each species (eider and Long‐tailed Duck) or species group (scoters) into 2.25 km^2^ segments (Figure 2); this resolution (1.5 km × 1.5 km) corresponded approximately to the coarsest level of resolution of biophysical covariates (see below).

### 2.2 Presence and abundance

We related spatiotemporal variation in sea duck occupancy (i.e., probability of presence) and abundance to potentially relevant biophysical and spatiotemporal covariates. Because we observed a high incidence of zero counts (e.g., no eiders were detected in 75% of segment observations), and we assumed our survey design led to a low incidence of false zeros in study segments (Certain & Bretagnolle, 2008), we applied a negative binomial hurdle model (Manté, Kidé, Yao‐Lafourcade, & Mérigot, 2016). This approach allowed us to model presence/absence in all grid cells, and abundance only in cells where at least one individual was detected.

We first used a logistic regression model to represent the probability of occurrence of at least one individual (hereafter, the occupancy model) in a given segment (Figure 1:2). We then used a truncated negative binomial model to represent the abundance of sea ducks in that segment conditional on their presence (hereafter, the count model) (Figure 1:2). Occupancy and abundance values correspond to the probability of sea duck presence (occupancy) or sea duck abundance in a 1.5 km × ca. 180 m transect through a given segment during a single survey. We generated separate hurdle models for two sea duck species (Common Eider and Long‐tailed Duck) and one species group (scoters; Zipkin et al., 2010).

### 2.3 Environmental covariates

#### 2.3.1 Covariates

Distribution of large marine vertebrates is primarily a function of the distribution of preferred prey items. Since we did not have direct measurements of the availability of sea duck benthic prey (e.g., mollusks and crustaceans), we evaluated biophysical covariates expected to influence the distribution and abundance of these organisms, including water depth, sediment grain size, and primary productivity (Table 1). Additionally, we included interactions with time that allowed the effects of two ecological covariates (water depth and relative sea surface temperature) and all spatial covariates to vary over time within a given winter. We standardized (i.e., mean centered and scaled) all continuous covariates.

**Table 1:** Biophysical and survey covariates used to evaluate the distribution and abundance of Common Eider, Black, Surf, and White‐winged Scoter, and Long‐tailed Duck in Nantucket Sound during winters 2003–2005

Variable	Abbreviation	Units	Type[Link]	Description	Previous studies[Link]
Bathymetry	*depth*	m	S	Bottom depth relative to mean high water; lower values = deeper water (Eakins et al., 2009; National Oceanic & Atmospheric Administration, 2017)	Guillemette et al. (1993), Lewis et al., (2008), Winiarski et al. (2014), Flanders et al. (2015)
Sediment grain size	*meanphi*	phi	S	Sediment grain size (phi scale; Poti, Kinlan, & Menza, 2012: larger values =smaller grain sizes)	Goudie and Ankney (1988), Lovvorn et al. (2009), Loring et al. (2013)
Ratio of sea floor surface area to planimetric area	*SAR*	N/A	S	Topographic variability of the sea floor (calculated from bathymetry; Jenness, 2004)	Legendre et al. (1997), Knights, Crowe, and Burnell (2006)
Epibenthic tidal velocity (mean and standard deviation)	mean:* tidebmean* standard deviation:* tidesd*	m/s	S	Epibenthic tidal velocity during 2003–2005 based on monthly Finite‐Volume Community Ocean Model (FVCOM: Marine Ecosystem Dynamic Modeling Lab, [Link]; Chen, Liu, & Beardsley, 2003, Chen et al., 2011)	Legendre et al. (1997); Knights et al. (2006)
Water column stratification potential	*strat*	s^3^/m^2^	S	Potential for seasonal thermal stratification of the water column (Simpson & Hunter, 1974): ratio of depth (from bathymetry) to the third power of monthly surface tidal velocity (Simpson & Sharples, 2012)	Tremblay and Sinclair (1990), Raby, Lagadeuc, Dodson, and Mingelbier (1994), Witbaard and Bergman (2003)
Chlorophyll‐a	*chla*	mg/m^3^	S	Geometric mean of monthly composite chlorophyll‐a levels from July 2002 (first available) to March 2006; data from the Aqua MODIS satellite (ERDDAP, 2017)	Zipkin et al. (2010); Flanders et al. (2015)
Chromophoric dissolved organic material	*cdom*	N/A	S	Geometric mean of monthly composite chromophoric dissolved organic material levels (measured based on absorbance values) from July 2002 (first available) to March 2006 (ERDDAP, 2017)	
Sea bottom temperature	*SBT*	^o^C	ST	Epibenthic temperature averaged from May to October (bivalve settling period) in 2003–2005 from monthly FVCOM structured grids	Fay Neves and Pardue (1983); Newell (1989) Evans, Ford, Chase, and Sheppard (2011)
Sea surface temperature	monthly: *SST_m_*winter: *SST_w_* relative: *SST_rel_*	^o^C	ST	Sea surface temperature from monthly FVCOM structured grids. We included three *SST* values: monthly average, winter average (November through March), and relative (difference between the segment and the overall study area)	Zipkin et al. (2010), Flanders et al. (2015)
North Atlantic Oscillation (Dec–Mar)	*NAO_w_*	N/A	T	Winter (December through March) North Atlantic Oscillation index based on sea level pressure anomalies over the Atlantic sector (Hurrell, 1995; Hurrell & Deser, 2010; National Center for Atmospheric Research, 2017)	Zipkin et al. (2010)
Distance to land	*d2land*	km	S	Distance to the nearest location of zero depth (from bathymetry)	Guillemette et al. (1993), Lewis et al., (2008), Winiarski et al. (2014), Flanders et al. (2015)
Ferry route within 1 km	*ferry*	categorical	S	Indicator of whether a ferry route (Massachusetts Department of Transportation, Office of Transportation Planning) passed within 1 km a given segment. Ferries traversed this route ~16 times per day during the study period	Larsen and Laubek (2005), De La Cruz et al. (2014)
Day of year	*time*	day	T	Number of days before (negative) or after (positive) 31 December	
Winter	2004:* y2004* 2005: *y2005*	categorical	T	Study year indicator	
Easting	*xkm*	km	S	Distance west (negative) or east (positive) from the median longitude of all segments in the study area	
Northing	*ykm*	km	S	Distance south (negative) or north (positive) from the median latitude of all segments in the study area	
Survey effort	*obs_window*	km^2^	ST	Area surveyed in a given segment on a given date; calculated as the product of the length and width of the strip transect	
Spatiotemporal interactions	*xkm · ykm* xkm · time ykm · time *xkm · ykm · time*	N/A	ST	Interaction terms representing relationships between day of year and spatial distribution parameters	

Variable type: S (spatial; varying only among segments); T (temporal; varying consistently among segments over time); ST (spatiotemporal; varying both among segments and over time).

Studies suggesting a relationship of the variable to distribution of sea ducks and/or benthic prey populations.

#### 2.3.2 Modeling approach

To relate occupancy and abundance data to environmental covariates, we used additive models (Figure 1:3). We implemented a GAM for occupancy that flexibly accommodated varying effects of covariates on presence/absence data (Hastie & Tibshirani, 1990; Wood, 2006). For abundance data, we used a GAMLSS approach (Rigby & Stasinopoulos, 2005). Using GAMLSS allowed us to independently model dispersion of count data in relation to biophysical features, as well as account for potential non‐linear or heterogeneous responses of abundance to underlying environmental covariates (Stasinopoulos & Rigby, 2007).

We fitted GAM and GAMLSS using an iterative machine‐learning approach, component‐wise functional gradient descent boosting (Bühlmann & Hothorn, 2007; Hothorn et al., 2010; Mayr et al., 2012; Hofner, Boccuto, & Göker, 2015; Mayr & Hofner, 2018) in a cyclical framework (Thomas et al., 2018). The first step of this process was to compute the negative gradient of a pre‐selected loss function, which acts as a working residual by giving more weight to observations not properly predicted in previous iterations. We used the binomial log‐likelihood as the loss function for occupancy models and the truncated negative binomial log‐likelihood as the loss function for count models. For GAM, we computed the negative gradient of the mean only. For GAMLSS, we computed the negative gradient separately for mean and overdispersion in each iteration while holding the other parameter as a fixed constant (Mayr et al., 2012).

In the next step, we fitted various functional forms of each covariate relative to each response, called base‐learners (Hofner, Mayr, Robinzonov, & Schmid, 2014) to the negative gradients of the models. For each continuous covariate, we specified two possible base‐learners: a linear base‐learner and a base‐learner for the smooth deviation from the linear effect via penalized splines (i.e., P‐splines; Eilers & Marx, 1996; Schmid & Hothorn, 2008). This allowed the model to select the best alternative for each covariate between no effect, linear effect, and smooth effect. For categorical covariates, we coded the categories and used a separate linear base‐learner for each category, excepting a reference category (i.e., dummy‐coding). To address potential spatial autocorrelation, we included a smooth surface function of the spatial coordinates of segment centers (Kneib, Müller, & Hothorn, 2008), which accounted for underlying variance in sampling units similarly to a random effect term in a linear model. This surface comprised four base‐learners—linear base‐learners for the easting and northing, their linear interaction, and a penalized nonlinear tensor product (Kneib et al., 2008; Kneib, Hothorn, & Tutz, 2009; Maloney, Schmid, & Weller, 2012). We also allowed this surface to vary over time within a winter via an interaction. The decomposition of continuous covariates into linear and penalized nonlinear base‐learners reduced bias and overfitting by preventing the preferential selection of smooth base‐learners (Hofner, Hothorn, Kneib, & Schmid, 2011; Kneib et al., 2009). Thus, we restricted each base‐learner to a single degree of freedom and omitted the intercept term from each base‐learner (Hofner et al., 2011; Kneib et al., 2009) and added a linear base‐learner to the overall model to represent the model intercept. Once all potential base‐learners had been tested, the single best fitting base‐learner was added to the current model fit. As only the single best‐fitting base‐learner was selected in each iteration, the algorithm integrated intrinsic selection of the most relevant covariates and their functional form.

In order to maximize predictive accuracy while avoiding model overfitting, we employed an early stopping mechanism (Maloney et al., 2012; Mayr et al., 2012) during variable selection by stopping the algorithm prior to convergence to maximum likelihood estimates. In other words, after adding the best‐fitting base learner to the model, the negative gradient was then reevaluated at the current model fit and the procedure of testing, comparing, and adding base‐learners (Figure 1:3) was repeated until a pre‐specified number of iterations was reached (Bühlmann & Hothorn, 2007). We used 25‐fold subsampling to determine the optimal stopping iteration for each model. Specifically, we randomly drew (without replacement) 25 samples of size n/2 from the original data set. We used the selected sample to estimate the model and the balance of the data in each sample to determine the out‐of‐bag prediction accuracy (empirical risk) measured by the negative log‐likelihood of each model; the optimal stopping iteration (${\hat{m}}_{\text{stop}}$) is the iteration with the lowest average empirical risk. In boosted GAMLSS models we used multi‐dimensional subsampling to determine the stopping iteration for each of the GAMLSS parameters while allowing for potentially different model complexities in the parameters; a detailed explanation of this cross‐validation (subsampling) scheme is given in Hofner, Mayr, et al. (2016).

Since boosting methods typically produce "rich" models relying to some extent on many base‐learners (Hofner et al., 2015), we additionally used stability selection to compare the relative importance of covariates. Briefly, this process involved modeling subsamples of the data and measuring the frequency with which each covariate was included in the final models (Hofner et al., 2015). The methodology and results of this analysis are included in the Supporting information (Appendix S1).

### 2.4 Synthesis

Both GAM and GAMLSS models took the following general form:

$$g\left(\cdot \right)=\text{int}+{\text{covariate}}_{1}+f\left({\text{covariate}}_{1}\right)\cdots +{\text{covariate}}_{n}+f\left({\text{covariate}}_{n}\right)$$

For occupancy models (GAM), we modeled the occupancy probability of a given duck species in a segment *g*(*π*_sea ducks_) as a function of all environmental covariates (Table 1), with *g* representing the logit link. Count models (GAMLSS) took two forms: the (conditional) mean count of sea ducks, *g*(*μ*_sea ducks_), and the (conditional) overdispersion in sea duck counts, *g*(*σ*_sea ducks_); *g* is the log link in both cases. The same environmental covariates were included in both models (Table 1). *f*(·) indicates the penalized nonlinear deviations from the corresponding linear base‐learners (e.g., *f*(*time*)) and were included for all non‐categorical variables.

We included interaction terms between easting (*xkm*), northing (*ykm*), and day of year (*time*) to incorporate spatiotemporal effects. The explicit intercept (*int*) was a necessary byproduct of our decomposition of base‐learners (Hofner et al., 2011; Kneib et al., 2009). We included *obs_window*, our measure of survey effort (Table 1), as a covariate rather than an offset because small values in some segments impaired estimability.

Subsequent to their independent fitting, we consolidated occupancy and conditional count models into a single model (see Equation 6 in Zeileis, Kleiber, & Jackman, 2008) to generate unconditional, spatially‐explicit estimates of sea duck abundance.

### 2.5 Validation

Since additional test data were not available for our study area, we used a pseudo *R* ^2^ measure of the explained variation (Maloney et al., 2012; Nagelkerke, 1991) to evaluate the approximate explanatory power of our final models. We obtained the pseudo *R* ^2^ value by comparing the log likelihood values for our model‐generated estimates of unconditional abundance for each species to log likelihood values obtained from null (intercept‐only) models, giving us an estimate of the increase in explanatory power provided by our models.

## 3 RESULTS

**Figure 3:**
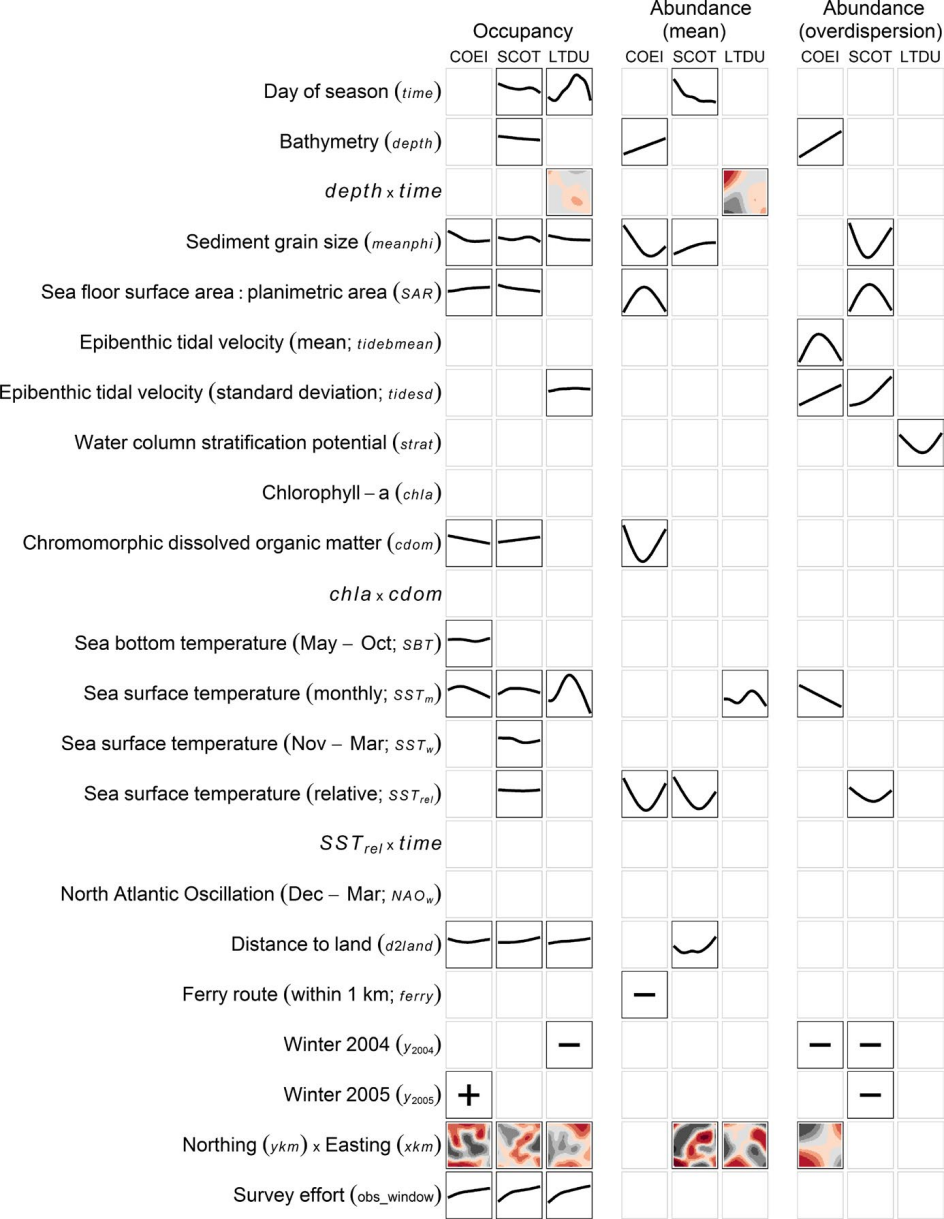
Marginal functional plots for stably selected covariates in the occupancy (probability of presence) and conditional abundance (mean and overdispersion) models for Common Eider (COEI), scoters (SCOT), and Long‐tailed Duck (LTDU) in Nantucket Sound during three winters (2003–2004 to 2005–2006). Each plot illustrates the partial contribution of a covariate to the additive predictor (Y‐axis), holding all other covariates at their mean. Within a model, plots share a *Y*‐axis scale, enabling direct comparisons of effect sizes among covariates and species. Bivariate plots reflect the first (*Y*‐axis) and second (*X*‐axis) variables listed in the interaction; colors indicate the direction and magnitude of the partial contribution (blacks = negative, reds = positive; darker colors = larger effect). Northing by easting effects are given only at 31 December. For factor variables, only the general association (positive or negative) with the additive predictor is given. Covariate abbreviations correspond to Table 1

For each species or species group, we fitted independent models for occupancy and conditional count data (mean and overdispersion). Bootstrapped empirical risk suggested that occupancy models for all species converged to the maximum likelihood estimates (i.e., occupancy models failed to stop early; see Supporting information Appendix S2). Conversely, bootstrapped empirical risks prescribed early stopping for both the conditional mean and overdispersion parameter in all count models (see Supporting information Appendix S2). Final occupancy models and models for the conditional mean of count data included only a subset (12% to 38%) of the 48 base‐learners initially specified for selection. Occupancy models generally contained more covariates and their interactions (8–10 of 23) than did count models (3–6 of 23), particularly among stably selected covariates and their interactions (Figure 3, see also Supporting information Appendix S3).

### 3.1 Sea duck occupancy

Three covariates—grain size, sea surface temperature, and distance to land—were associated with probability of occurrence for all three sea duck species or species groups (Figure 3). Standardized effects of each variable on the response can be compared among species and covariates within a model based on their range on the *Y*‐axis. For example, monthly sea surface temperature (*SST_m_*) spanned a larger range of the *Y*‐axis, and thus associated more strongly with eider occupancy, than did distance to land (d2land) (Figure 3). In contrast, monthly sea surface temperature (*SST_m_*) associated much more strongly with occupancy of Long‐tailed Duck than with eider or scoters (Figure 3). Detailed comparisons of univariate, bivariate, and categorical effects for each species are included in the Supporting information (Appendix S3).

Spatiotemporal effects (i.e., occupancy associated with the *xkm‐ykm* location of segments and the change over time within winter [*time*]) were the dominant explanatory feature in occupancy models, although these patterns varied considerably among species (Figure 3; see Day of season, Northing x Easting). Occupancy increased, but at a decreasing rate, with survey effort (*obs_window*) in a given segment (Figure 3). Occupancy estimates increased at intermediate monthly sea surface temperature (*SST_m_*), greater distances from land (*d2land*), and in areas with coarser sediments (i.e., smaller *meanphi*). Eider occupancy was associated negatively with chromomorphic dissolved organic material (*cdom*) and positively with sea floor surface area relative to planimetric area (*SAR;* our measure of the topographic variability of the sea floor; Figure 3), whereas scoter occupancy likewise related to *SAR* and *cdom*, but in the opposite direction in both cases (Figure 3). Scoters occupancy was modestly greater in deeper waters (*depth*), whereas Long‐tailed Duck occupancy was greatest in shallow waters early in the winter but in deeper waters later in the winter (Figure 3; depth × time covariate). Other effects were relatively minor and inconsistent among species.

**Figure 4:**
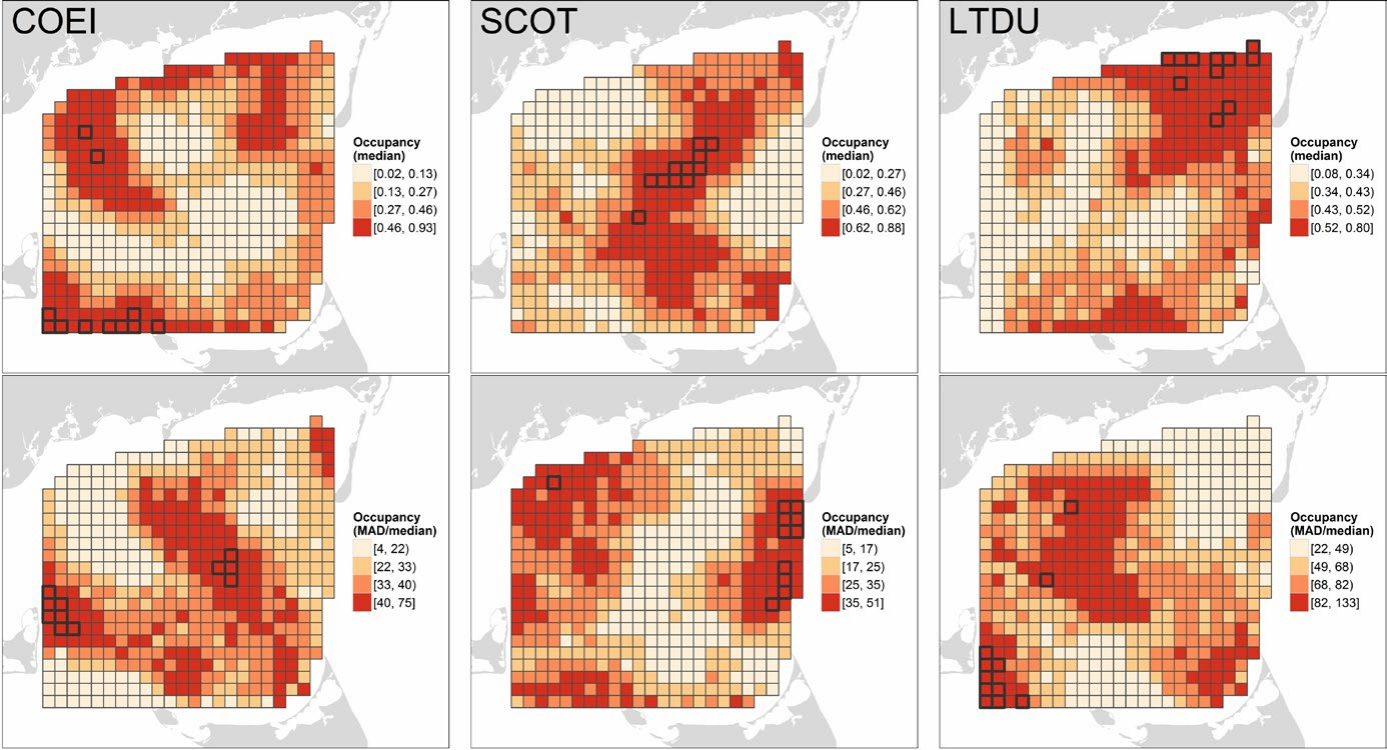
Occupancy probability for Common Eider (COEI), scoters (SCOT), and Long‐tailed Duck (LTDU) in Nantucket Sound during three winters, 2003–2005. Occupancy probabilities (top row) represent the median expected probability of sea duck presence in a 1.5 km × ca. 180 m transect through a given segment predicted on 10 evenly‐spaced dates from 15 November through 1 April in each winter. Spatiotemporal variation in occupancy (%; bottom row) is indicated by the median absolute deviation, MAD, of occupancy probability relative to the median. Predicted values are categorized based on their quartiles; segments with the highest occupancy or variability (values ≥98th percentile) are outlined in black

The predominance of spatial effects (*ykm‐xkm*) resulted in distinct spatial patterns of occupancy among species (Figure 4, top row) despite the relative similarity of occupancy associations with biophysical covariates (Figure 3). Occupancy was typically highest for eider in northwest and southwest Nantucket Sound, in interior Nantucket Sound for scoters, and in northeast and south Nantucket Sound for Long‐tailed Duck (Figure 4, top row). All species tended to avoid the western edge of the Sound northeast of Martha's Vineyard. Generally, the areas of highest occupancy exhibited the lowest relative variability (Figure 4, bottom row), defined as the median absolute deviation (MAD) of occupancy relative to median occupancy within a segment (a measure analogous to the coefficient of variation, but in this case providing an estimate of temporal variability).

### 3.2 Sea duck conditional abundance and overdispersion

Spatial effects (*ykm‐xkm*) were the dominant explanatory feature of conditional abundance estimates for scoters and Long‐tailed Duck, but they were not selected in the eider model (Figure 3). In contrast with the corresponding occupancy model, scoter conditional abundance decreased with increasing sediment grain size (*meanphi*). Additionally, the relationships between eider conditional abundance and dissolved organic material (*cdom*) and sea floor topography (*SAR*; Figure 3) were more complex than with eider occupancy. The conditional abundance of eider and scoter was also associated with relatively warm or cool sea surface temperatures (*SST*_rel_; Figure 3). Biophysical covariates associated with Long‐tailed Duck conditional abundance exhibited general agreement with their counterpart in the occupancy models.

**Figure 5:**
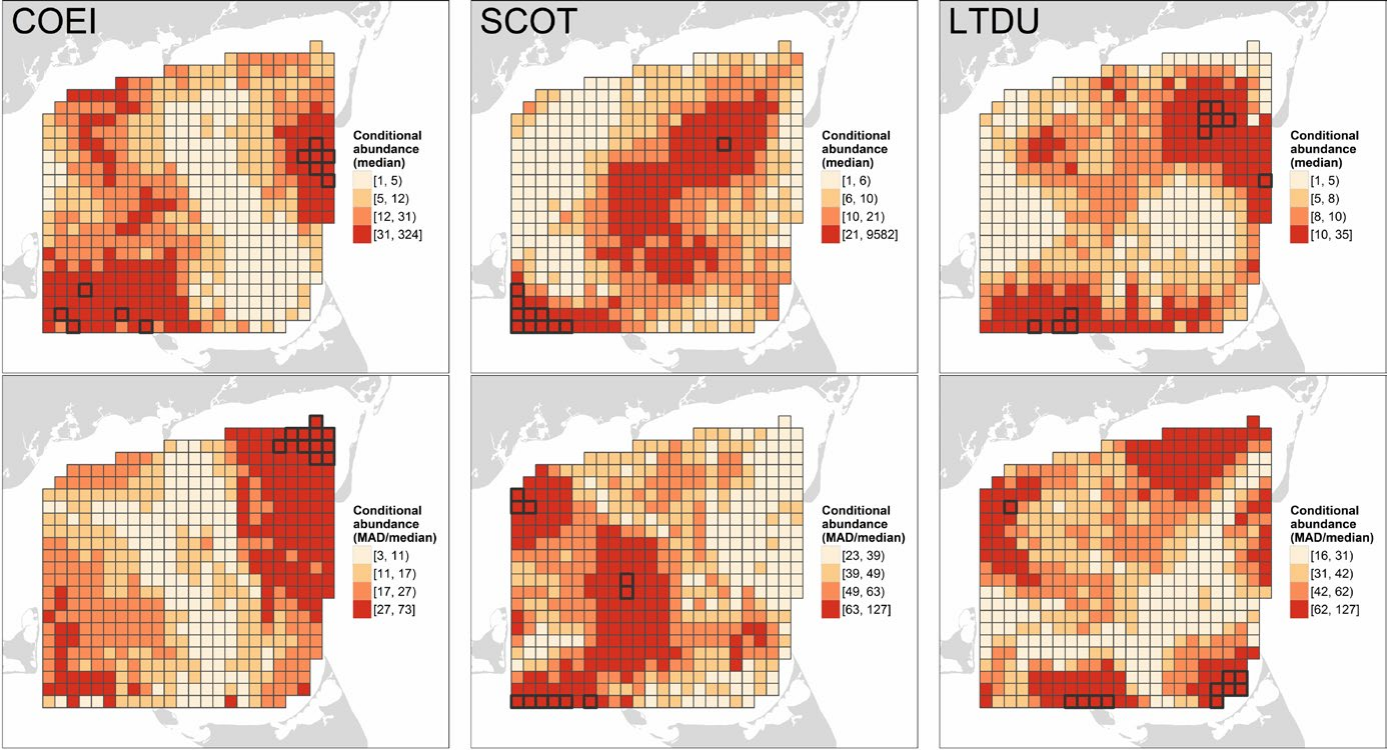
Conditional abundance of Common Eider (COEI), scoters (SCOT), and Long‐tailed Duck (LTDU) in Nantucket Sound during three winters, 2003–2005. Conditional abundances (top row) represent the median expected number of sea ducks, assuming their presence, in a 1.5 km × ca. 180 m transect in each segment predicted on 10 evenly‐spaced dates from 15 November through 1 April in each winter. Spatiotemporal variation in conditional abundance (%; bottom row) is indicated by the median absolute deviation, MAD, relative to the median. Predicted values are categorized based on their quartiles; segments with the highest conditional abundance or variability (values ≥98th percentile) are outlined in black

Spatially‐explicit patterns of occupancy (cf. Figure 4, top row) did not necessarily reflect patterns of median conditional abundance (Figure 5, top row). Some areas of Nantucket Sound exhibited mutually high conditional abundance and occupancy for a given species (e.g., eider in the southwest, scoter in the interior, and Long‐tailed Duck in parts of the northeast). However, conditional abundance was low despite relatively high occupancy in some instances (e.g., eider in the northeast and Horseshoe Shoal, scoters in the northeast and southeast, and Long‐tailed Duck along the northern margin). Conversely, other areas of Nantucket Sound exhibited lower occupancy but sea ducks, when present, were more abundant (e.g., eider along the eastern margin, and scoters and Long‐tailed Duck in the southwest). As in occupancy models, sea ducks were relatively absent from the middle‐western margin of Nantucket Sound (i.e., northeast of Martha's Vineyard; see Figure 3). In contrast to occupancy, which was less variable in areas of high abundance, areas of high conditional sea duck abundance typically also exhibited high relative variability over time (Figure 5, bottom row).

Overdispersion in conditional sea duck abundance also varied with biophysical covariates, although relationships were less consistent among species (Figure 3; see also Supporting information Appendix S3). Variability (i.e., overdispersion) in sea duck counts was heterogeneous in space and time in Nantucket Sound (Supporting information Appendix S4), particularly for eider and scoters (as indicated by the magnitude of the overdispersion parameter values).

### 3.3 Expected sea duck abundance

**Figure 6:**
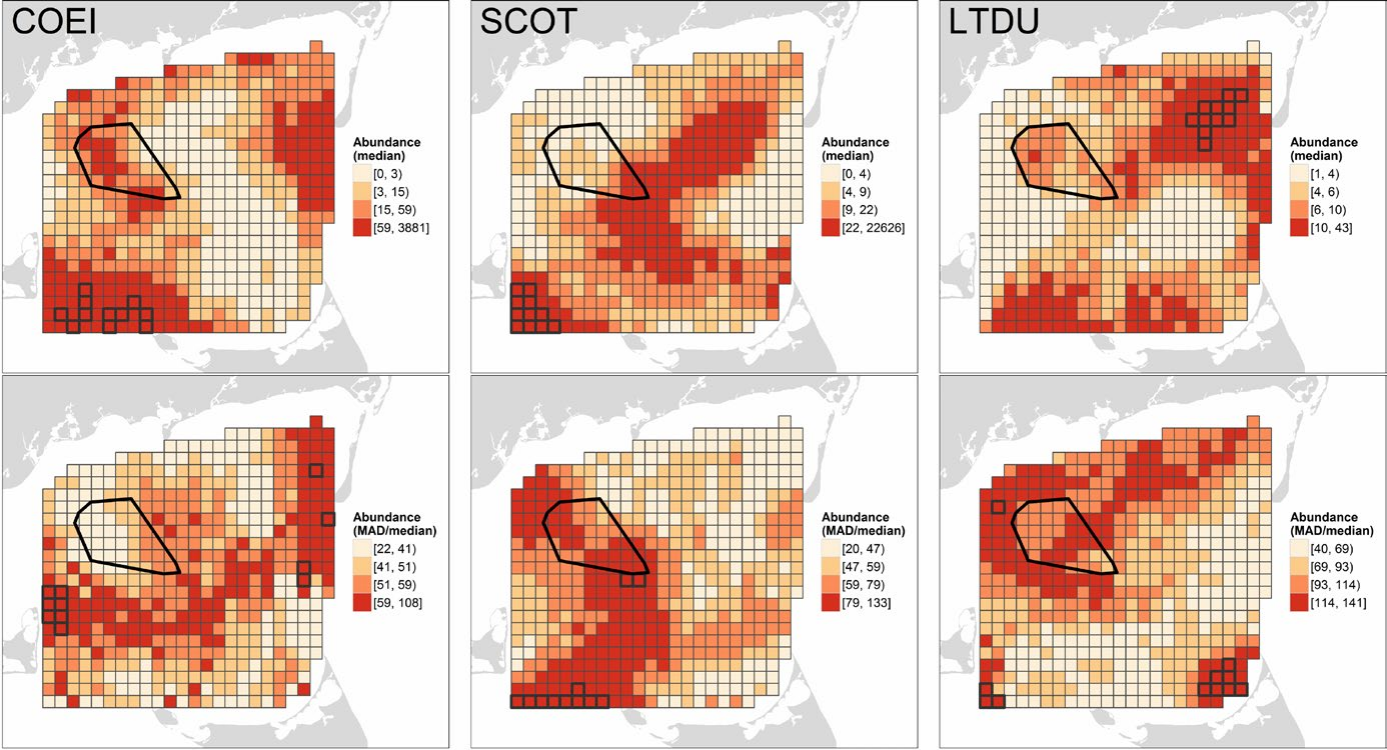
Unconditional abundance of Common Eider (COEI), scoters (SCOT), and Long‐tailed Duck (LTDU) in Nantucket Sound during three winters, 2003–2005. Median abundances (top row) represent the expected number of sea ducks along a 1.5 km × ca. 180 m transect within each segment predicted on 10 evenly‐spaced dates from 15 November through 1 April in each winter. Spatiotemporal variation in abundance (%; bottom row) is estimated from the median absolute deviation, MAD, relative to the median. Predicted values are categorized based on their quartiles; segments with the highest abundance or variability (values ≥98th percentile) are outlined in black

Consolidated occupancy and conditional count models provided estimates of unconditional sea duck abundance in the study area over the survey period. Final models of expected sea duck abundance explained moderate amounts of variation in observed counts of eider, scoters, and Long‐tailed Duck (pseudo *R* ^2^ = 0.31, 0.48, and 0.32, respectively). Conditional abundance (Figure 5) strongly influenced the spatially‐explicit patterns of expected abundance. Sea duck species exhibited relatively distinct patterns of abundance in Nantucket Sound. Eider were consistently most abundant in southwestern Nantucket Sound. They also were relatively abundant in the northeastern part of the study area but less consistently based on the relatively high MAD/median abundance over time (Figure 6). Scoters were also most abundant, with occasional extremely large flocks, in southwestern Nantucket Sound. This was also the area of highest relative variation in scoter abundance; relatively high abundances of scoters also occurred in interior Nantucket Sound (Figure 6). Long‐tailed Ducks were consistently most abundant in northeastern Nantucket Sound, as well as along its southern margin (Figure 6). No species' highest abundances occurred in the permitted Nantucket Shoal area, although expected eider and scoters abundances were consistently elevated in some parts of the Shoal (west and southeast, respectively; Figure 6).

**Figure 7:**
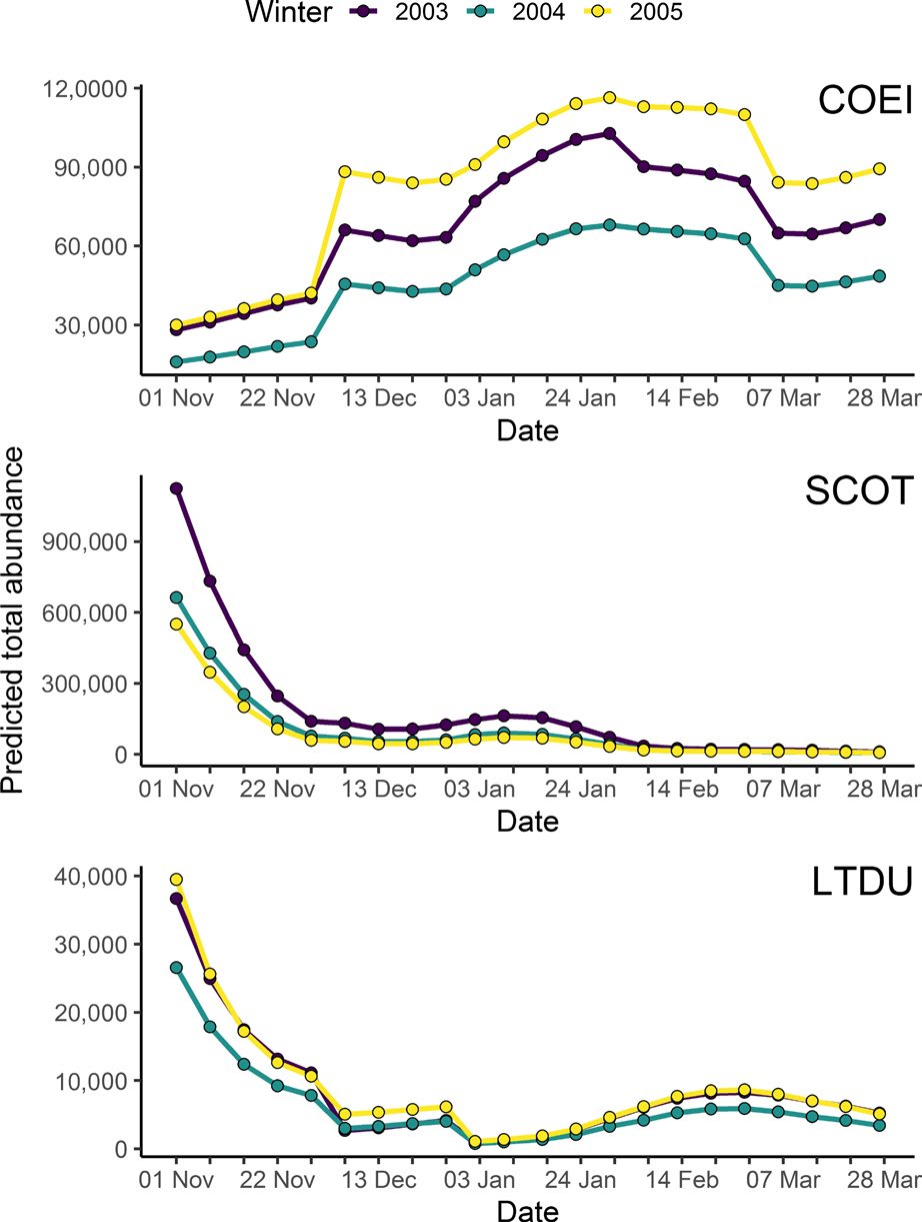
Estimated weekly total abundance of Common Eider (COEI), scoter (SCOT), and Long‐tailed Duck (LTDU) in the entire study area over three winters, 2003–2005

**Figure 8:**
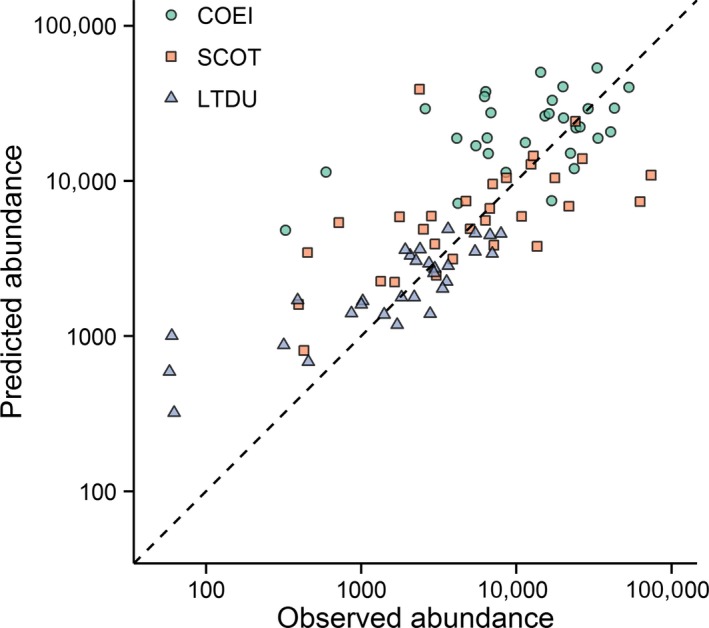
Relationship between observed and predicted total abundance of Common Eider (COEI), scoters (SCOT), and Long‐tailed Duck (LTDU) during 30 aerial surveys of Nantucket Sound over three winters, 2003–2005. The dashed line indicates a 1:1 relationship between predicted and observed abundances in surveyed segments; points below and above this line indicate underestimates and overestimates of predicted abundances, respectively

Summing the spatially‐explicit estimates of unconditional sea duck abundance (i.e., Figure 6) provides an estimate of total abundance in a 1.5 km × 180 m transect through all segments in the study area. We calculated overall abundance by species throughout the study area by extrapolating these estimates across the full study area (Figure 7). Although absolute estimates differed between study years, patterns of abundance were similar across years, with scoter and long‐tailed duck abundances highest early in the season, and eider abundance peaking in mid‐winter. We also compared the total count (summed across all segments) of each sea duck species observed in aerial strip transects with the corresponding estimated total abundance in surveyed segments for each of the 30 aerial surveys (Figure 8). Our models tended to overestimate sea duck abundance when the actual numbers of sea ducks were relatively low, although overestimation was typically less than an order of magnitude. This pattern may have resulted from patterns of seasonal variation: since not all individuals arrive in or depart from the study area at the same time, individuals present in the study area early and late in the winter season likely occurred in lower densities than expected relative to habitat features. Additionally, scoter abundance was occasionally extreme relative to typical counts and somewhat prone to underestimation during these extreme counts, likely because extremely high counts were too infrequent to allow accurate assessment of the factors influencing their occurrence. Nonetheless, the general adherence of observed and predicted abundance to a line of unit slope indicated that it may be reasonable to estimate sea duck abundance for the entire study area based on observed sea duck densities in transects (Figure 8).

### 3.4 Temporal dynamics in wintering sea ducks

The MAD/median estimates (Figures 4, 5, 6, bottom rows; Supporting information Appendix S4) show that our spatially‐explicit estimates of occupancy, abundance, and overdispersion invariably change over time, either explicitly via the selection of a within‐ or among‐winter temporal effect (*time* and *y2004*/*y2005*, respectively) or implicitly via the selection of biophysical covariates that change within or among winters. The temporal dynamics of the wintering sea duck system in Nantucket Sound was one of its most striking attributes, and we illustrate these dynamics with an animation for scoter occupancy and abundance in the Supporting Information (Appendix S5).

## 4 DISCUSSION

We demonstrated a flexible model‐based approach to evaluate the environmental associations of sea duck distribution and abundance based on multiyear replicated surveys. The boosted GAMLSS framework offered several useful features including (a) the ability to model all parameters of the conditional distribution (e.g., conditional mean and overdispersion) as a function of covariates, (b) integrated variable reduction and selection among many covariates, and (c) integrated model selection via model decomposition of continuous covariates and thus the simultaneous consideration of competing functional forms (e.g., linear vs. non‐linear). Additionally, this framework allowed us to incorporate smooth effects to efficiently account for spatiotemporal trends in the data that were poorly explained by other covariates and to identify those covariates and their functional forms most consistently associated with animal distribution and abundance (via stability selection). Recent advances in the application of gradient boosting (non‐cyclical application: see Thomas et al., 2018) could allow for even greater power in selecting appropriate variables and responses from among available covariates.

Although negative binomial hurdles and boosted GAM/GAMLSS have previously been used to predict organism distributions, to the best of our knowledge, they have not yet been combined into a single modeling framework. In order to effectively model organism distributions in relation to biophysical features from survey data, both modeling components address important characteristics of the data. Applying a negative binomial hurdle allows simultaneous modeling of both presence/absence and abundance data, and subsequently applying GAM and GAMLSS accounts for unique aspects of each data type, particularly overdispersion of the abundance (count) portion of the data. As a final step, the data are recombined to produce predictions that flexibly incorporate a wide range of potential responses to environmental covariates, which is particularly crucial in systems with little a priori knowledge regarding the relationships between organism distributions and environmental covariates. These predictions can be used to generate a variety of information on species abundance and distribution, including overall abundance estimates (this study), as well as estimates of prediction error, which can be generated via bootstrapping by repeated runs of the model (Hofner, Kneib, & Hothorn, 2016).

The useful features of this modeling framework apply especially to mobile species with non‐uniform distributions that vary among and within years, such as the species of sea ducks that we studied. Our estimates of the spatiotemporal abundance of sea ducks in Nantucket Sound were controlled largely by estimates of the conditional abundance and less by spatiotemporal patterns in the occupancy of sea ducks. This suggests that occupancy models alone may be inadequate for assessing risk from anthropogenic disturbances and for describing the fine‐scale distribution of marine species. Previously, the statistically challenging features of count data have restricted their use in distribution models, meaning that most predictions have addressed only occupancy (Flanders et al., 2015; Winiarski, Miller, Paton, & McWilliams, 2014), and may thus have ignored important facets of sea duck distribution and risk exposure, particularly variation in abundance. For species such as sea ducks, which gather in dense social aggregations at preferred habitat locations, flock size is a key component of distributional patterns as it both reflects and enhances habitat suitability (Guillemette, Himmelman, & Barette, 1993) and may affect the distribution and intensity of risk factors on individuals (e.g., Schwemmer, Mendel, Sonntag, Dierschke, & Garthe, 2011). Our modeling approach thus allows us to examine key features of sea duck distribution patterns that have been overlooked in previous studies.

### 4.1 Environmental covariates that best explain sea duck distribution and abundance

The biophysical associations with sea duck occupancy derived from our models were relatively consistent among species, whereas their associations with sea duck conditional abundance were more species‐specific. Distance to land, which was associated with both occupancy and abundance, tends to be positively associated with bathymetry and often has a strong influence on sea duck occupancy estimates (Flanders et al., 2015; Guillemette et al., 1993; Lewis, Esler, & Boyd, 2008; Winiarski et al., 2014). Sediment grain size can also affect prey availability for foraging sea ducks (Goudie & Ankney, 1988; Loring, Paton, McWilliams, McKinney, & Oviatt, 2013; Lovvorn, Grebmeier, Cooper, Bump, & Richman, 2009) and was associated with occupancy and conditional abundance in this study. In addition, topographic variability of the sea floor also influenced occupancy and conditional abundance, although its relationship to prey availability is less understood. We did not find evidence for temporally varying associations of sea duck assemblages with dynamic oceanographic conditions such as sea surface temperature, chlorophyll *a*, or the North Atlantic Oscillation (NAO). These results contrast with several previous studies of sea duck occupancy (Flanders et al., 2015; Zipkin et al., 2010) that have documented effects of dynamic oceanographic conditions on sea duck distributions. Certain covariates may associate with marine bird abundance or behavior at specific scales and not at others (Logerwell & Hargreaves, 1996; Mannocci et al., 2017); thus, the smaller spatial scale of our analysis compared to previous studies may explain the apparent discrepancy between studies in the effect of chlorophyll *a* and NAO.

The unexplained variation in our models and the predominance of marginal spatiotemporal effects suggest that we likely missed important variable(s) relevant to the distribution of sea ducks in Nantucket Sound. This lack of explanatory power suggests a need for better biophysical proxies for the distribution of prey eaten by sea ducks or concurrent prey distribution information (Cervencl & Fernandez, 2012; Cervencl et al., 2014; Kaiser et al., 2006; Vaitkus & Bubinas, 2001; Žydelis, Esler, Kirk, & Boyd, 2009), although such data are challenging to obtain at appropriate resolutions and may not guarantee improved predictive accuracy (Benoit‐Bird et al., 2013; Grémillet et al., 2008; Torres, Read, & Halpin, 2008). Additionally, our survey methods may have resulted in either over‐ or under‐counting, depending on both movement and diving behavior of birds. Given the dominant effects of large flock sizes on our predictions, we expect that the magnitude of detection bias would not have been large enough to substantially affect our results. However, future surveys could benefit from recent developments in survey design (Conn & Alisauskas, 2018) that have been proposed to address the particular biases associated with aerial counts of waterfowl.

### 4.2 The importance of spatial scale

The process whereby migratory animals such as sea ducks select a given area to inhabit during winter involves decisions at multiple spatial scales and the environmental attributes that determine this habitat selection often vary with spatial scale (Johnson, 1980; Johnson et al., 2006, 2004). The majority of North American sea ducks migrate from high‐latitude arctic and sub‐arctic breeding areas to mid‐latitude temperate wintering areas where they reside for most of the year (Bowman et al., 2015; Flanders et al., 2015; Silverman et al., 2013). At these large spatial scales, the distribution and abundance of sea ducks during winter may be affected by large‐scale ocean characteristics (Flint, 2013), climatic conditions (Zipkin et al., 2010), and static or persistent habitat features (e.g., bathymetry, substrate, current and frontal systems; Hyrenbach, Forney, & Dayton, 2000; Nur et al., 2011; Flanders et al., 2015). At regional and local scales, however, most species of sea ducks congregate in large flocks (e.g., up to tens of thousands of birds) at sites where prey are abundant and accessible (Flint, 2013; Loring et al., 2013) although the abundance and distribution of these prey, and thus predators, can be extremely ephemeral and dynamic (Cisneros, Smit, Laudien, & Schoeman, 2011; Hyrenbach et al., 2000).

Given that sea duck distribution and abundance is spatially and temporally dynamic, yet expected to be driven by biophysical covariates (Flanders et al., 2015; Oppel, Powell, & Dickson, 2009; Zipkin et al., 2010) that may differ in importance depending on spatial scale (Johnson, 1980; Johnson et al., 2006, 2004), marine spatial planners must carefully consider the most appropriate information to use when deciding, for example, where to place marine protected areas or offshore wind energy developments to meet conservation and management goals. A larger‐scale occupancy model developed by Flanders et al. (2015) suggested that eiders were relatively uniformly distributed across Nantucket Sound, whereas our higher resolution abundance models found that eiders were concentrated in southwestern and central, eastern areas within Nantucket Sound. While large‐scale models (Flanders et al., 2015; Silverman et al., 2013) are useful to identify general geographic areas of importance to sea ducks, our models provide more detailed estimates of sea duck distribution and abundance within a specific area of interest. In terms of marine spatial planning, large‐scale models can be used to inform the siting of lease areas or protected areas, followed by detailed modeling approach such as ours to select sites within these larger blocks that can be zoned for specific levels of development or protection.

## CONFLICT OF INTEREST

None declared.

## AUTHOR CONTRIBUTIONS

TA and GS designed and collected the survey data. AS, BH, SM, and PP conceived the analytical methodology. AS and BH analyzed the data. AS, JL, BH, SM, and PP led the writing and editing of the manuscript. AS and JL designed tables and figures. All authors contributed critically to the drafts and gave final approval for publication.

## Data Availability

Raw survey data, fitted models, and code: Dryad Data Repository ( https://doi.org/10.5061/dryad.1vm20t6).
