# Supplementary material for: Modeling spatiotemporal abundance of mobile wildlife in highly variable environments using boosted GAMLSS hurdle models
Source: Ecol Evol. 2019 Feb 14;9(5):2346–64. doi: 10.1002/ece3.4738 (PMC6405508; doi:10.1002/ece3.4738)
Supplement: Supplementary file 1 [file ECE3-9-2346-s001.pdf]

S.1 Stability selection

Methods

We applied stability selection (Meinshausen & Bühlmann 2010; Shah & Samworth 2013; see also Hofner *et al.* 2015 for details in the context of boosting) to identify base-learners, and thus covariates, that were commonly selected in the majority of randomly drawn subsamples of size $\lfloor n/2 \rfloor$ of the data. As proposed by Shah & Samworth (2013), we used $B =$ 50 complementary pairs subsamples (i.e., we randomly split the data into two halves and used both to independently fit the model). This resulted in 100 total subsamples. We set the number of selected base-learners per boosting model (q) to 35 and established upper bounds of three and six for the occupancy and count model per-family error rates (PFER), respectively. These error bounds corresponded to an upper bound of $\alpha = 0.062$ for the per-comparison error rate in both models. The different thresholds reflect the different number of base-learners in the two models; occupancy models contained 48 base-learners while the count models contained twice as many base-learners (i.e., 48 each for the mean and overdispersion parameter). The choice of q is somewhat arbitrary; it is chosen to be large enough to incorporate all important variables in the model (Hofner *et al.* 2015). We used the unimodality assumption for the computation of the error bounds in the occupancy and count models (Shah & Samworth 2013; Hofner *et al.* 2015).

Results

Occupancy models.—Given our specifications ($q = 35$; PFER upper-bound = 3, unimodality assumption), only base-learners selected in at least 99 of the 100 subsamples (i.e., $\pi_{\text{thr}} = 0.99$) were identified as stable (Figure S1.1).

Count models.—Given our specifications ($q = 35$; PFER upper-bound = 6, unimodality assumption), only base-learners selected in at least 90 of the 100 subsamples (i.e., $\pi_{\text{thr}} = 0.9$) were identified as stable (Figure S1.2); this threshold applies to the simultaneous selection of base-learners for the conditional mean (μ) and conditional overdispersion (σ).

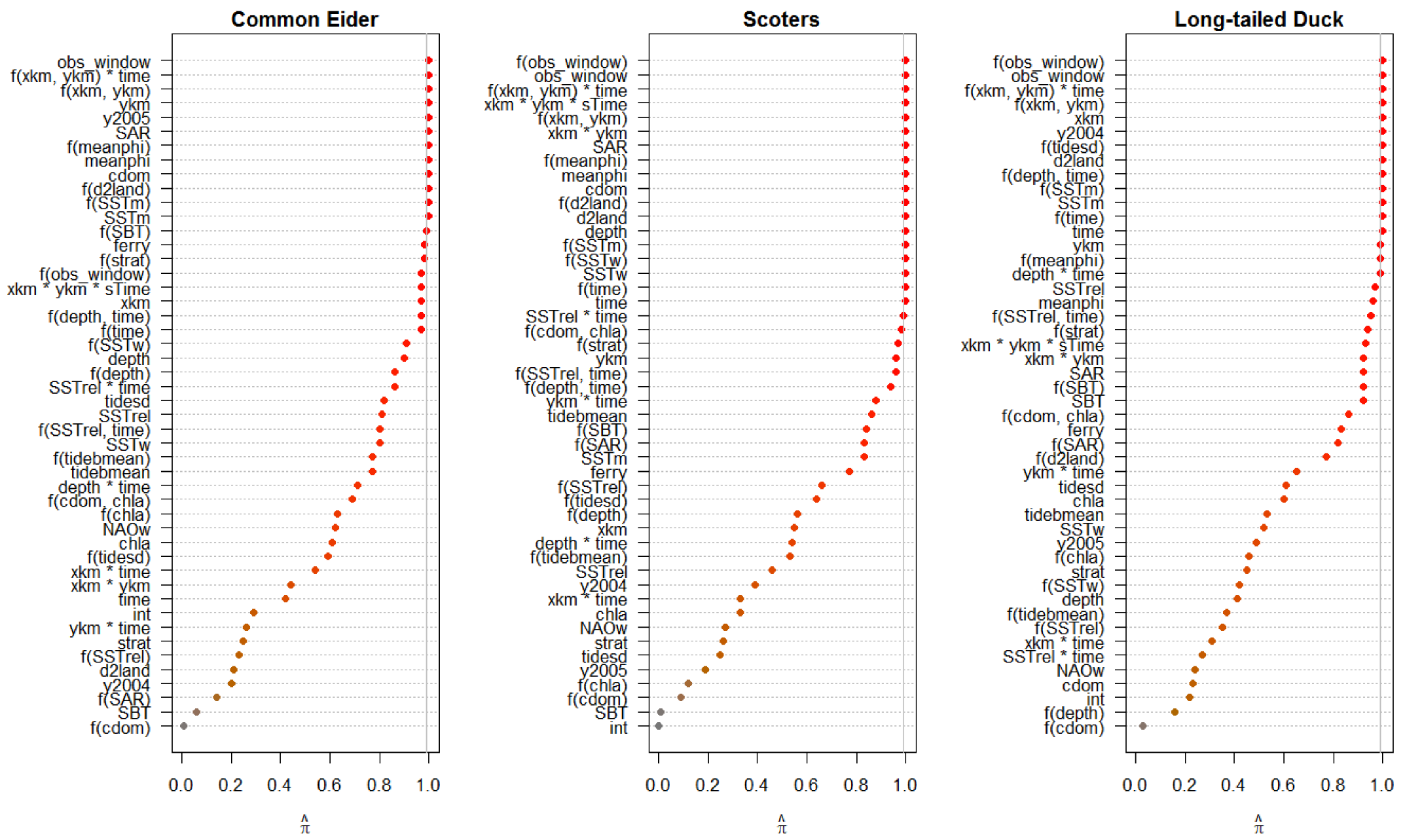

Figure S1 Stability selection using complementary pairs subsampling and unimodality assumption for sea duck occupancy models. The number of selected base-learners in each model run was set to $q = 35$. Base-learners with selection frequencies above the threshold (π_{thr}; vertical gray line) were considered stable with upper bound PFER $= 3$.

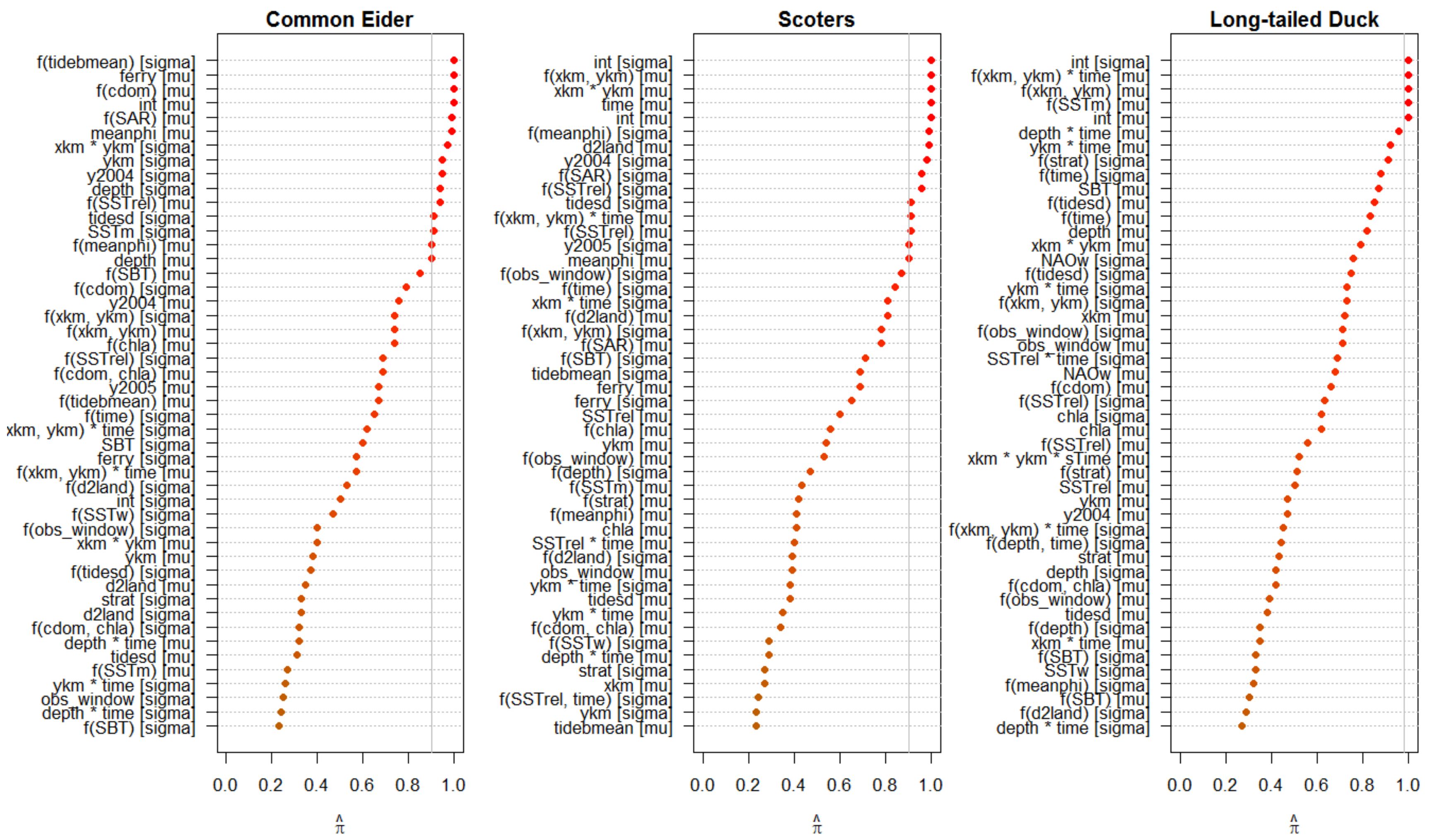

Figure S2 Stability selection using complementary pairs subsampling and unimodality assumption for sea duck conditional count models. The number of selected base-learners in each model run was set to $q = 35$. Base-learners with selection frequencies above the threshold (π_{thr}; vertical gray line) were considered stable with upper bound PFER $= 6$. Only the top 48 (of 96 total) base-learners are illustrated. Brackets indicate the parameter (conditional mean, *mu*, or overdispersion, *sigma*) to which the base-learner applies.

References

Hofner, B., Boccuto, L. & Göker, M. (2015). Controlling false discoveries in high-dimensional situations: Boosting with stability selection. *BMC Bioinformatics*, **16**, 144.

Meinshausen, N. & Bühlmann, P. (2010). Stability selection (with discussion). *Journal of the Royal Statistical Society: Series B (Statistical Methodology)*, **72**, 417–473.

Shah, R.D. & Samworth, R.J. (2013). Variable selection with error control: Another look at stability selection. *Journal of the Royal Statistical Society: Series B (Statistical Methodology)*, **75**, 55–80.
