# Supplementary material for: Modeling spatiotemporal abundance of mobile wildlife in highly variable environments using boosted GAMLSS hurdle models
Source: Ecol Evol. 2019 Feb 14;9(5):2346–64. doi: 10.1002/ece3.4738 (PMC6405508; doi:10.1002/ece3.4738)
Supplement: Supplementary file 2 [file ECE3-9-2346-s002.pdf]

S.2 Early stopping

Occupancy models

All occupancy models converged to the maximum likelihood estimates (i.e., did not stop early; Figure S3). Failure to stop early sometimes happens in data sets with many observations and strong effects (see comment of Kneib in Bühlmann *et al.* 2014). This suggests that the effects of the environmental variables on sea duck occupancy are rather complex.

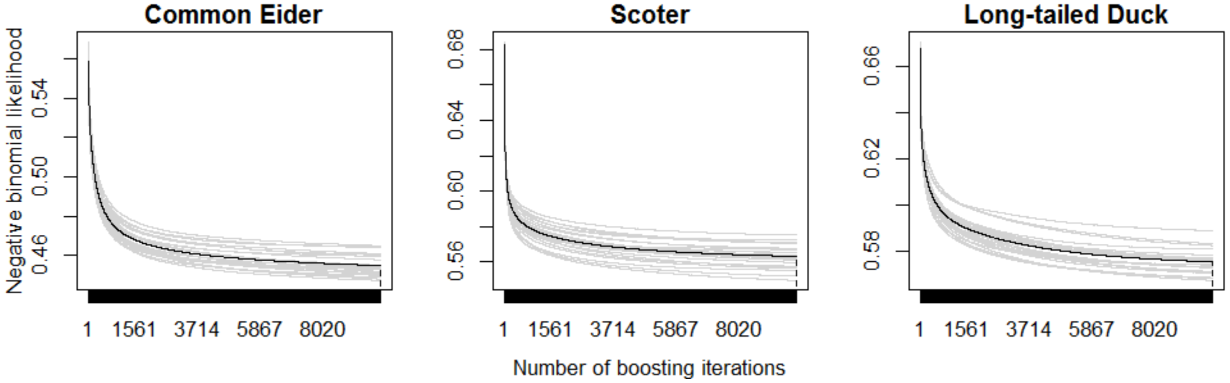

Figure S3 Bootstrapped out-of-bag empirical risk in sea duck occupancy models based on 25-fold subsampling. Gray lines indicate the out-of-bag risk on each subsample and the black line indicates the average out-of-bag risk; the optimal iteration is indicated by the dashed vertical line.

Count models

In contrast to occupancy model, bootstrapping prescribed early stopping for both parameters in all count models (Figure S4).

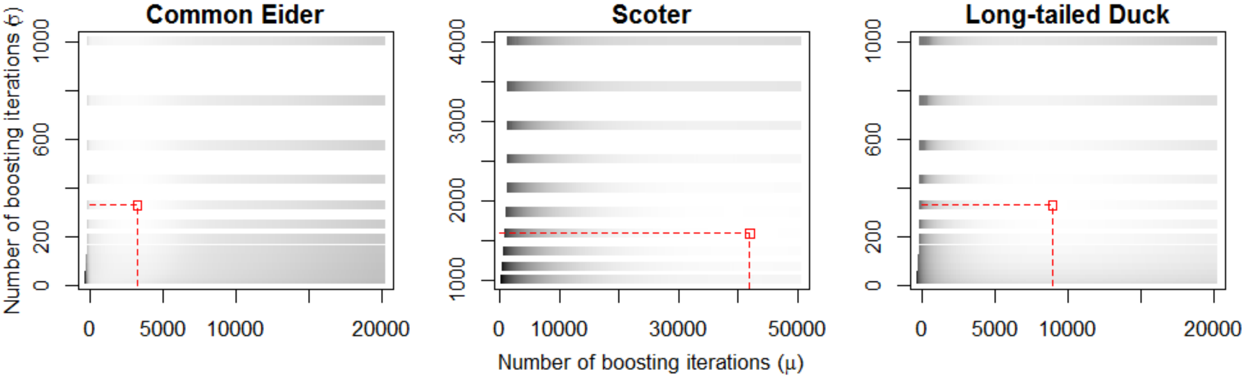

Figure S4 Bootstrapped out-of-bag empirical risk in sea duck conditional count models based on 25-fold subsampling. Lighter colors indicate lower average out-of-bag risk (over the 25 samples) for a given combination of m_{stop}-values for μ and σ; the optimal combination is indicated by the red square.

References

Bühlmann, P., Gertheiss, J., Hieke, S., Kneib, T., Ma, S., Schumacher, M., Tutz, G., Wang, C.-Y., Wang, Z. & Ziegler, A. (2014). Discussion of 'the evolution of boosting algorithms' and 'extending statistical boosting'. *Methods of Information in Medicine*, **53**, 436–445.
