# Supplementary material for: Modeling spatiotemporal abundance of mobile wildlife in highly variable environments using boosted GAMLSS hurdle models
Source: Ecol Evol. 2019 Feb 14;9(5):2346–64. doi: 10.1002/ece3.4738 (PMC6405508; doi:10.1002/ece3.4738)

# S.3 Stable covariate effects


Common Eider . . . . . 2
Occupancy . . . . . 2
Conditional mean . . . . . 11
Conditional overdispersion . . . . . 17
Black, Surf, and White-winged Scoter . . . . . 23
Occupancy . . . . . 23
Conditional mean . . . . . 34
Conditional overdispersion . . . . . 39
Long-tailed Duck . . . . . 45
Occupancy . . . . . 45
Conditional mean . . . . . 54
Conditional overdispersion . . . . . 57


Here we provide marginal functional plots of the relationships between stable covariates and the occupancy, conditional mean abundance, and conditional overdispersion of abundance of sea ducks in Nantucket Sound, Massachusetts, USA. By 'marginal' we mean holding all other variables at their mean or reference values. Covariate plots are ordered roughly in descending order of the magnitude of their influence on the additive predictor in each model (or model parameter for count models). Vertical lines along the $x$-axis (i.e., rug plots) indicate observed covariate values. Covariates (and any abbreviations) are defined in detail in Table 1 on the manuscript; only effects selected to be stable are depicted (see Supplemental Information S1).

## Common Eider

### Occupancy

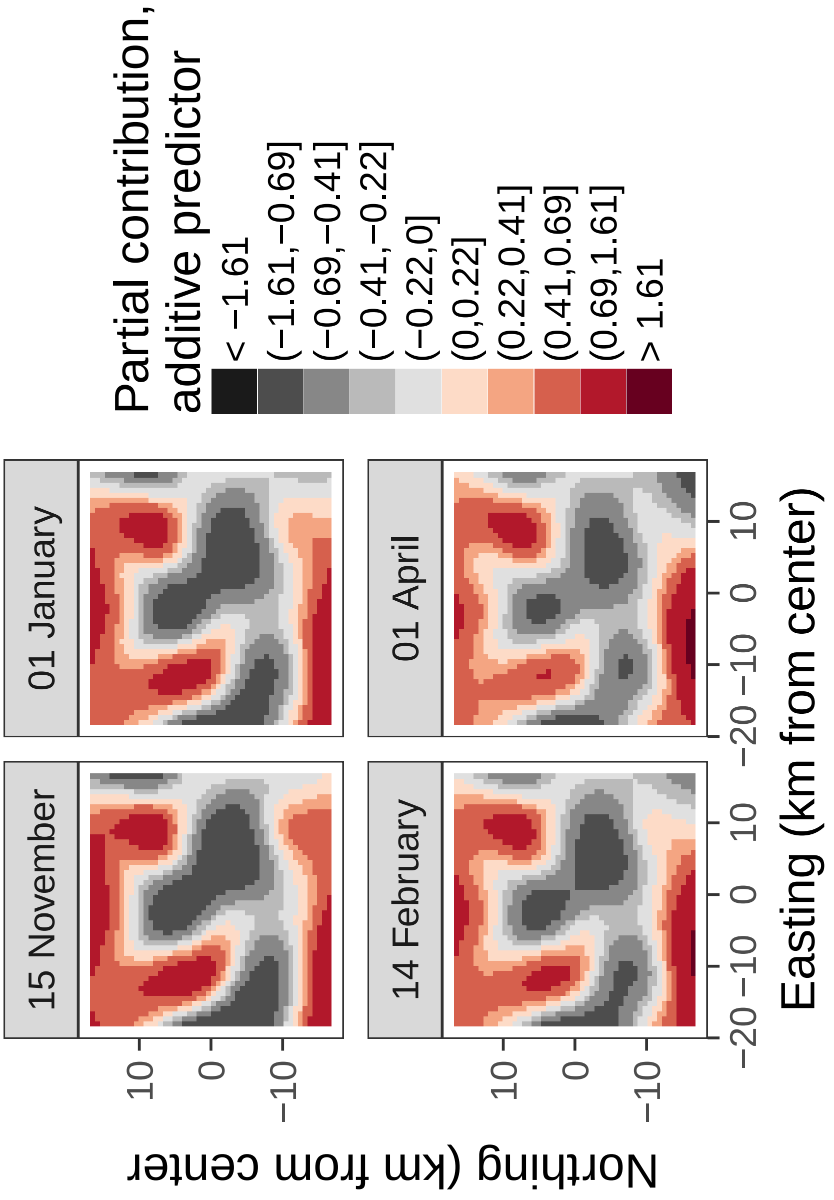

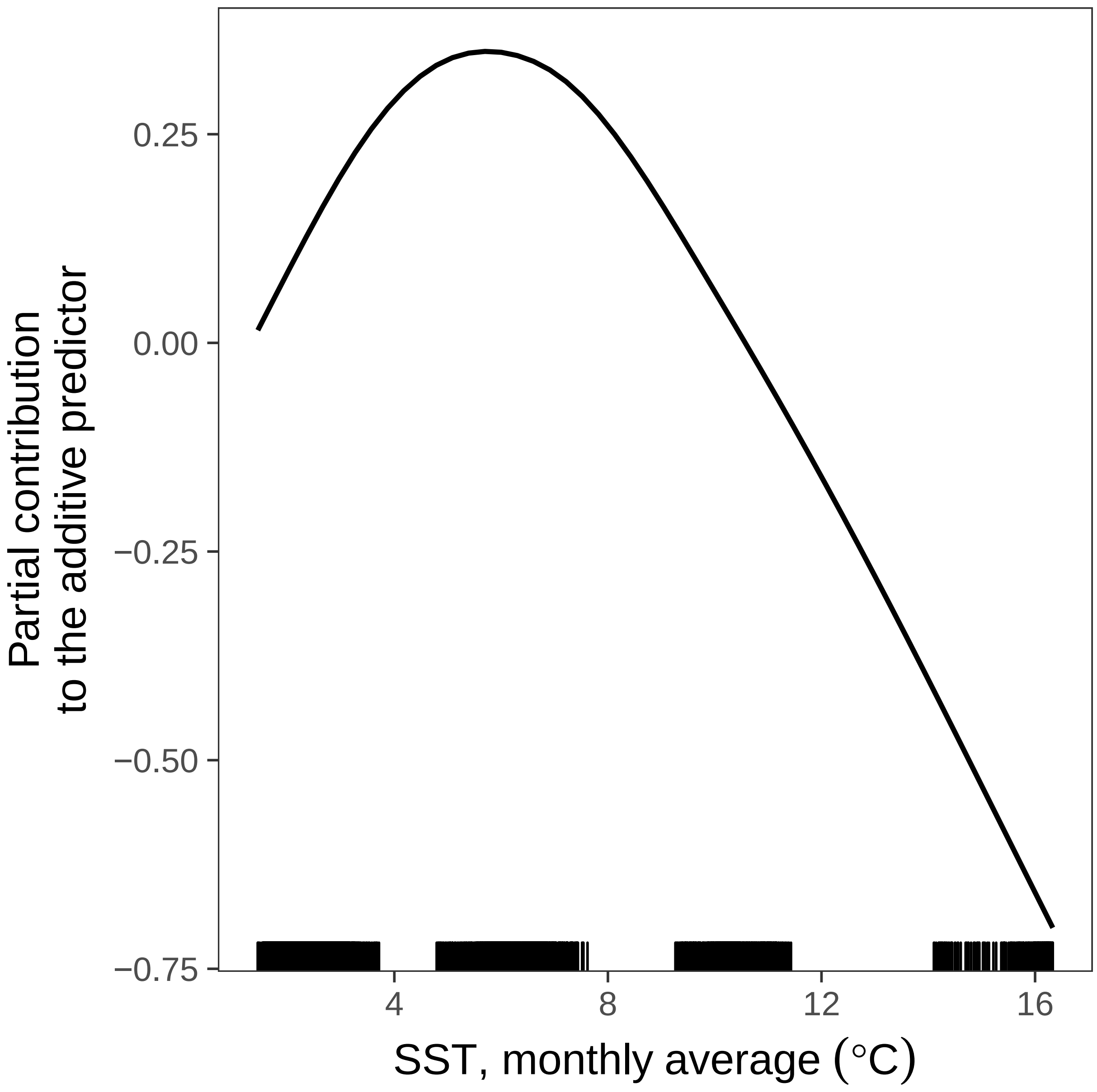
0.25
0.00
−0.25
−0.50
−0.75
Partial contribution
to the additive predictor
4
8
12
16
SST, monthly average (°C)

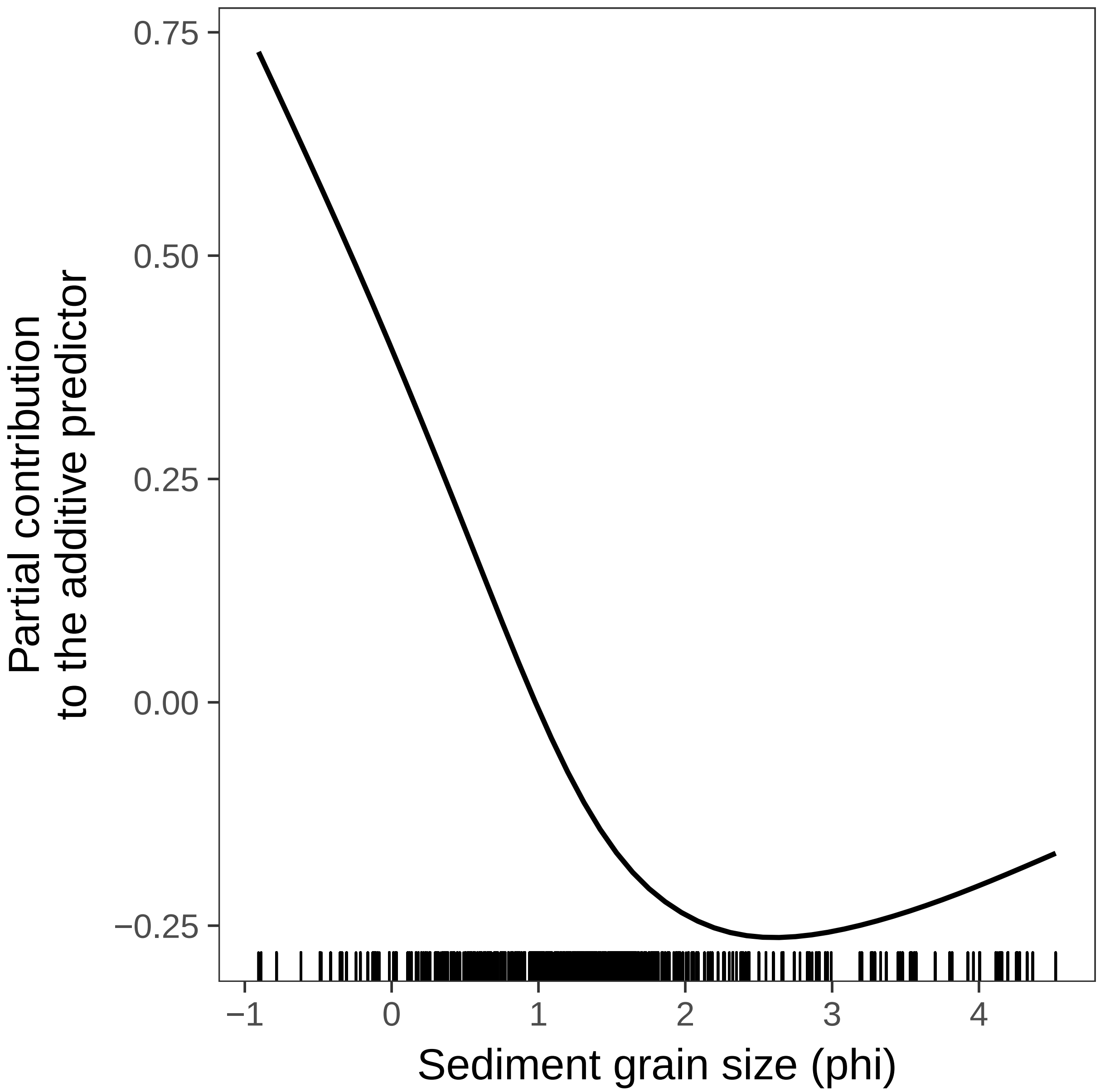
0.75
0.50
0.25
0.00
−0.25
Partial contribution
to the additive predictor
−1
0
1
2
3
4
Sediment grain size (phi)

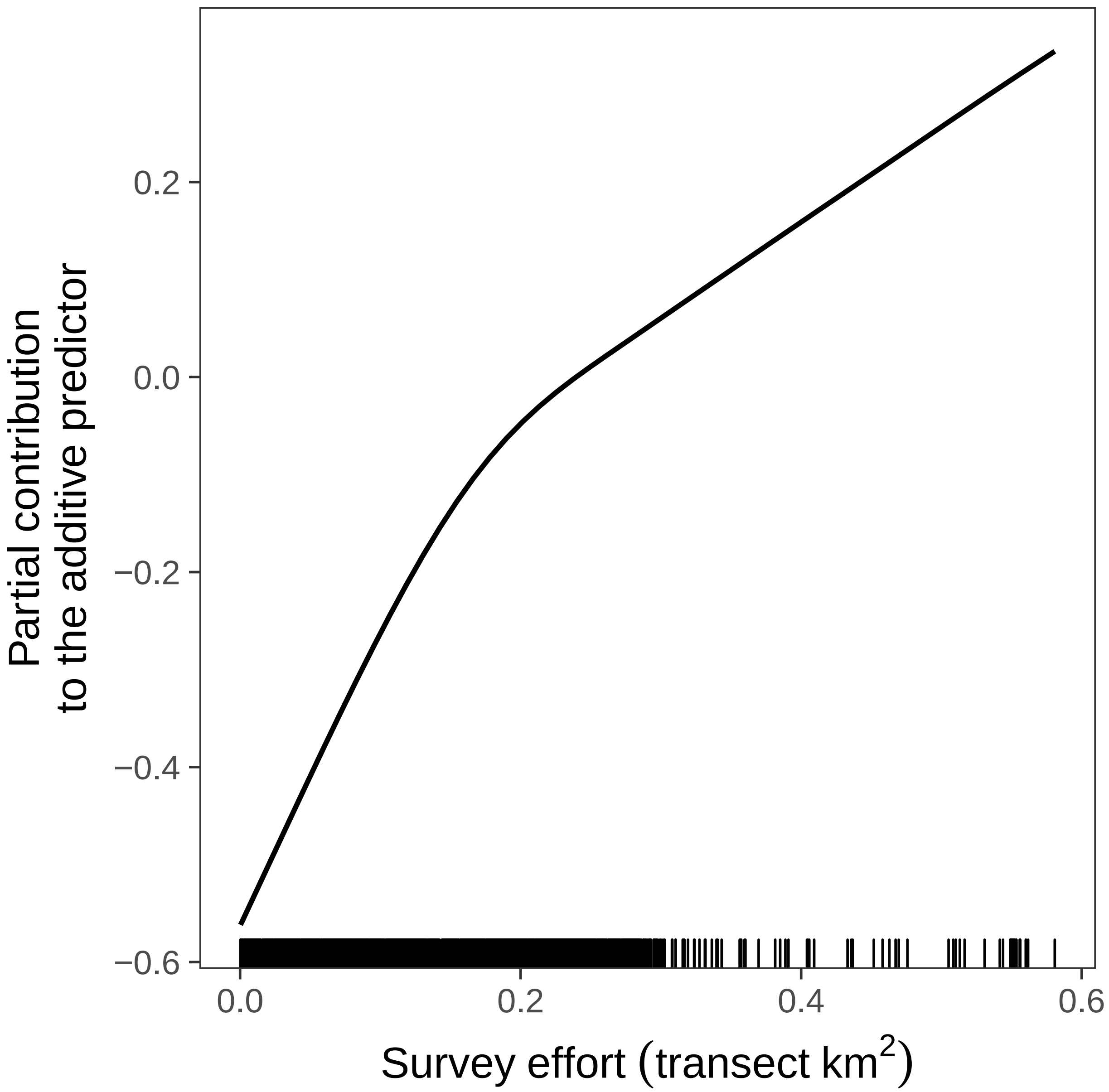

Partial contribution
to the additive predictor
0.2
0.0
−0.2
−0.4
−0.6
0.0
0.2
0.4
0.6
Survey effort (transect km2)

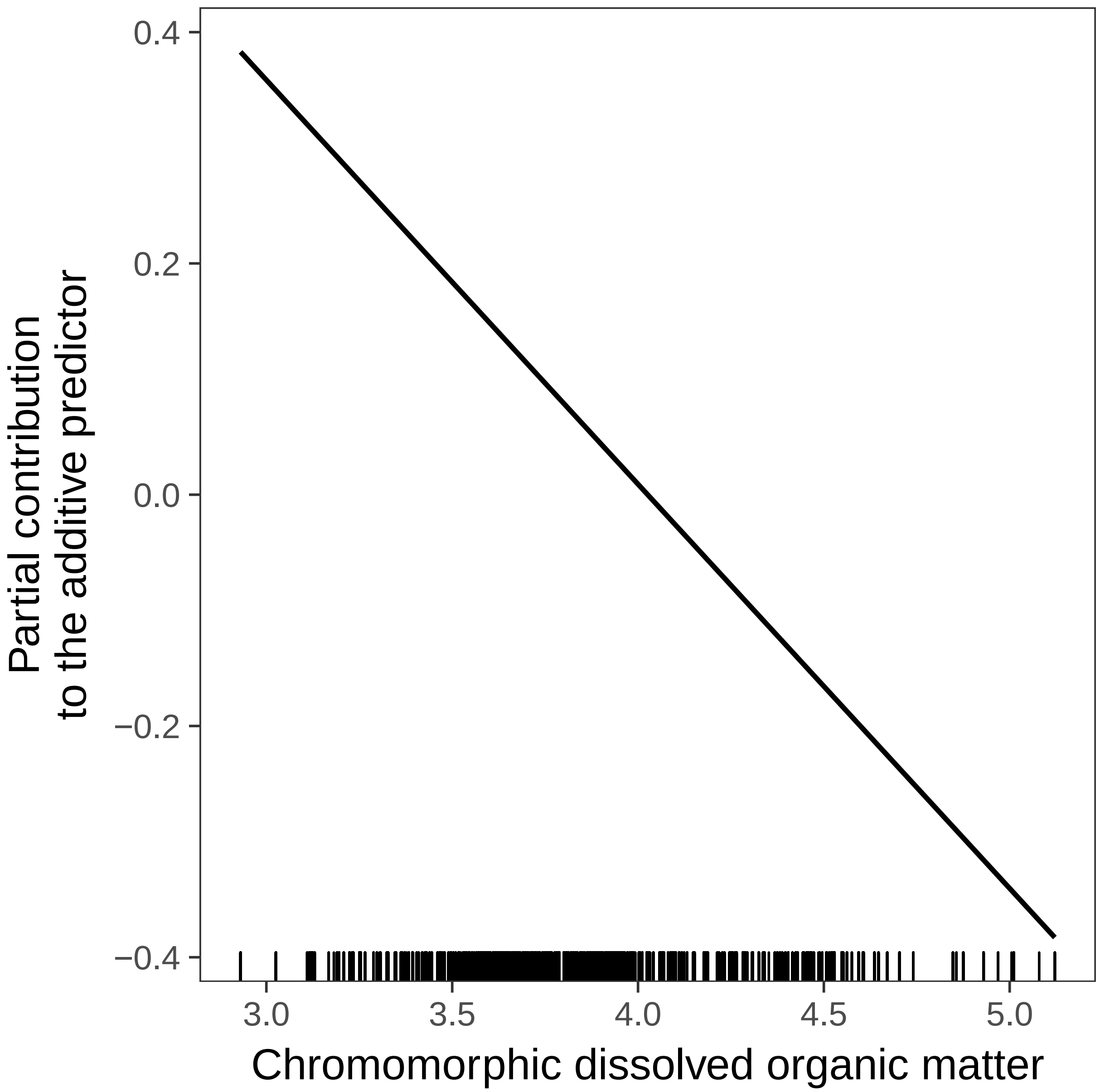
0.4
0.2
0.0
−0.2
−0.4
Partial contribution
to the additive predictor
3.0
3.5
4.0
4.5
5.0
Chromomorphic dissolved organic matter

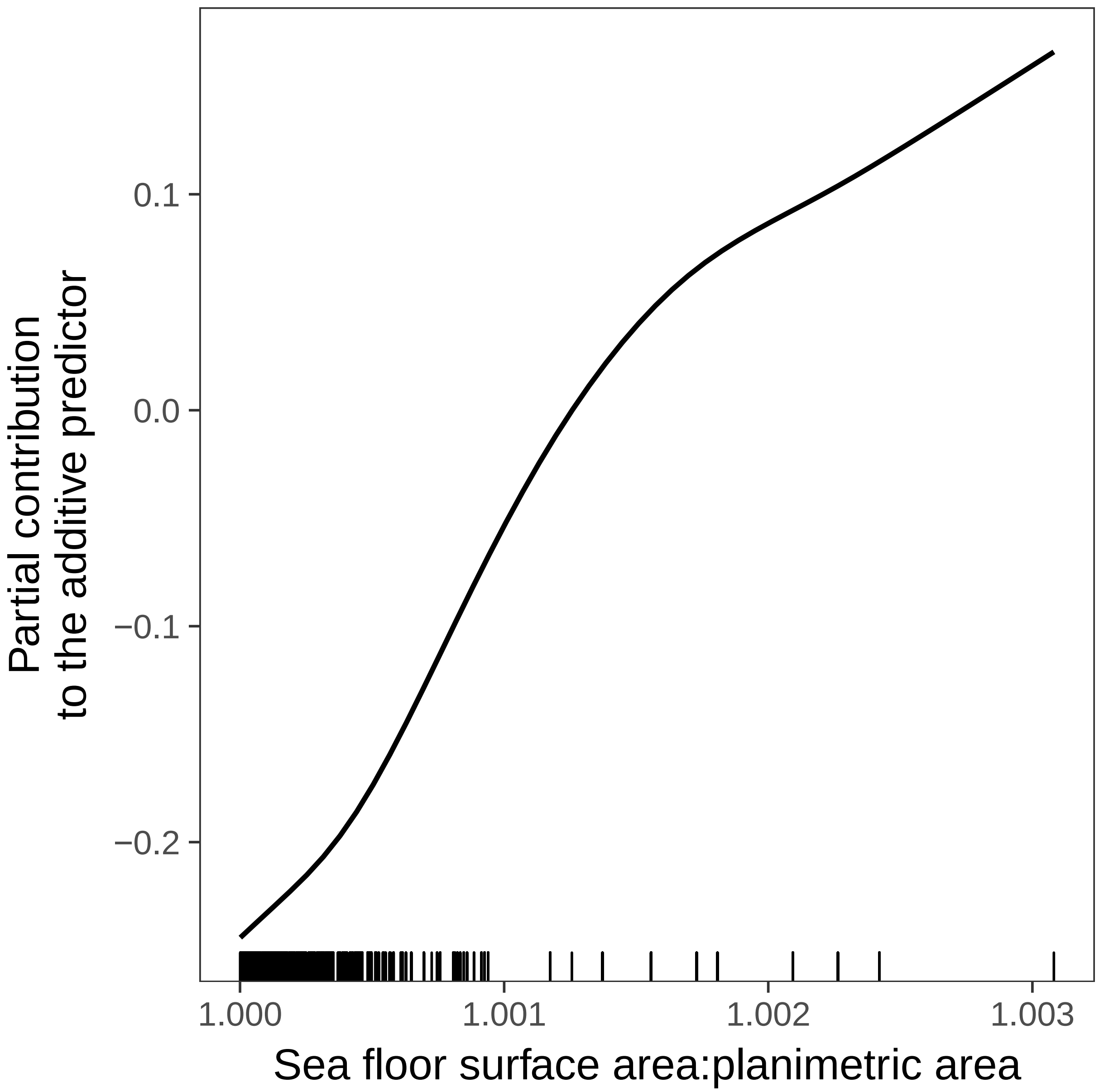
Partial contribution
to the additive predictor
0.1
0.0
−0.1
−0.2
1.000
1.001
1.002
1.003
Sea floor surface area:planimetric area

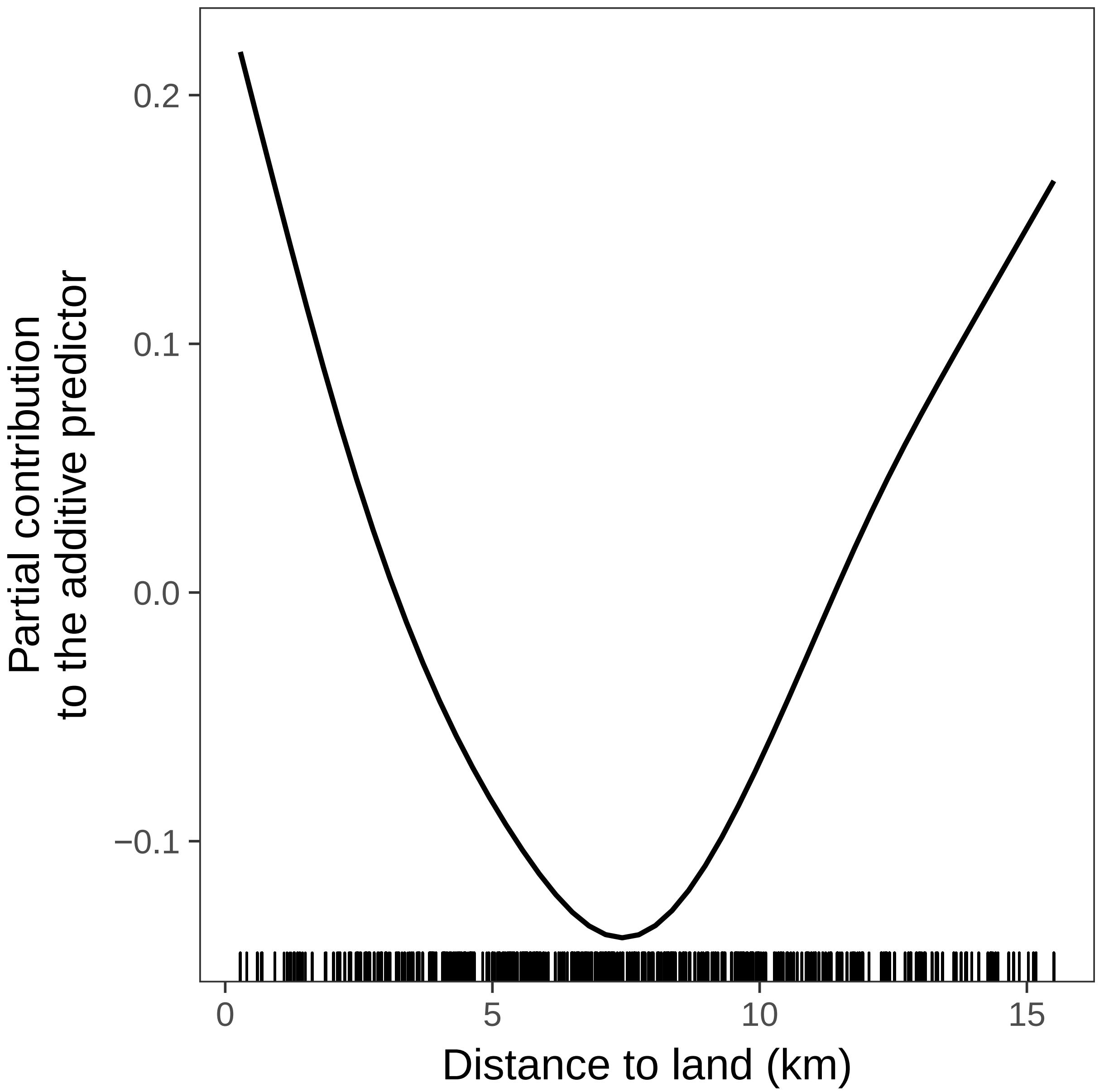
Partial contribution
to the additive predictor
0.2
0.1
0.0
−0.1
0
5
10
15
Distance to land (km)

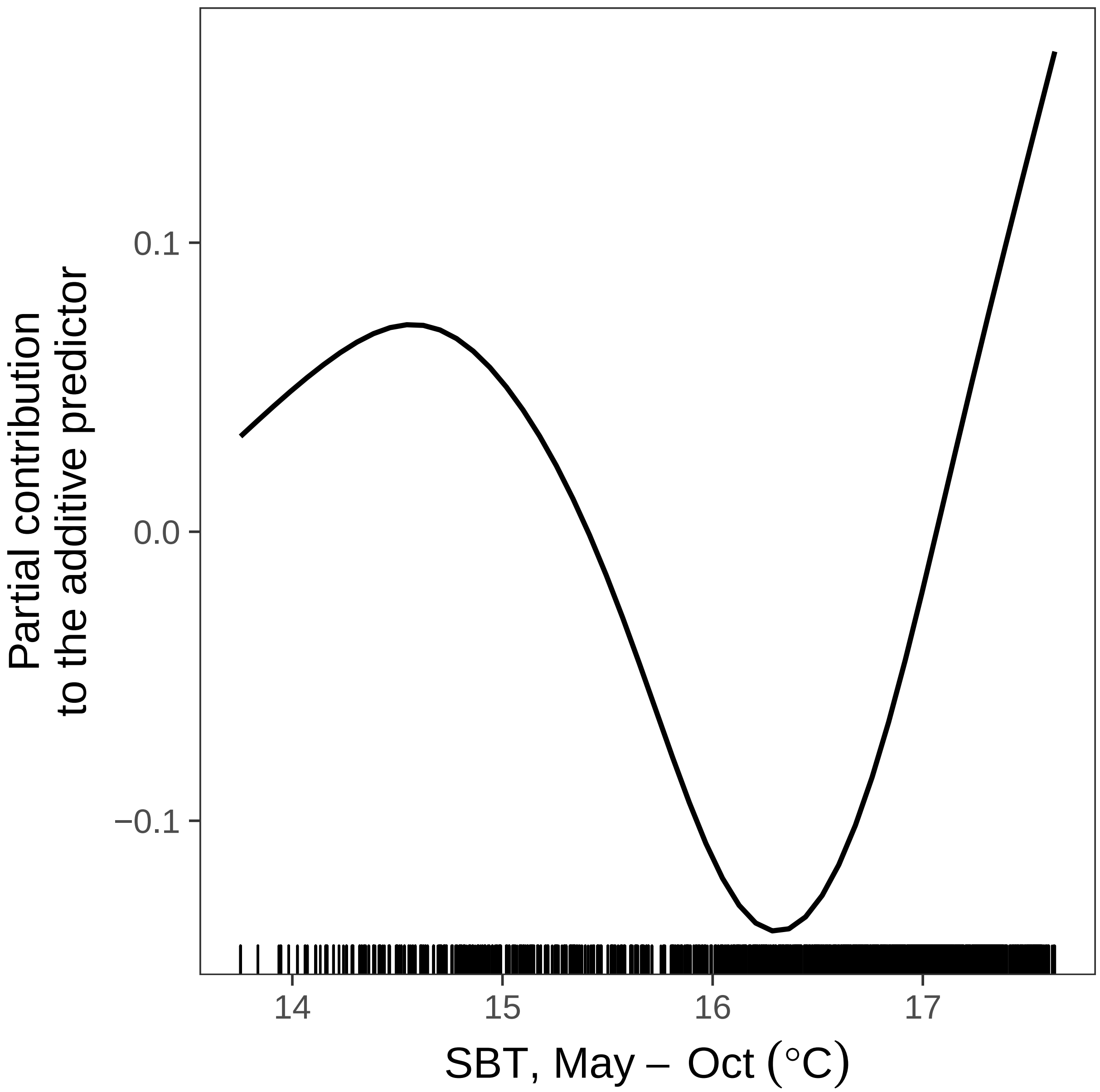

Partial contribution
to the additive predictor
0.1
0.0
−0.1
14
15
16
17
SBT, May – Oct (°C)

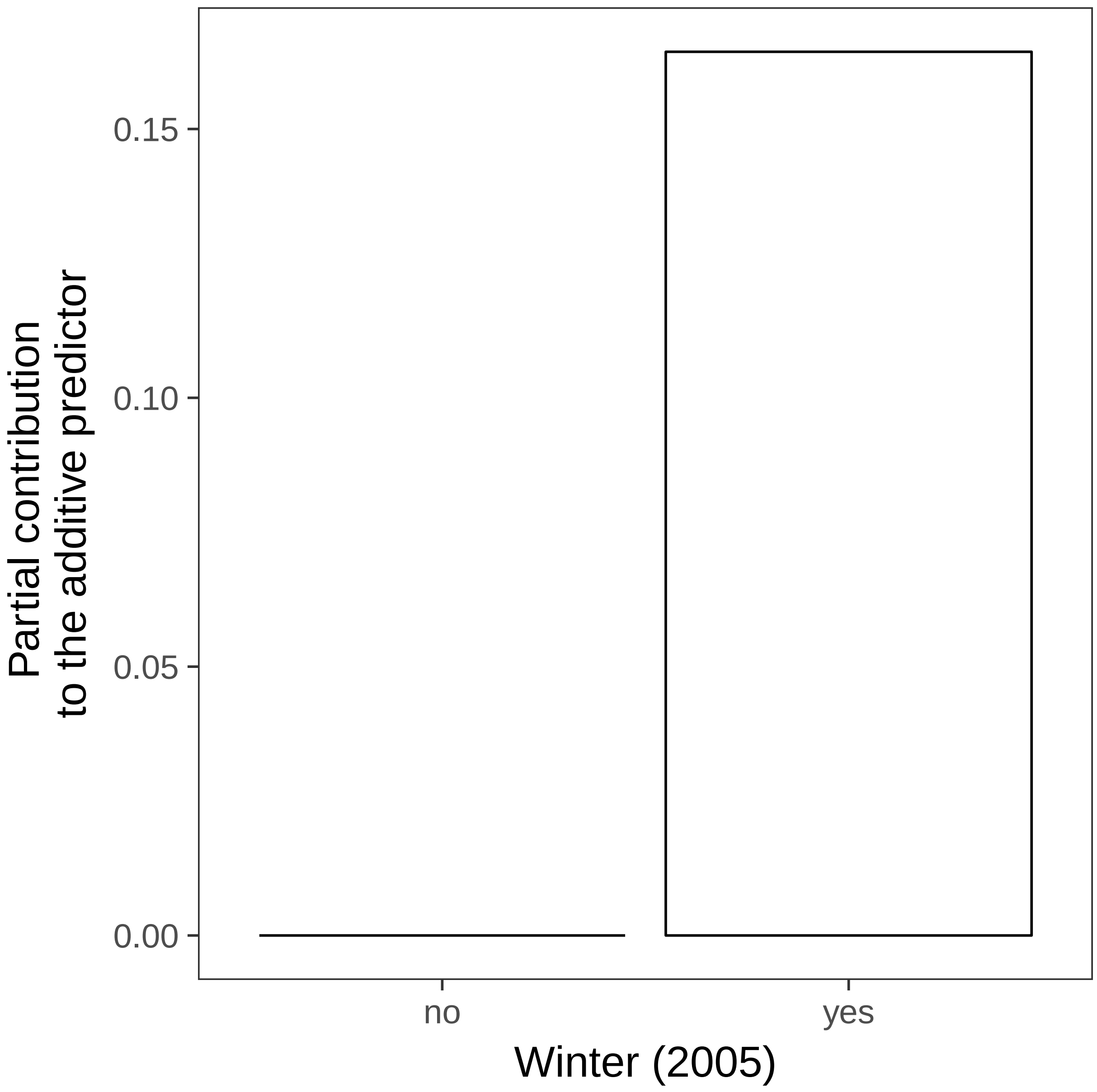
Partial contribution
to the additive predictor
0.15
0.10
0.05
0.00
no
yes
Winter (2005)

**Conditional mean**

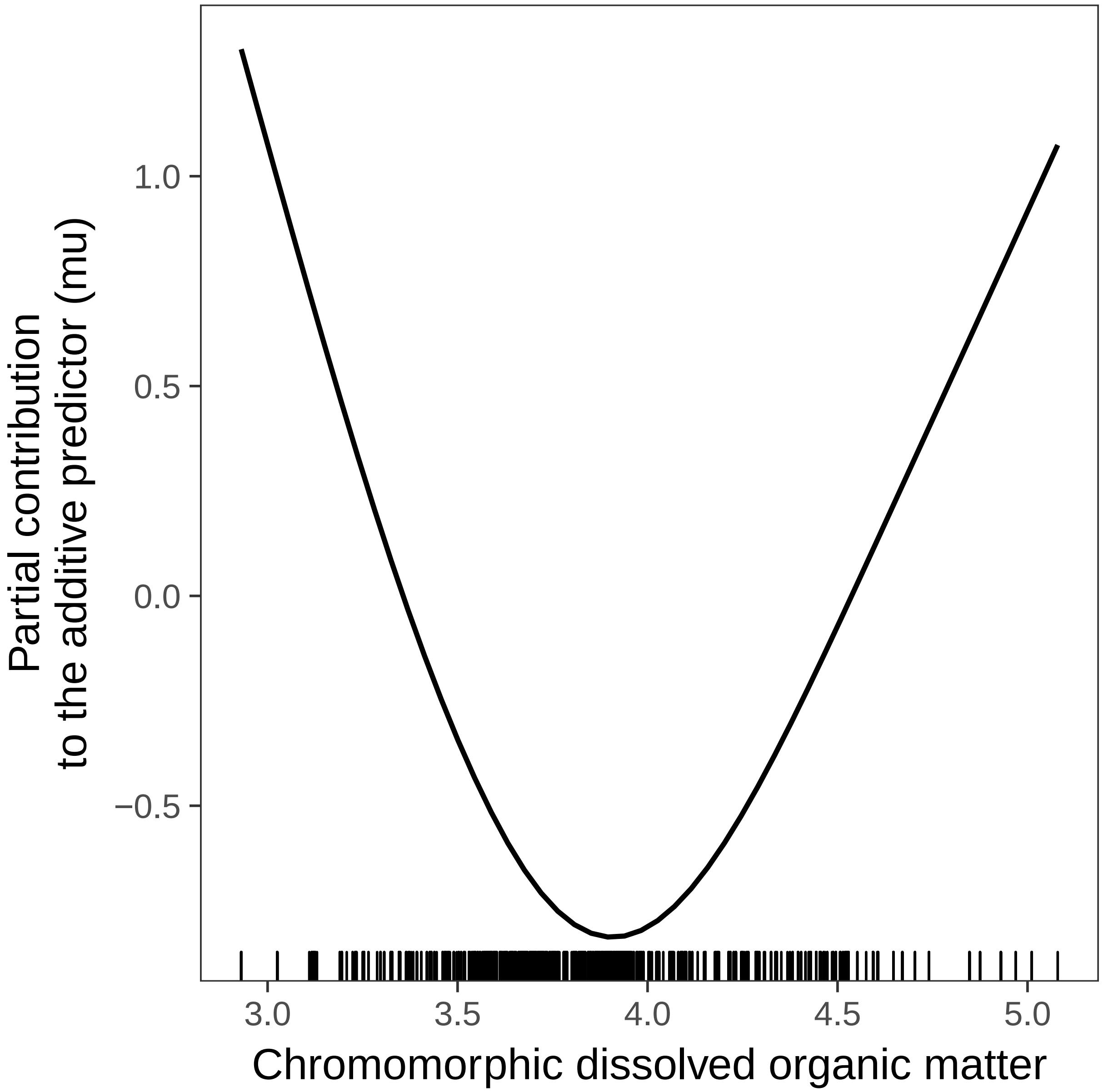

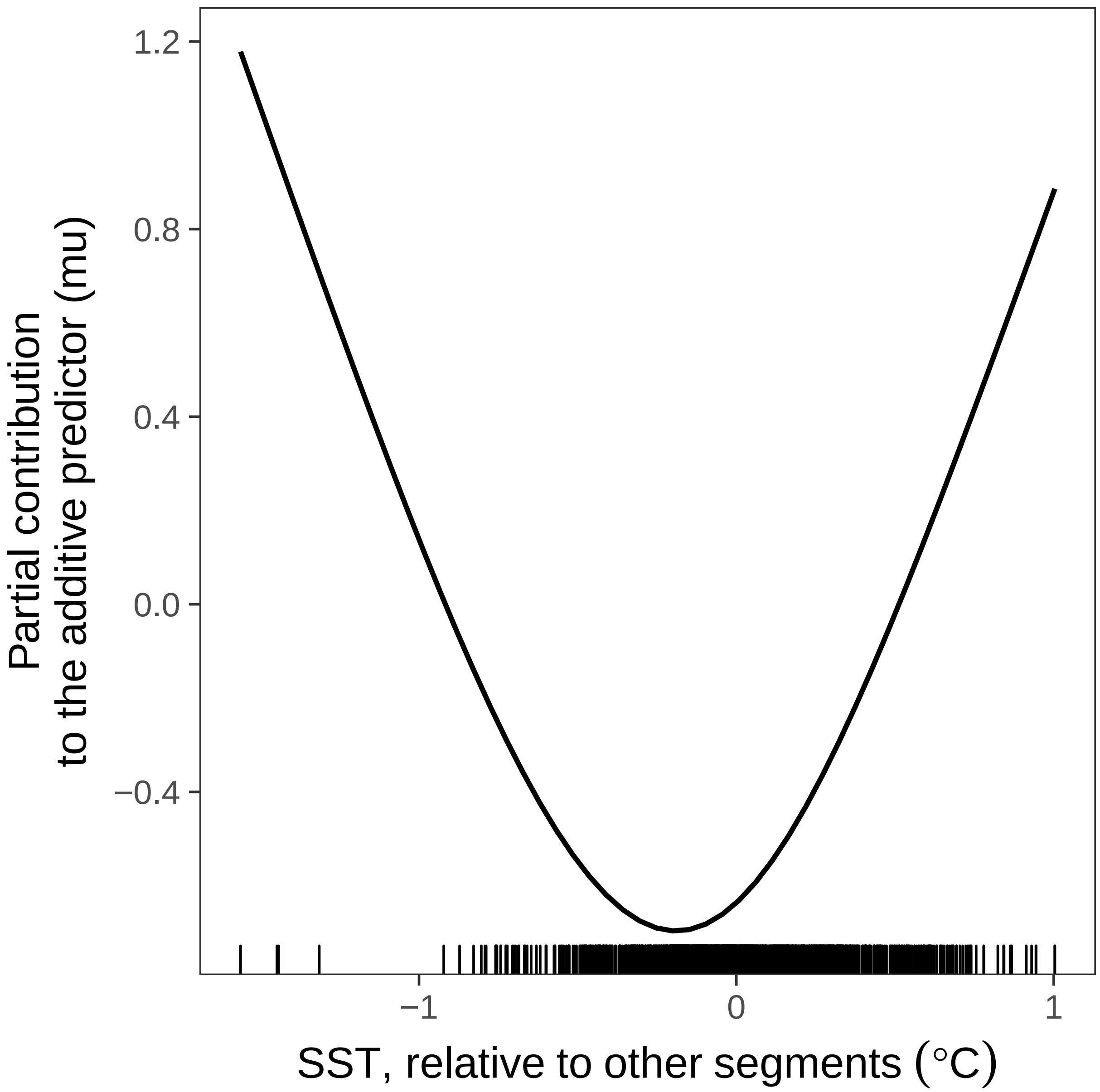
1.2
0.8
0.4
0.0
−0.4
Partial contribution
to the additive predictor (mu)
−1
0
1
SST, relative to other segments (°C)

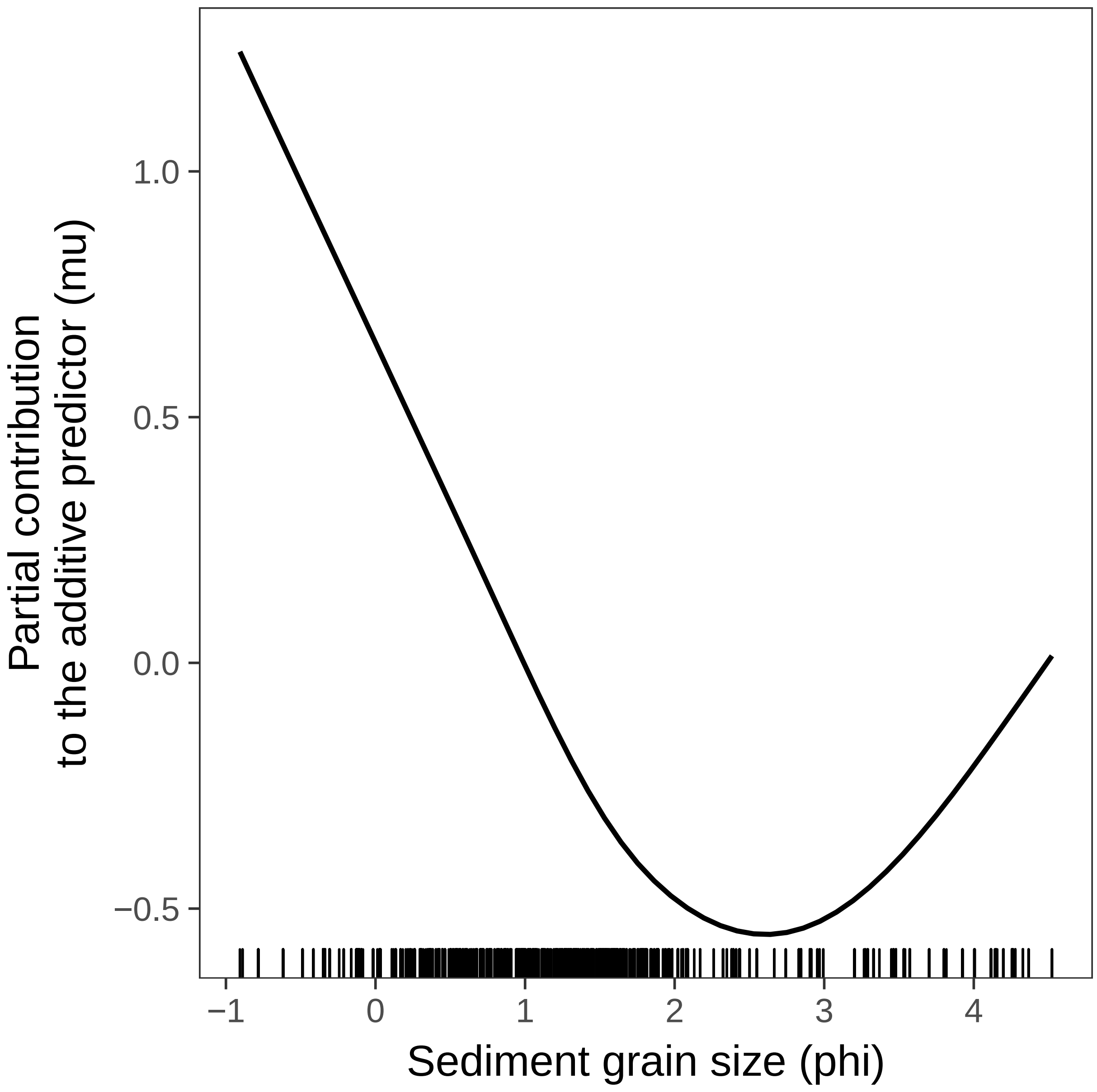
Partial contribution
to the additive predictor (mu)
1.0
0.5
0.0
−0.5
−1
0
1
2
3
4
Sediment grain size (phi)

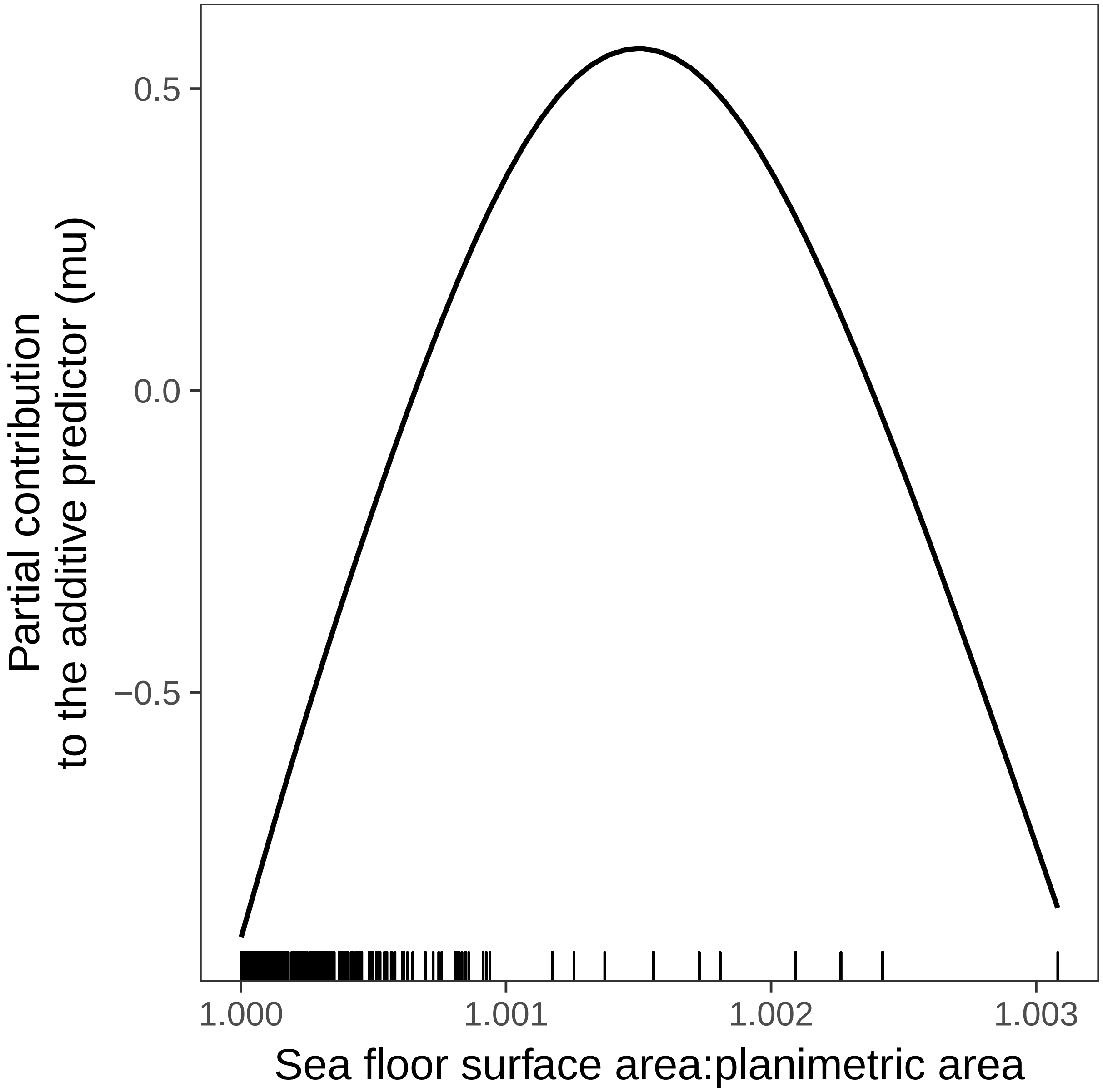

Partial contribution
to the additive predictor (mu)
0.5
0.0
−0.5
1.000
1.001
1.002
1.003
Sea floor surface area:planimetric area

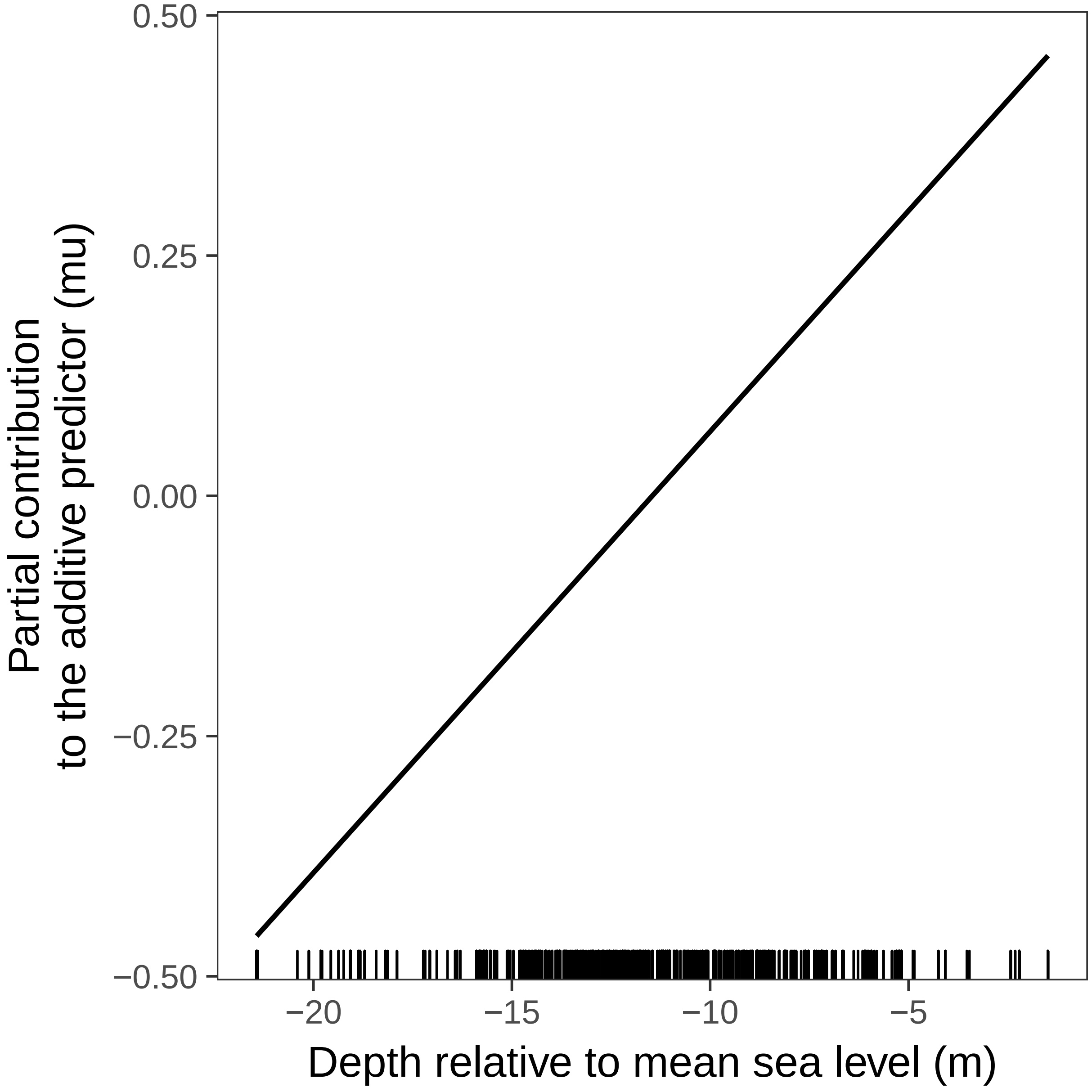

Partial contribution
to the additive predictor (mu)
0.50
0.25
0.00
−0.25
−0.50
−20
−15
−10
−5
Depth relative to mean sea level (m)

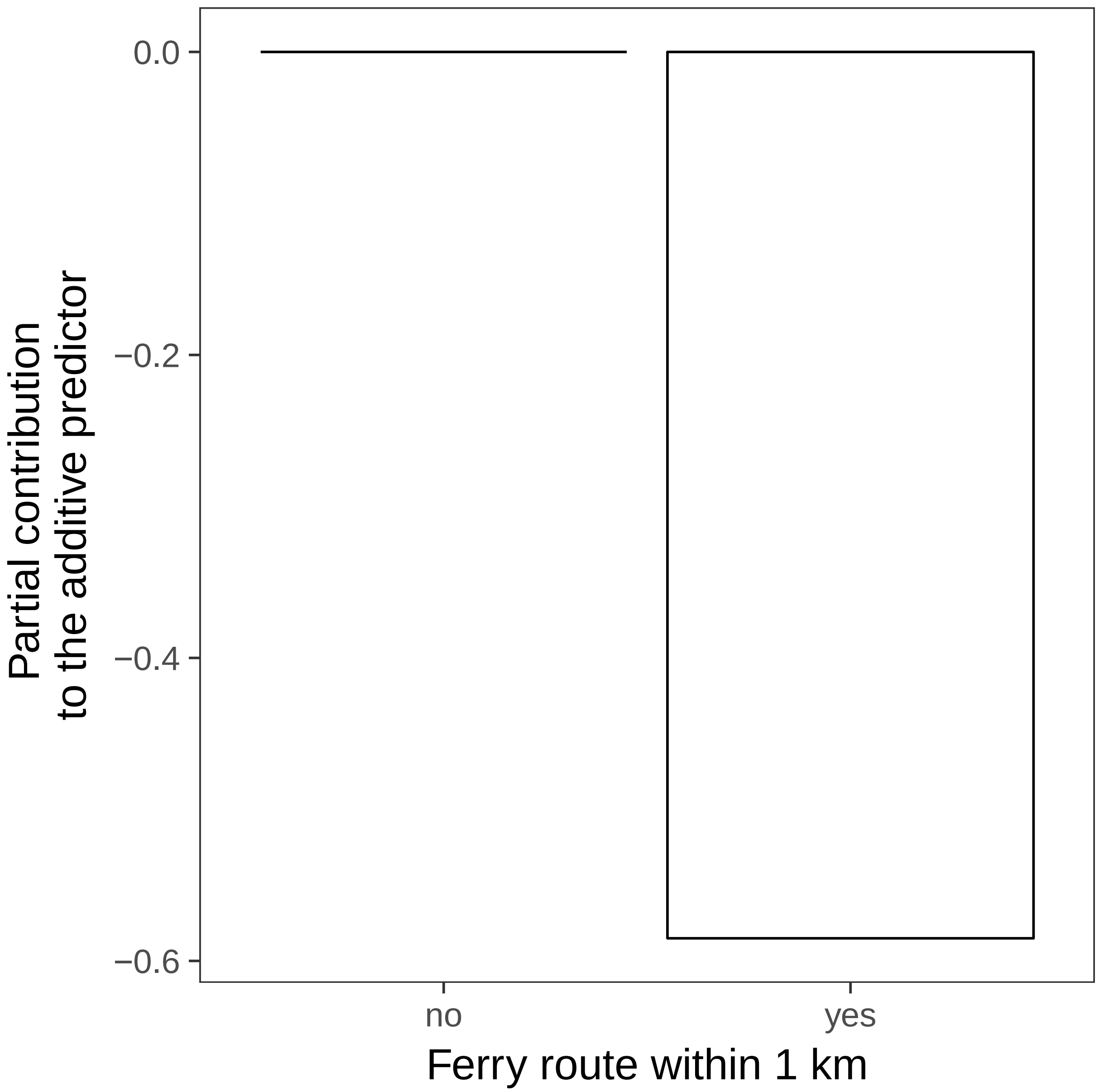
0.0
−0.2
−0.4
−0.6
Partial contribution
to the additive predictor
no
yes
Ferry route within 1 km

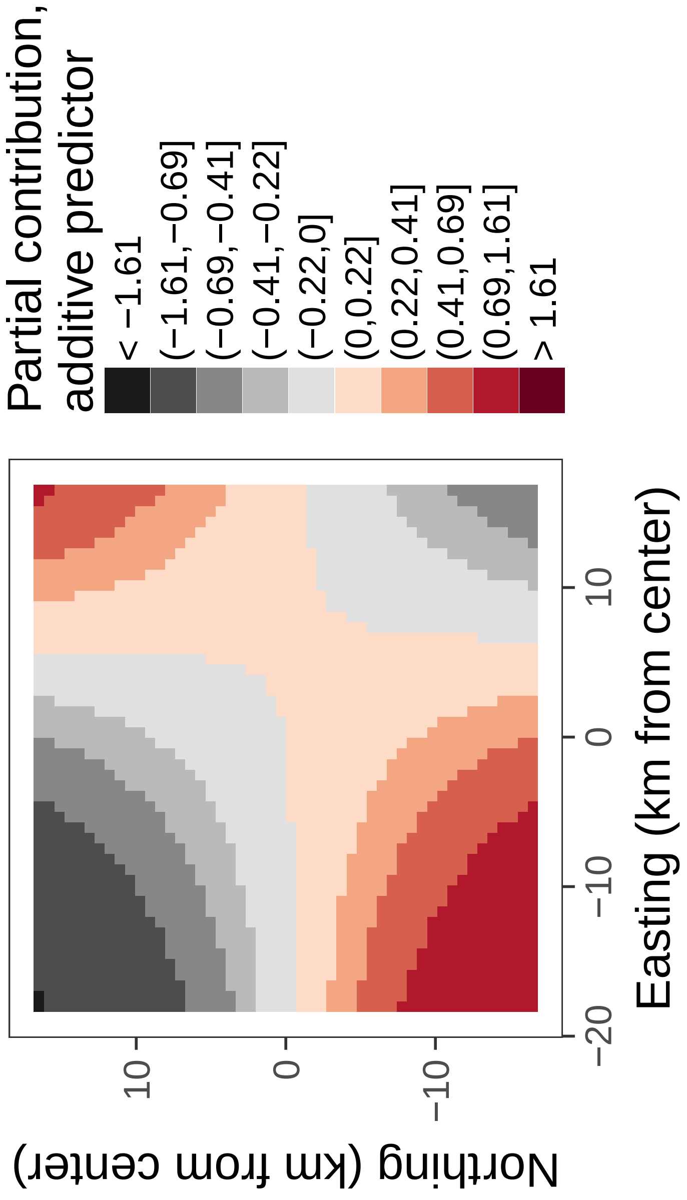
Partial contribution,
additive predictor
< −1.61
(−1.61,−0.69]
(−0.69,−0.41]
(−0.41,−0.22]
(−0.22,0]
(0,0.22]
(0.22,0.41]
(0.41,0.69]
(0.69,1.61]
> 1.61
Easting (km from center)
−20
−10
0
10
Northing (km from center)
10
0
−10

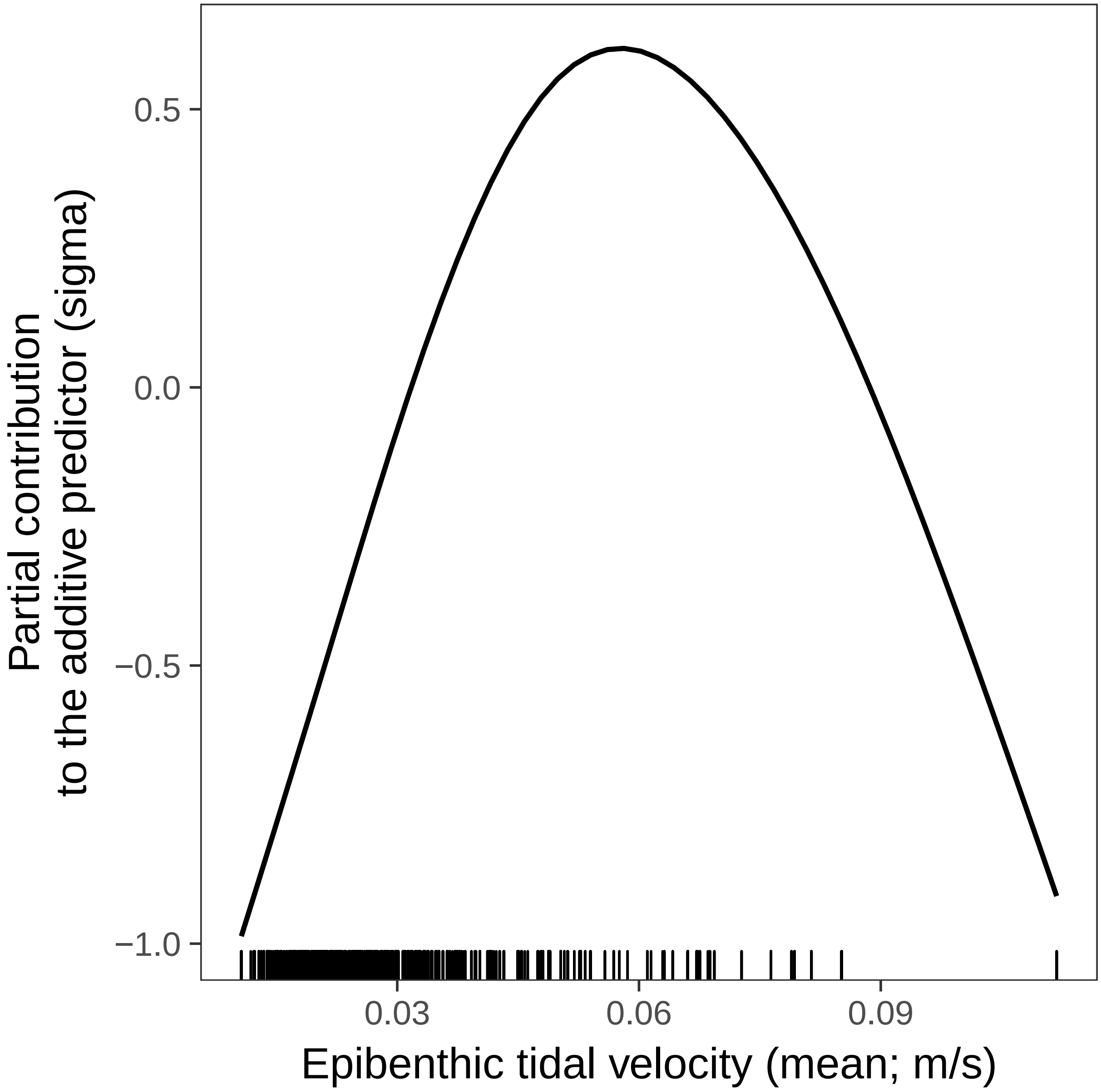

Partial contribution
to the additive predictor (sigma)
0.5
0.0
−0.5
−1.0
0.03
0.06
0.09
Epibenthic tidal velocity (mean; m/s)

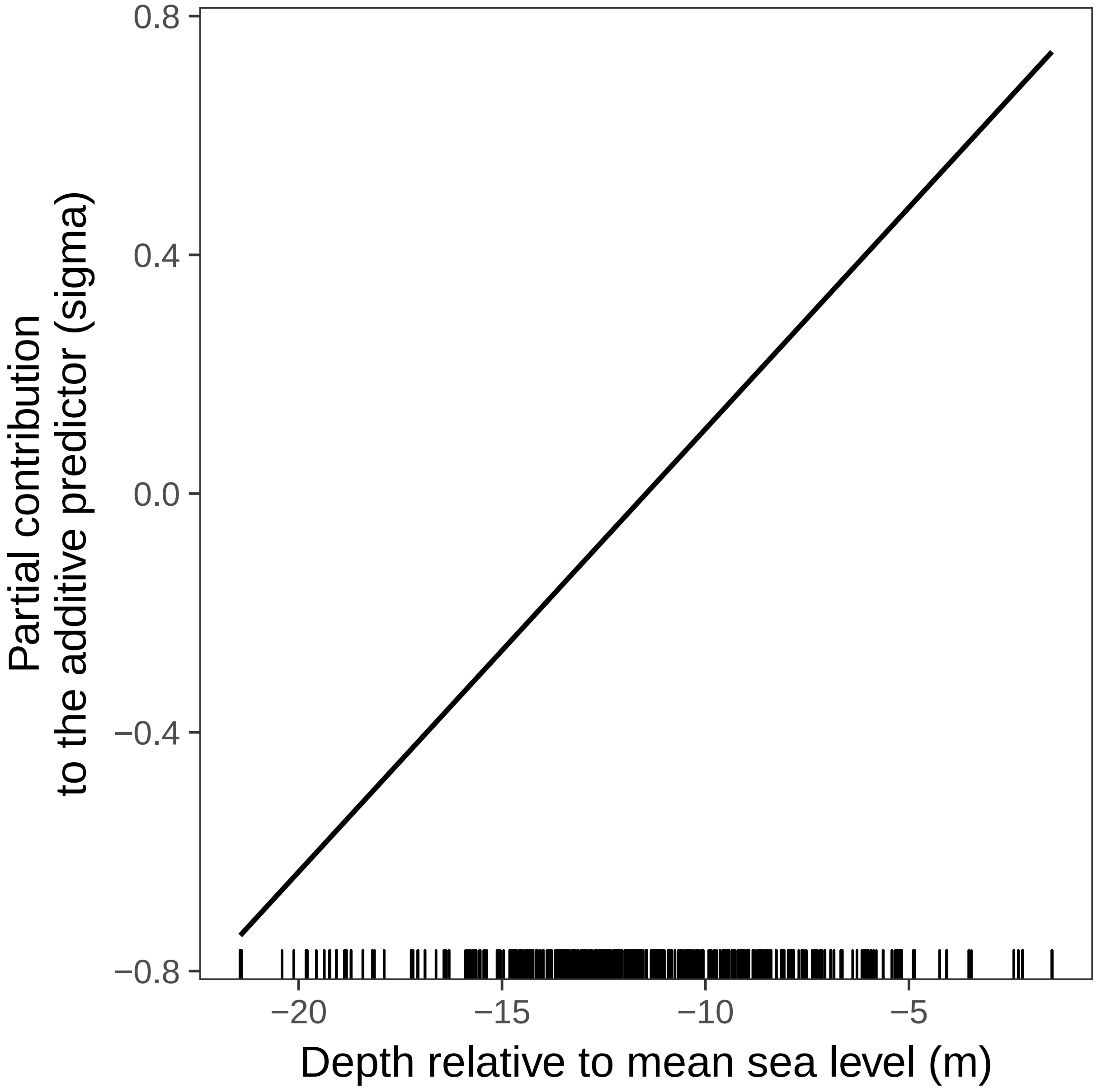

Partial contribution
to the additive predictor (sigma)
0.8
0.4
0.0
−0.4
−0.8
−20
−15
−10
−5
Depth relative to mean sea level (m)

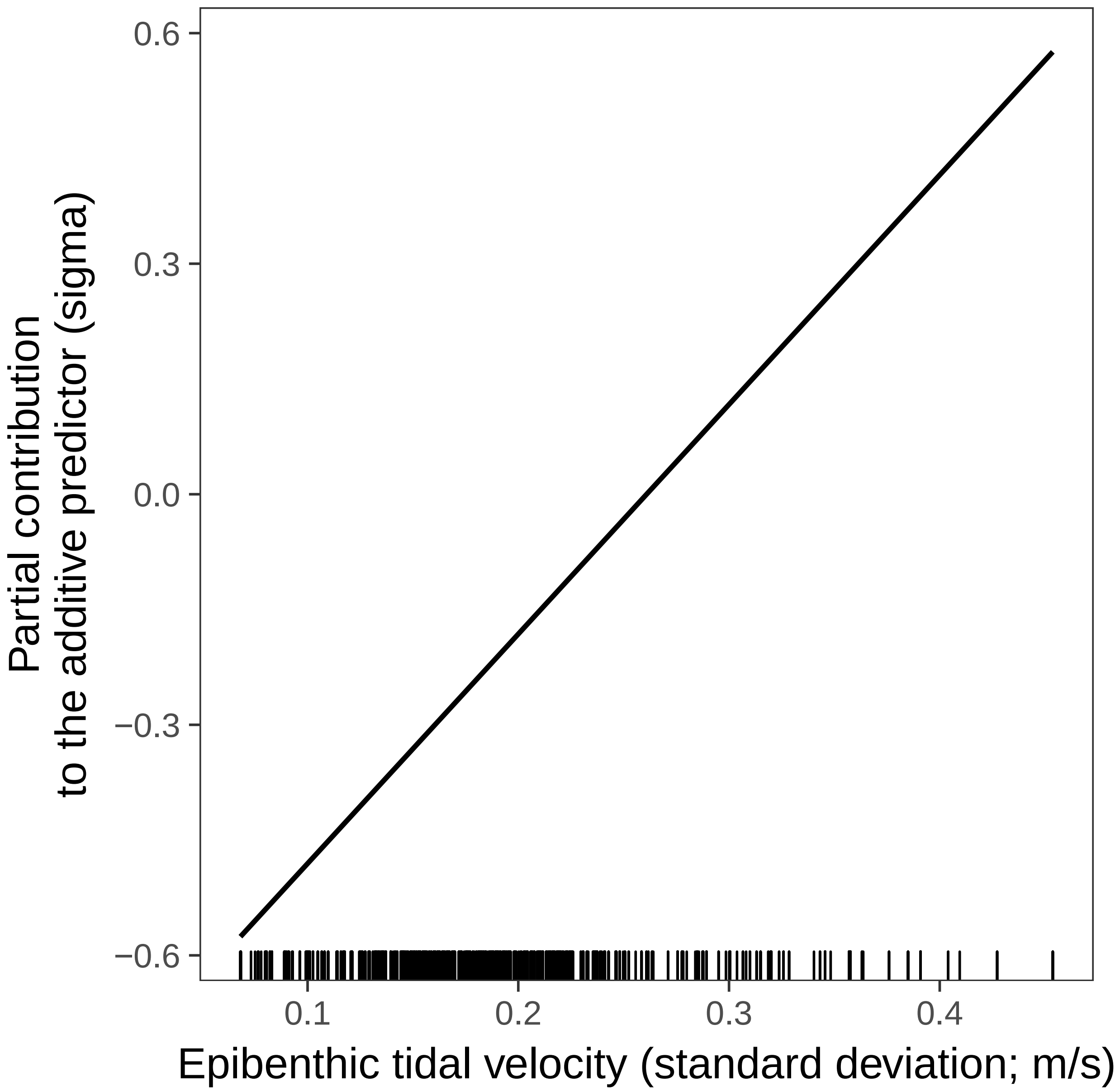
0.6
0.3
0.0
−0.3
−0.6
Partial contribution
to the additive predictor (sigma)
0.1
0.2
0.3
0.4
Epibenthic tidal velocity (standard deviation; m/s)

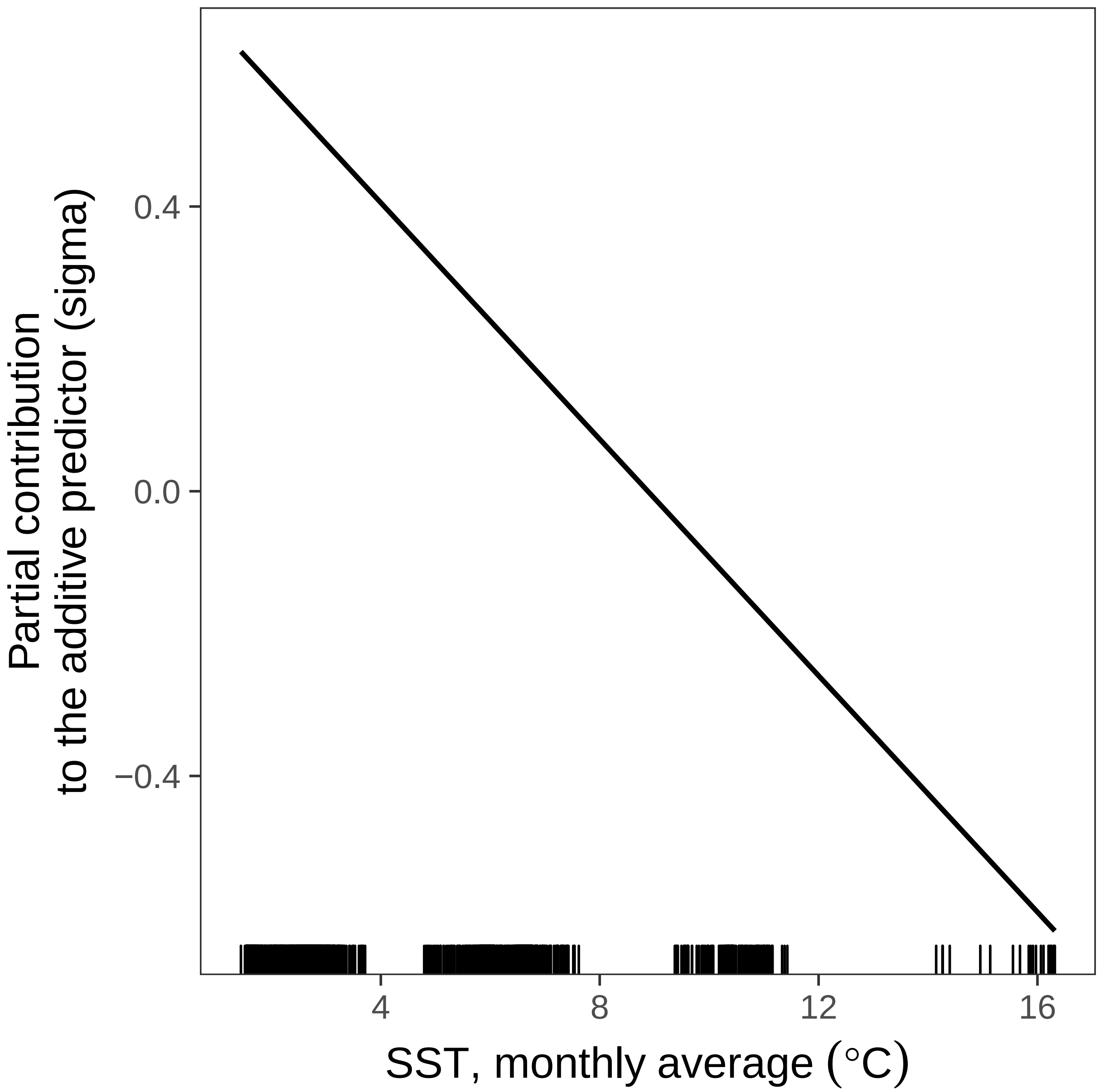
Partial contribution
to the additive predictor (sigma)
0.4
0.0
−0.4
4
8
12
16
SST, monthly average (°C)

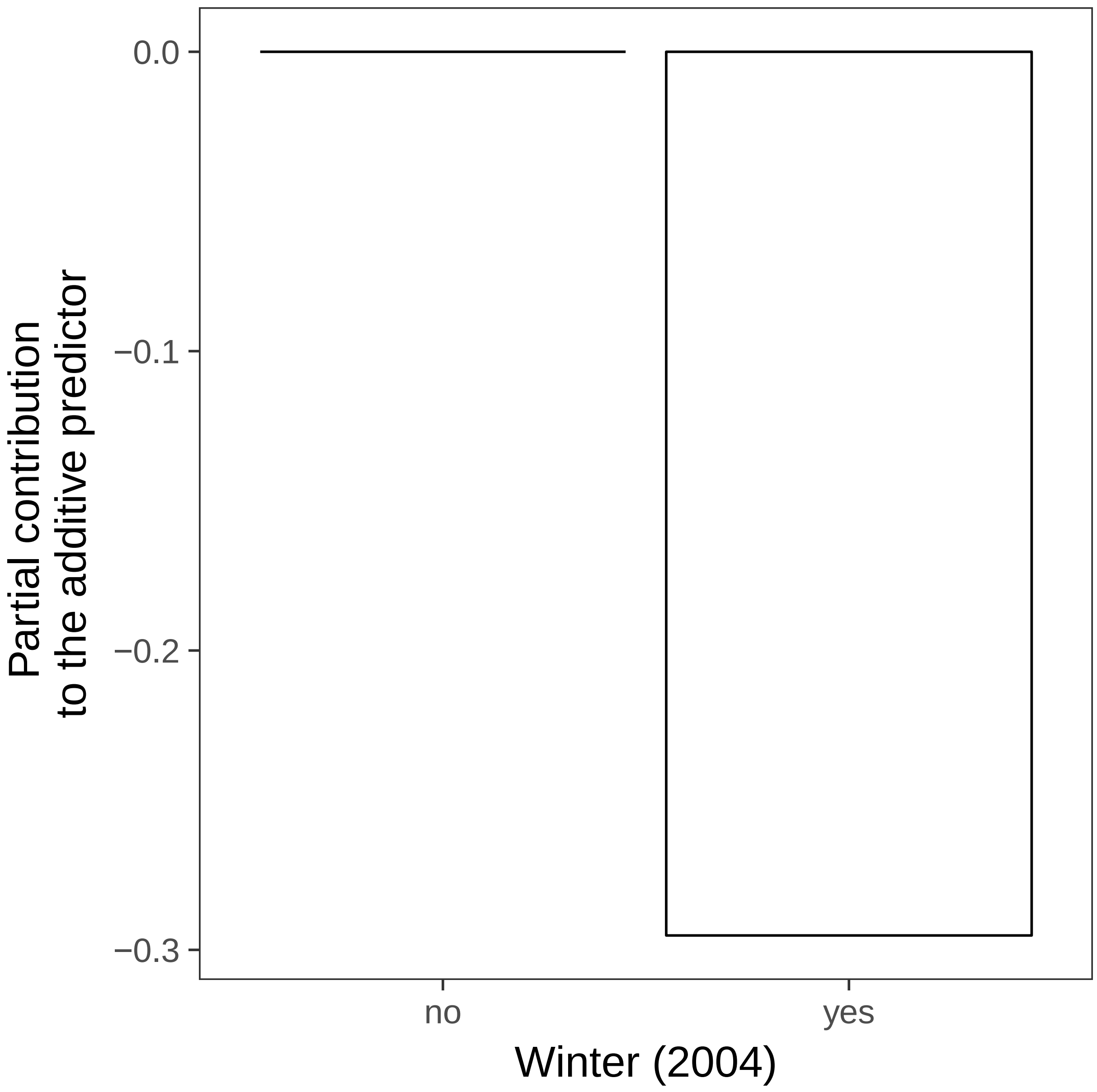

0.0
−0.1
−0.2
−0.3
Partial contribution
to the additive predictor
no
yes
Winter (2004)

## Black, Surf, and White-winged Scoter

### Occupancy

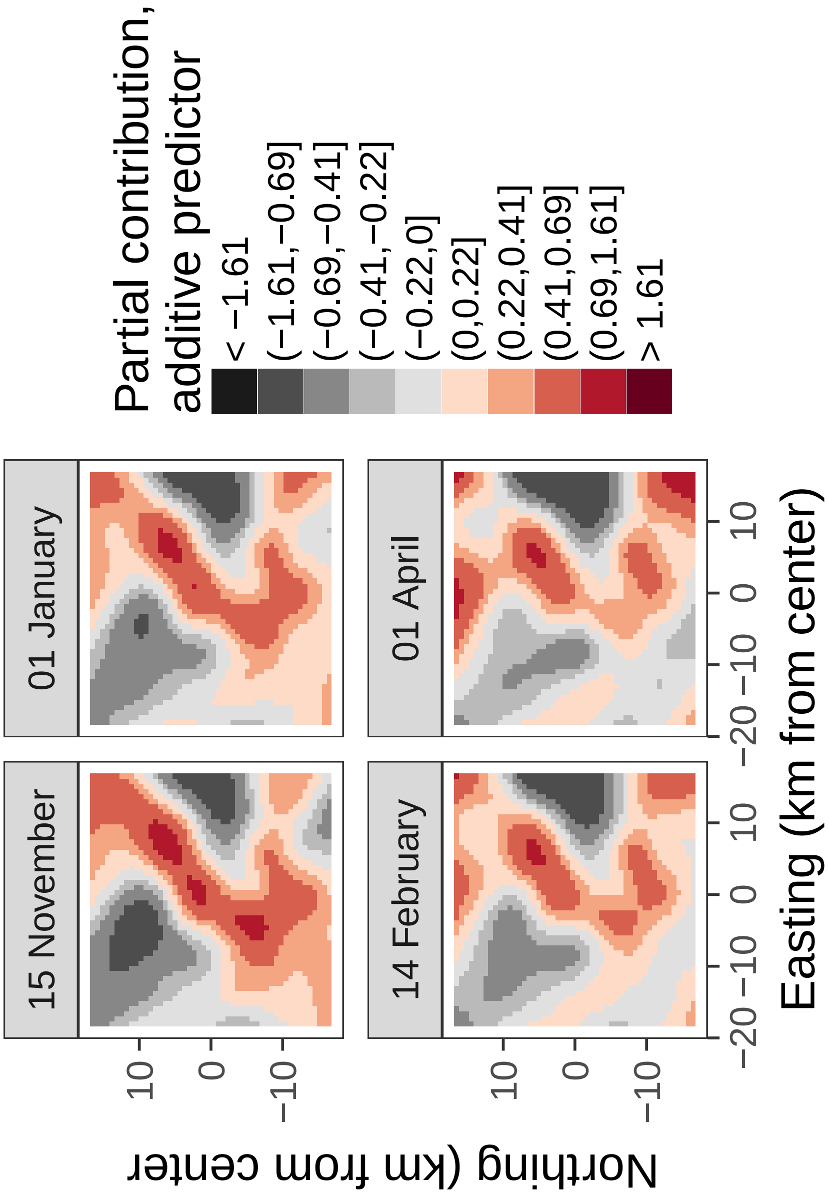

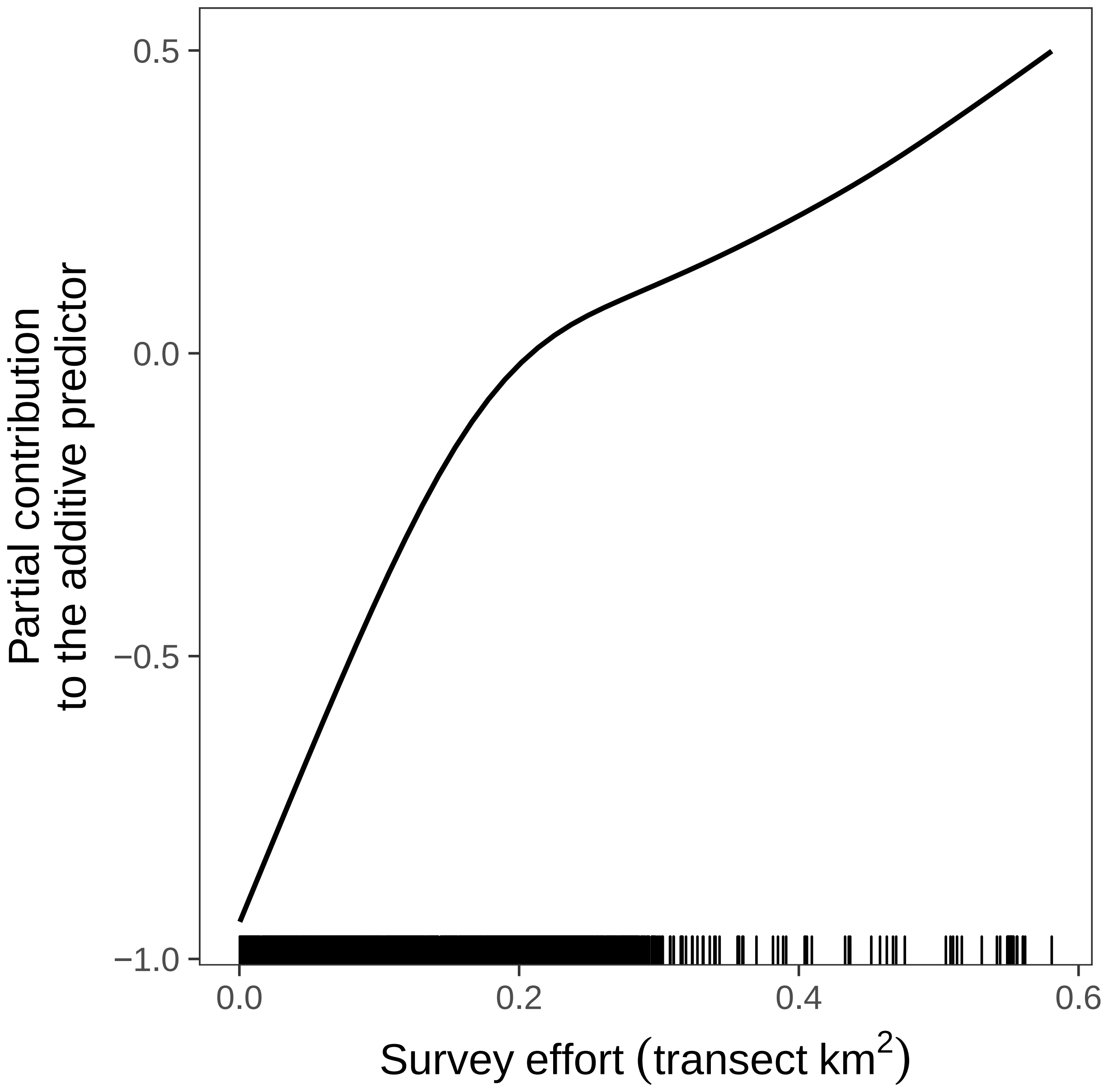
Partial contribution
to the additive predictor
0.5
0.0
−0.5
−1.0
0.0
0.2
0.4
0.6
Survey effort (transect km2)

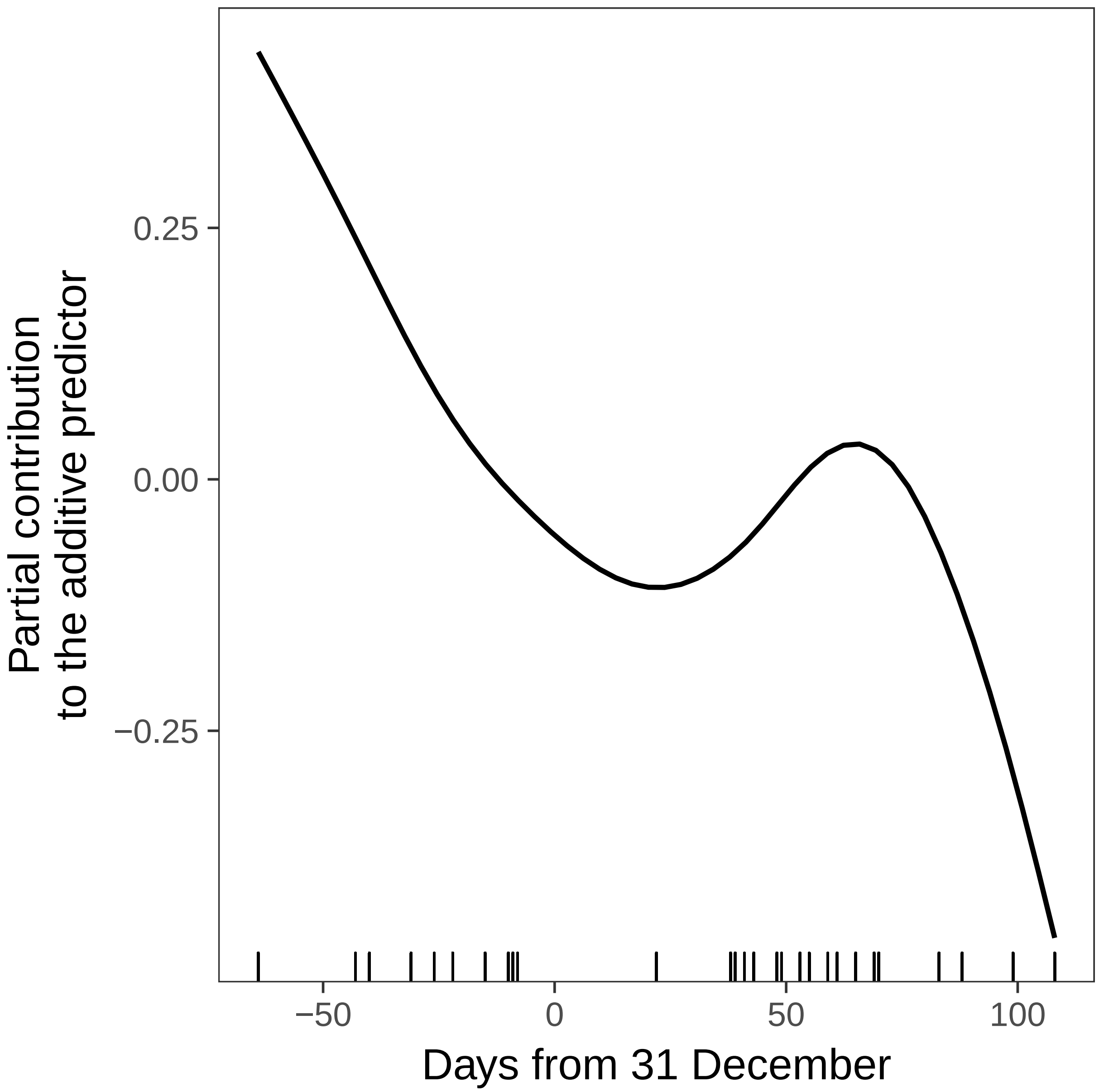

0.25
0.00
−0.25
Partial contribution
to the additive predictor
−50
0
50
100
Days from 31 December

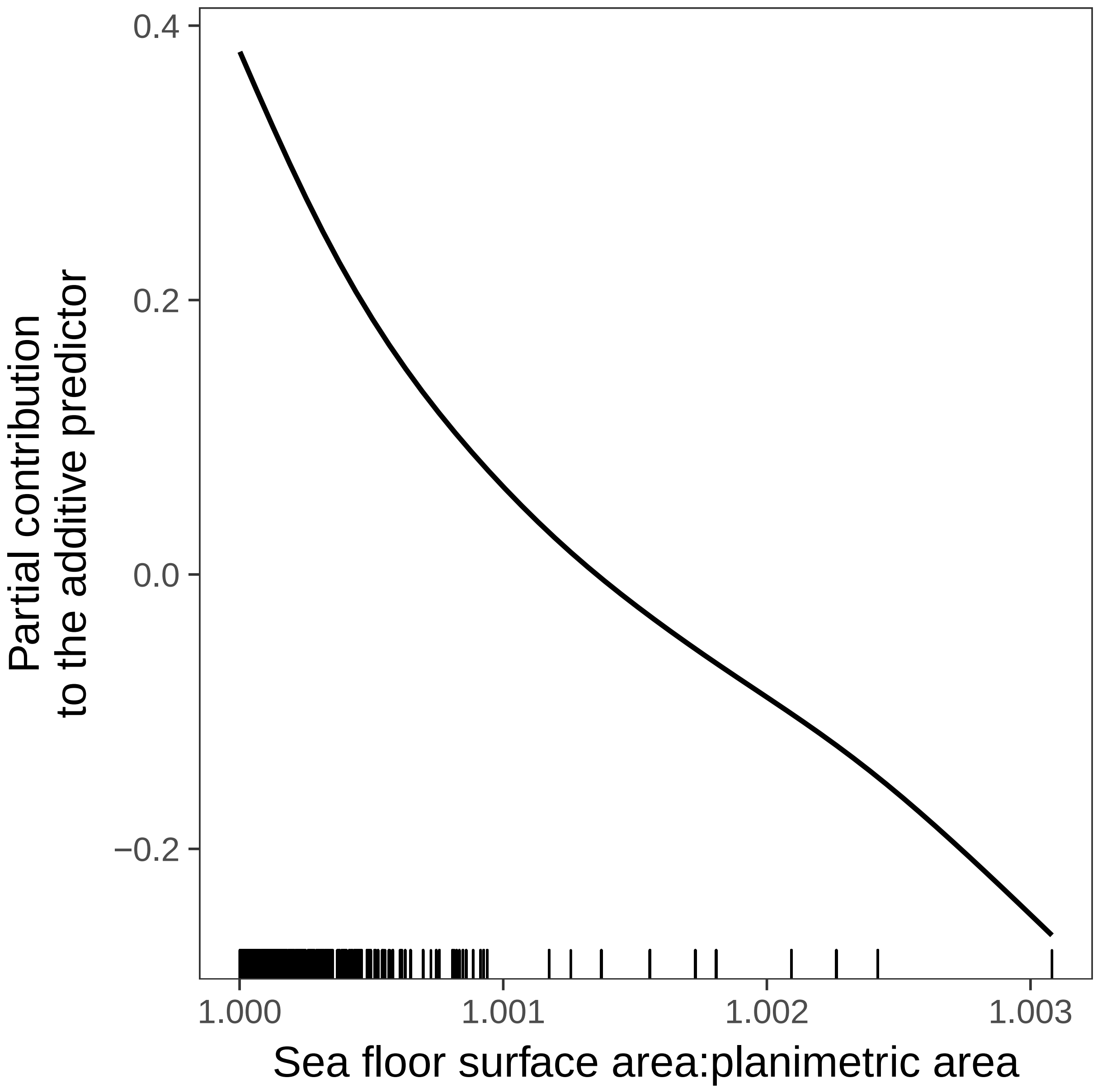

Partial contribution
to the additive predictor
0.4
0.2
0.0
−0.2
1.000
1.001
1.002
1.003
Sea floor surface area:planimetric area

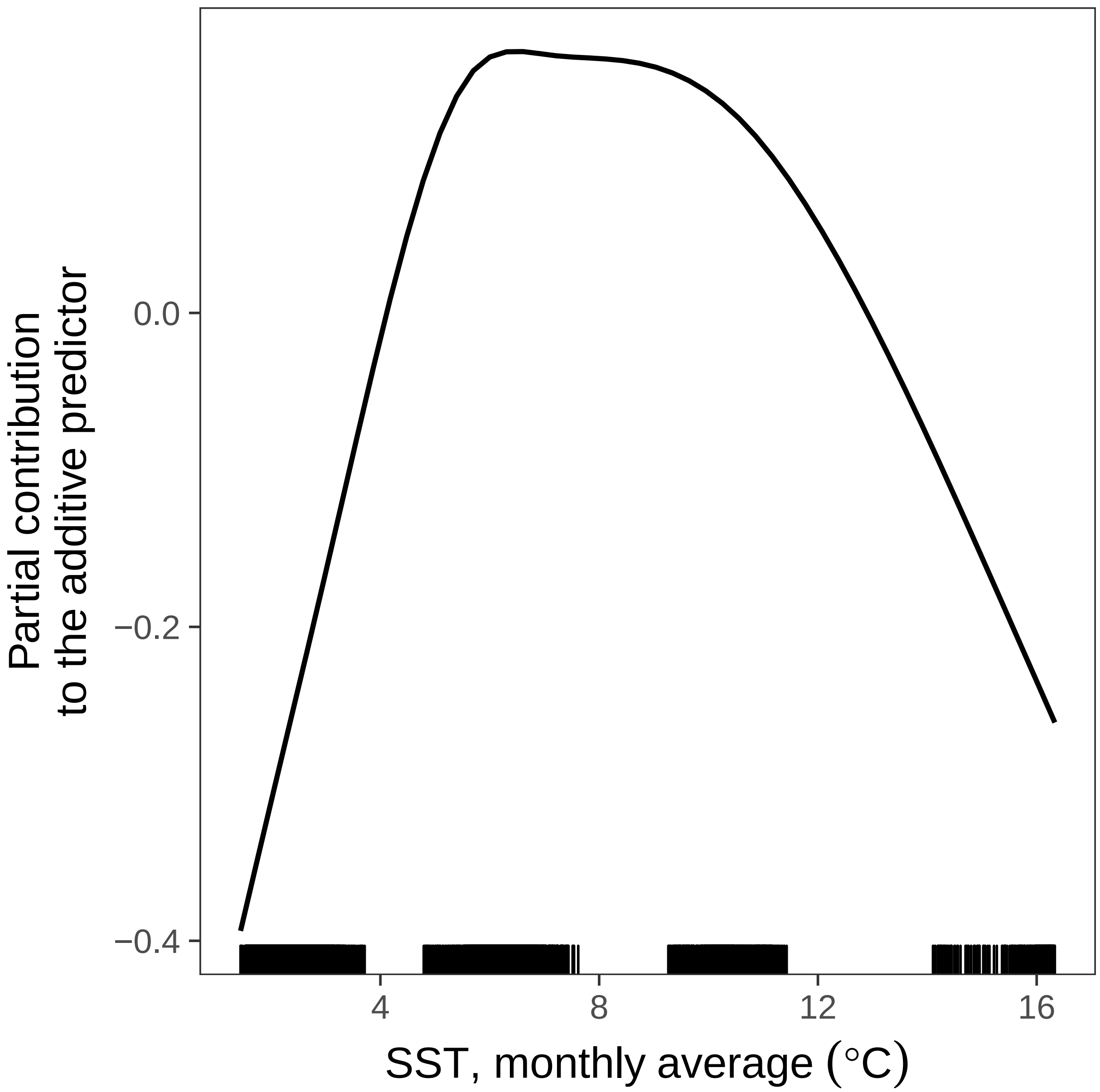

Partial contribution
to the additive predictor
0.0
−0.2
−0.4
4
8
12
16
SST, monthly average (°C)

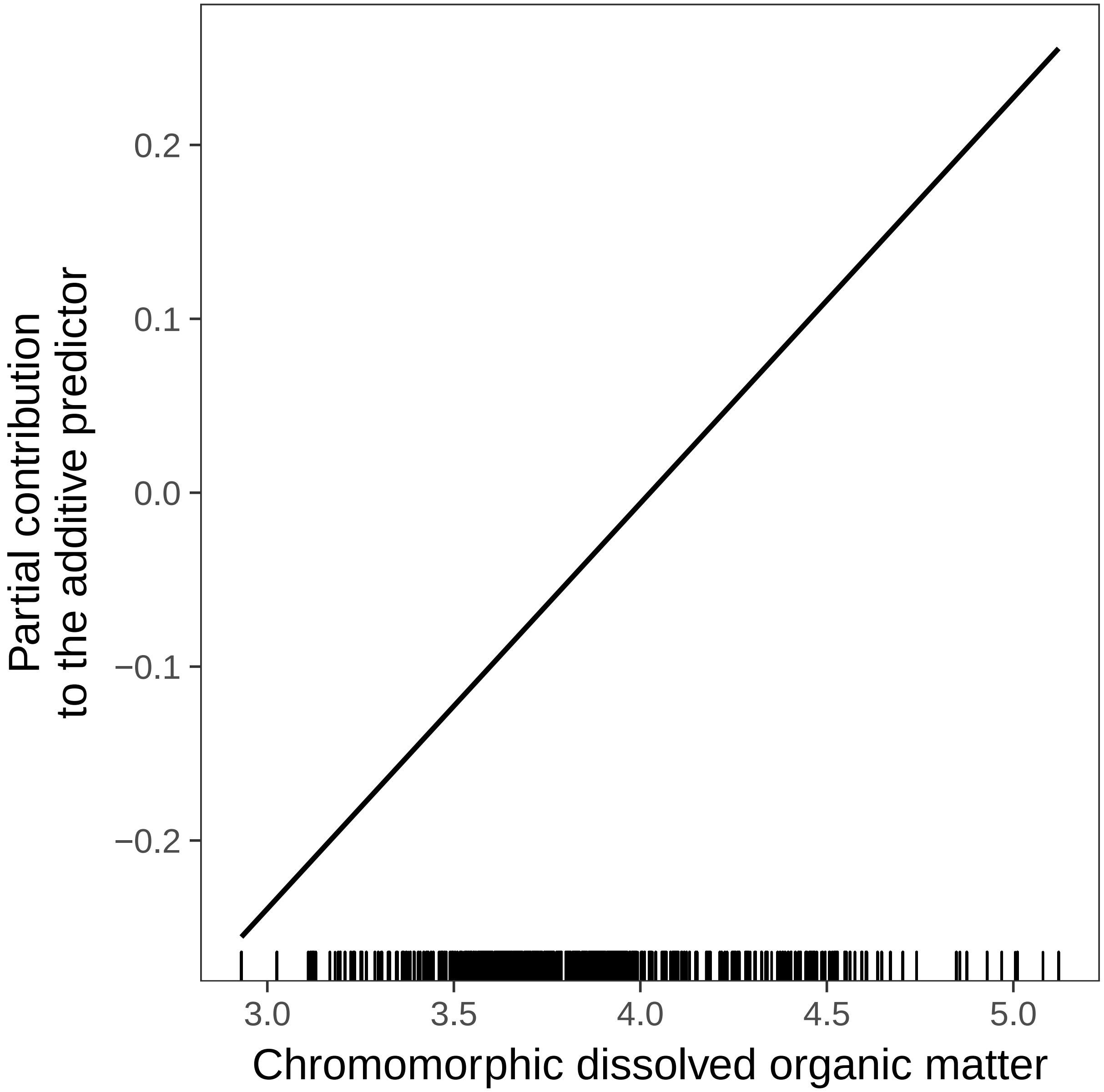

Partial contribution
to the additive predictor
0.2
0.1
0.0
−0.1
−0.2
3.0
3.5
4.0
4.5
5.0
Chromomorphic dissolved organic matter

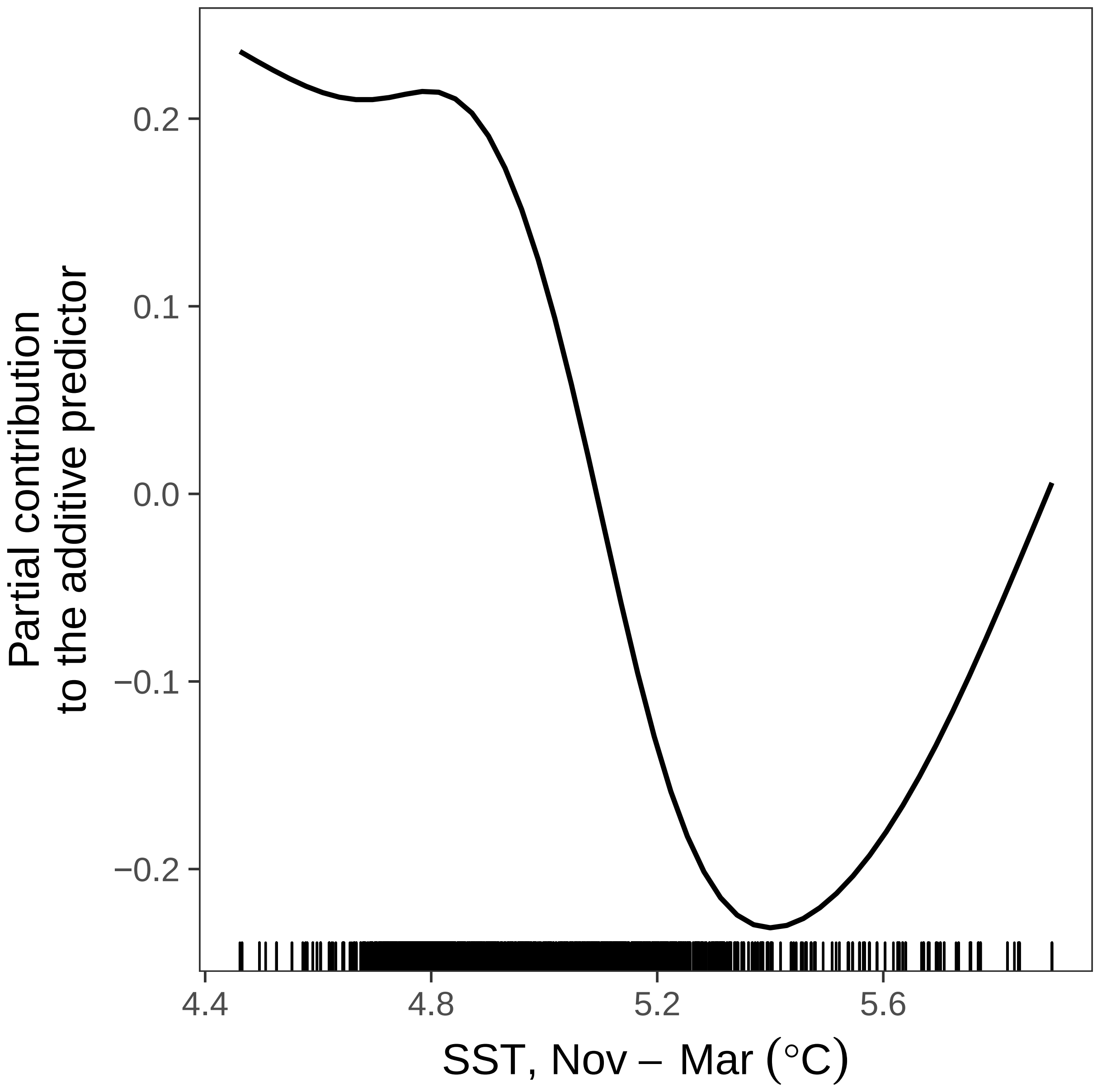
Partial contribution
to the additive predictor
0.2
0.1
0.0
−0.1
−0.2
4.4
4.8
5.2
5.6
SST, Nov – Mar (°C)

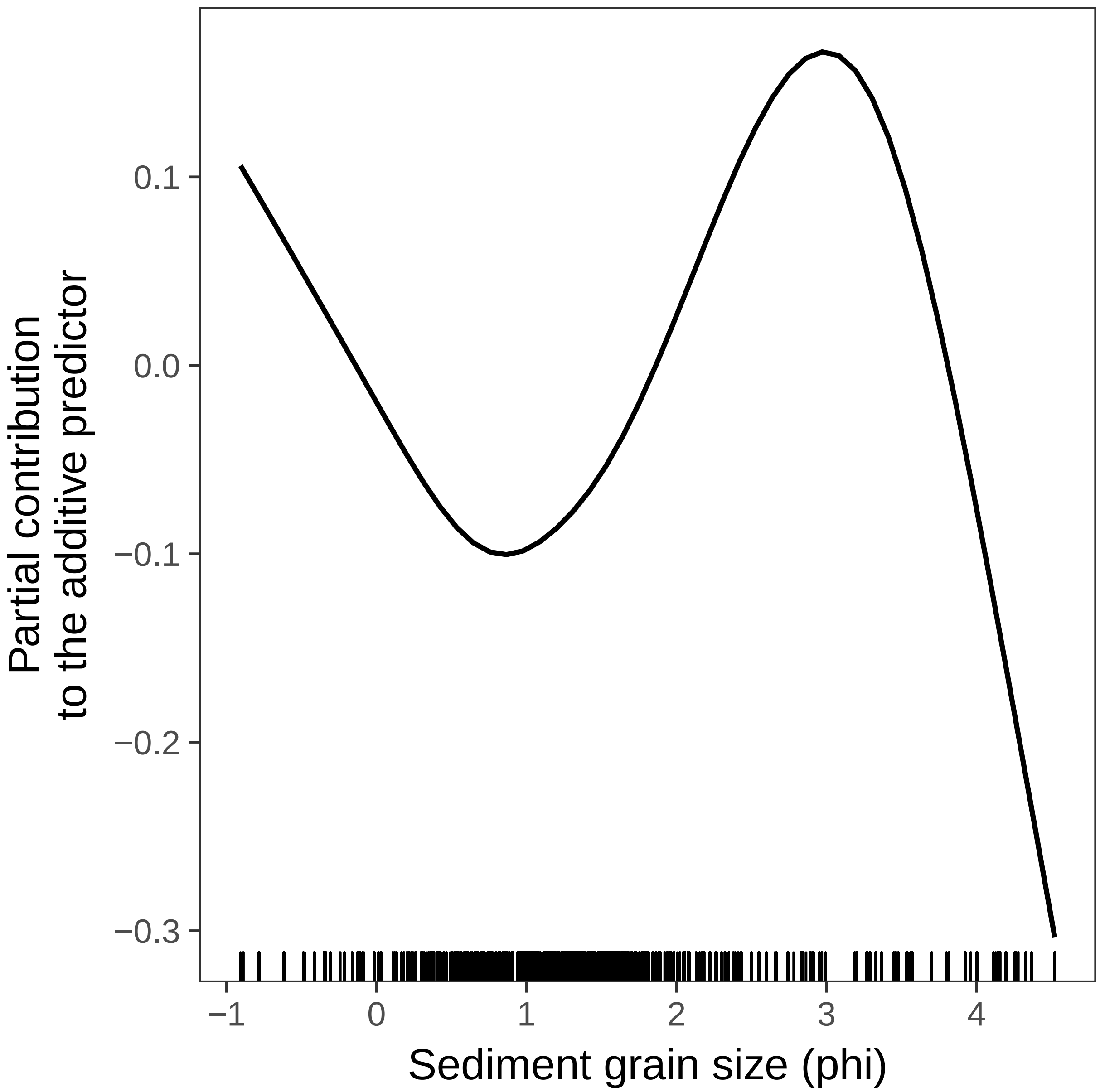

Partial contribution
to the additive predictor
0.1
0.0
−0.1
−0.2
−0.3
−1
0
1
2
3
4
Sediment grain size (phi)

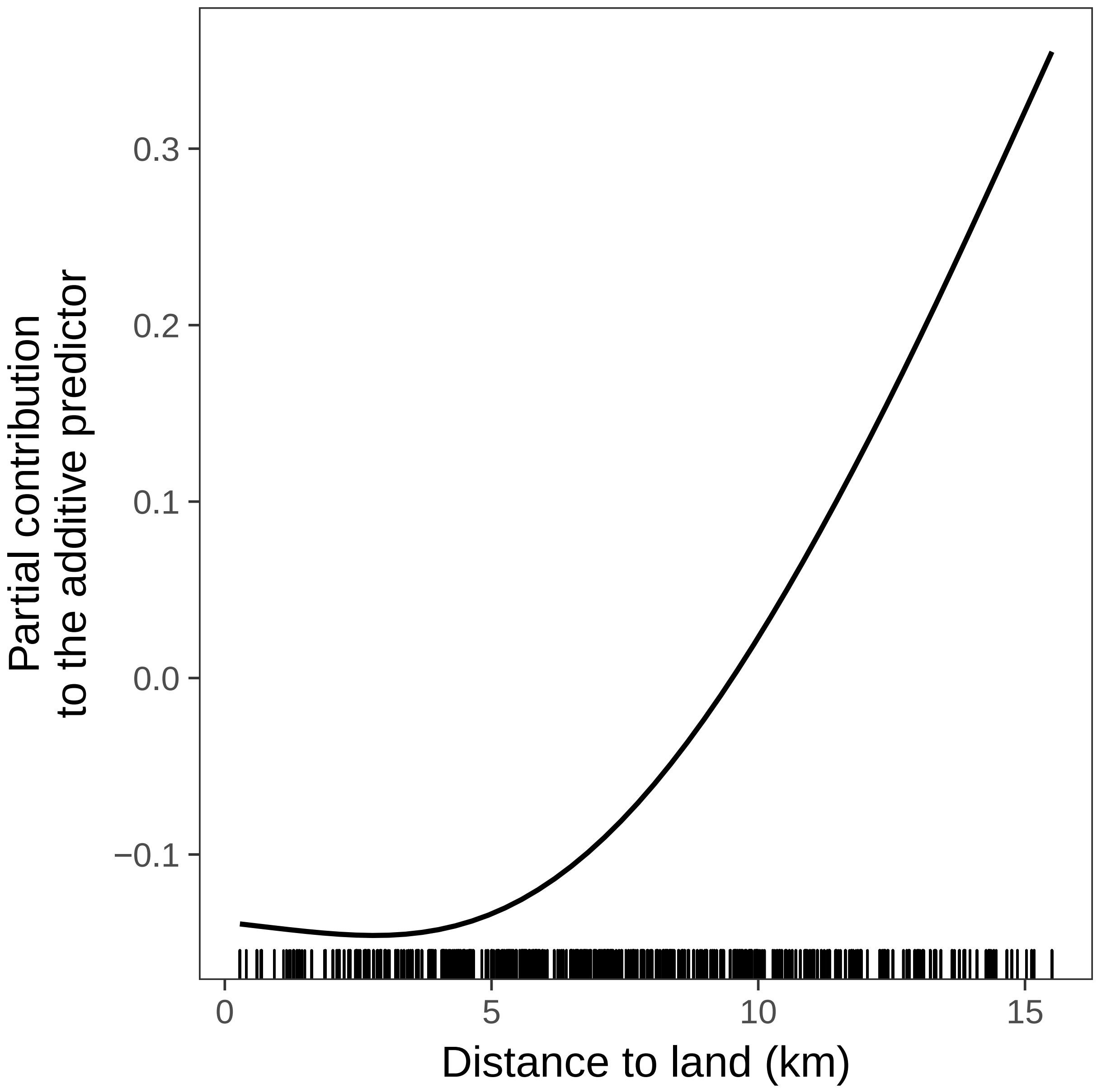
Partial contribution
to the additive predictor
0.3
0.2
0.1
0.0
−0.1
0
5
10
15
Distance to land (km)

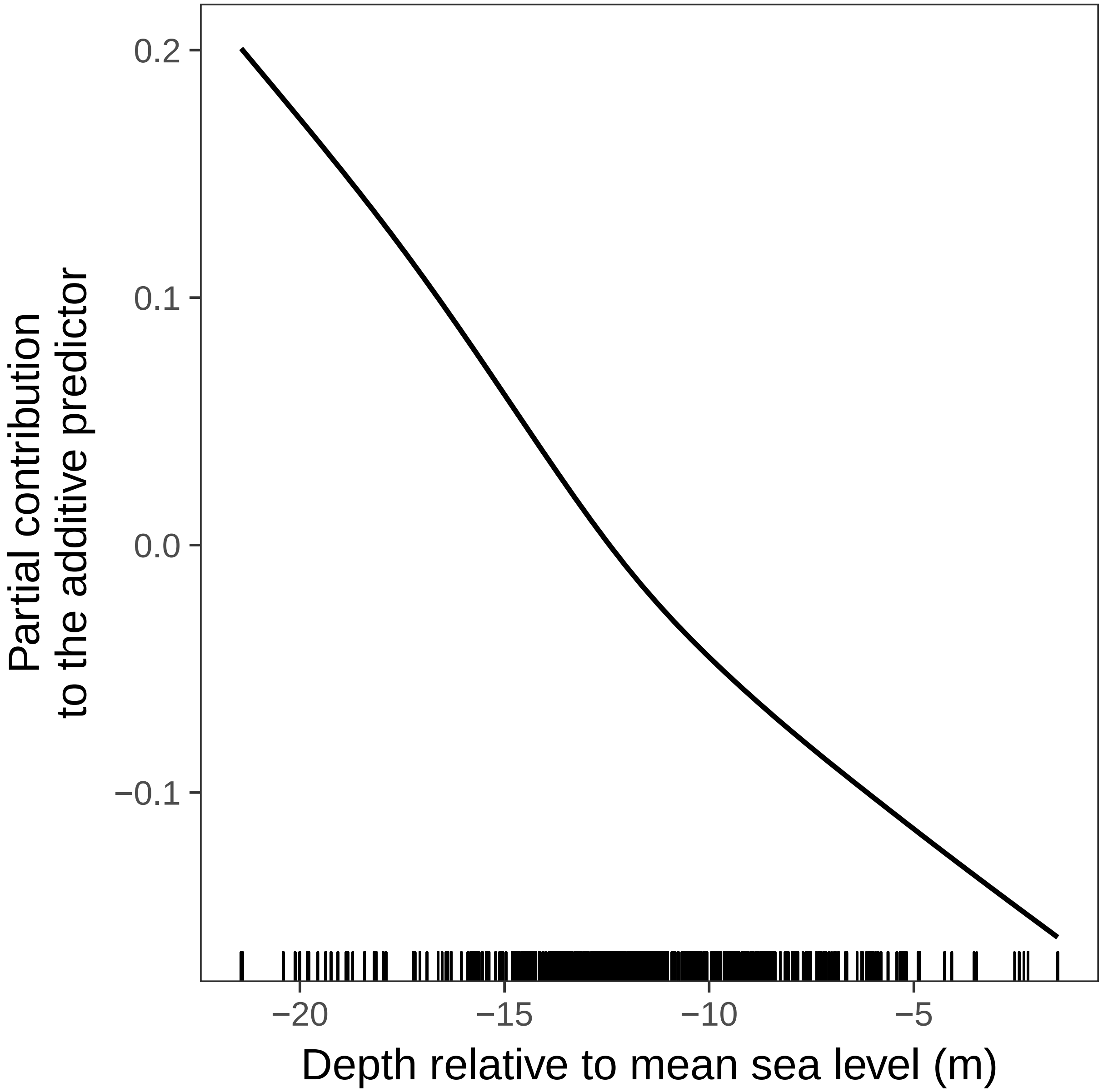
0.2
0.1
0.0
−0.1
Partial contribution
to the additive predictor
−20
−15
−10
−5
Depth relative to mean sea level (m)

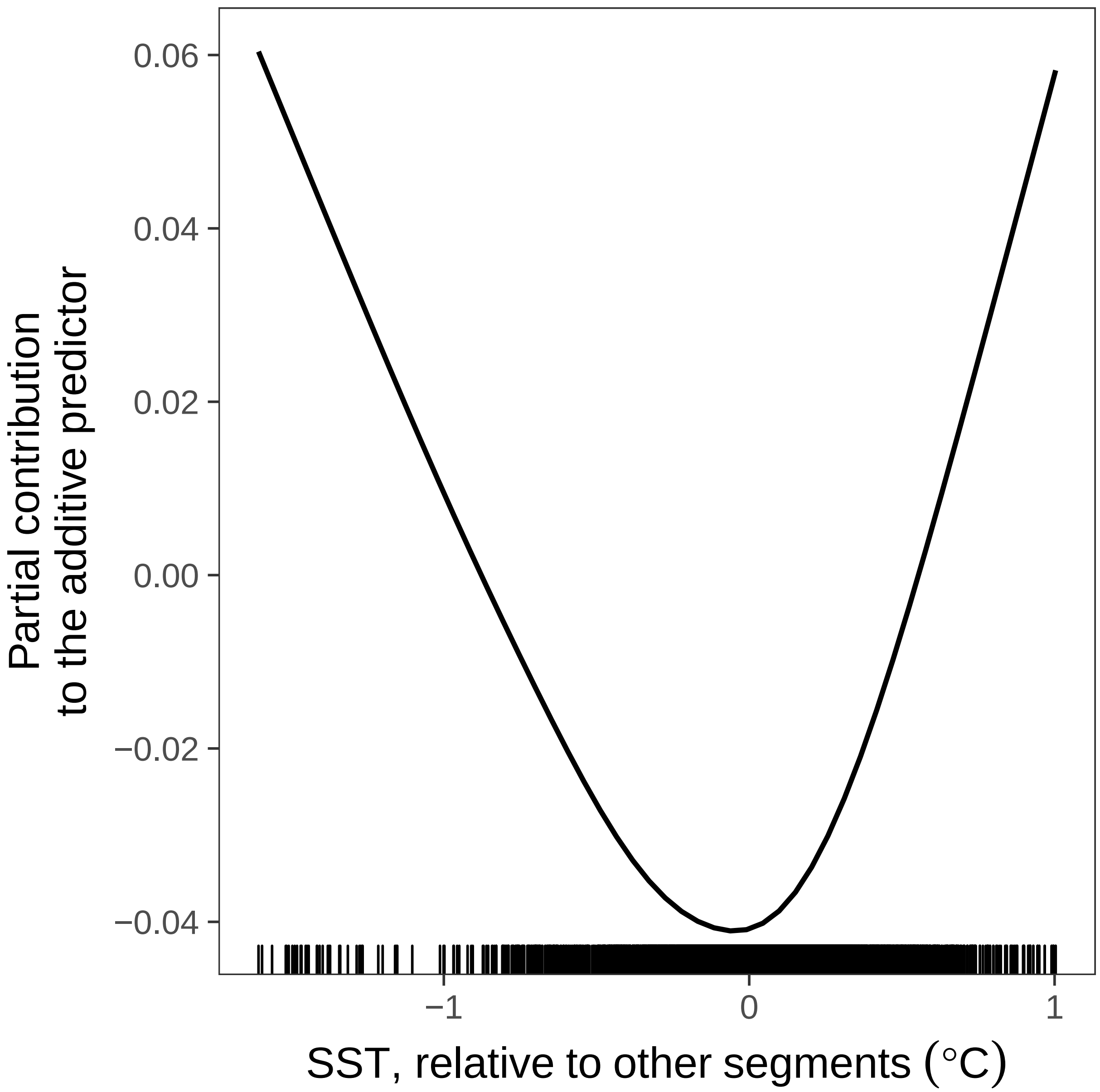
0.06
0.04
0.02
0.00
−0.02
−0.04
Partial contribution
to the additive predictor
−1
0
1
SST, relative to other segments (°C)

**Conditional mean**

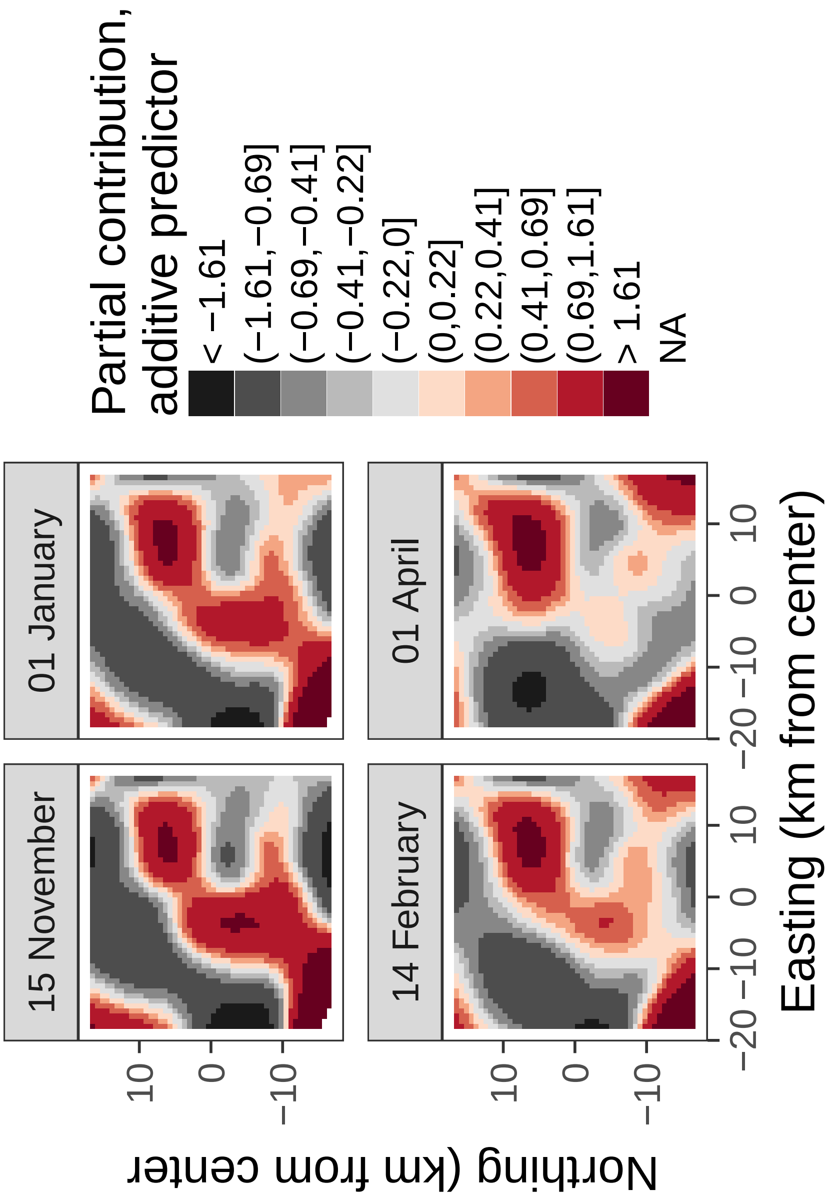

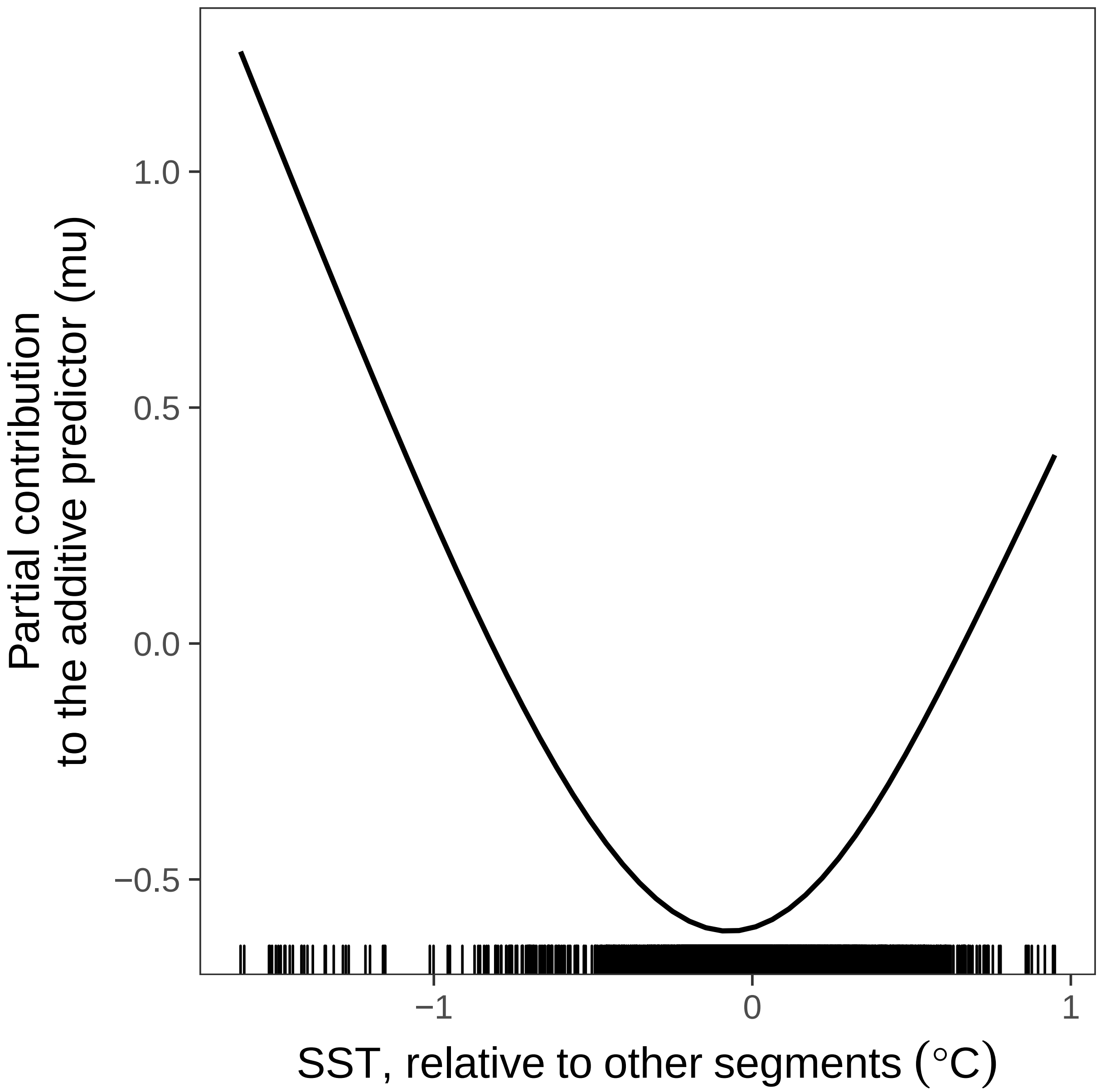
Partial contribution
to the additive predictor (mu)
1.0
0.5
0.0
−0.5
−1
0
1
SST, relative to other segments (°C)

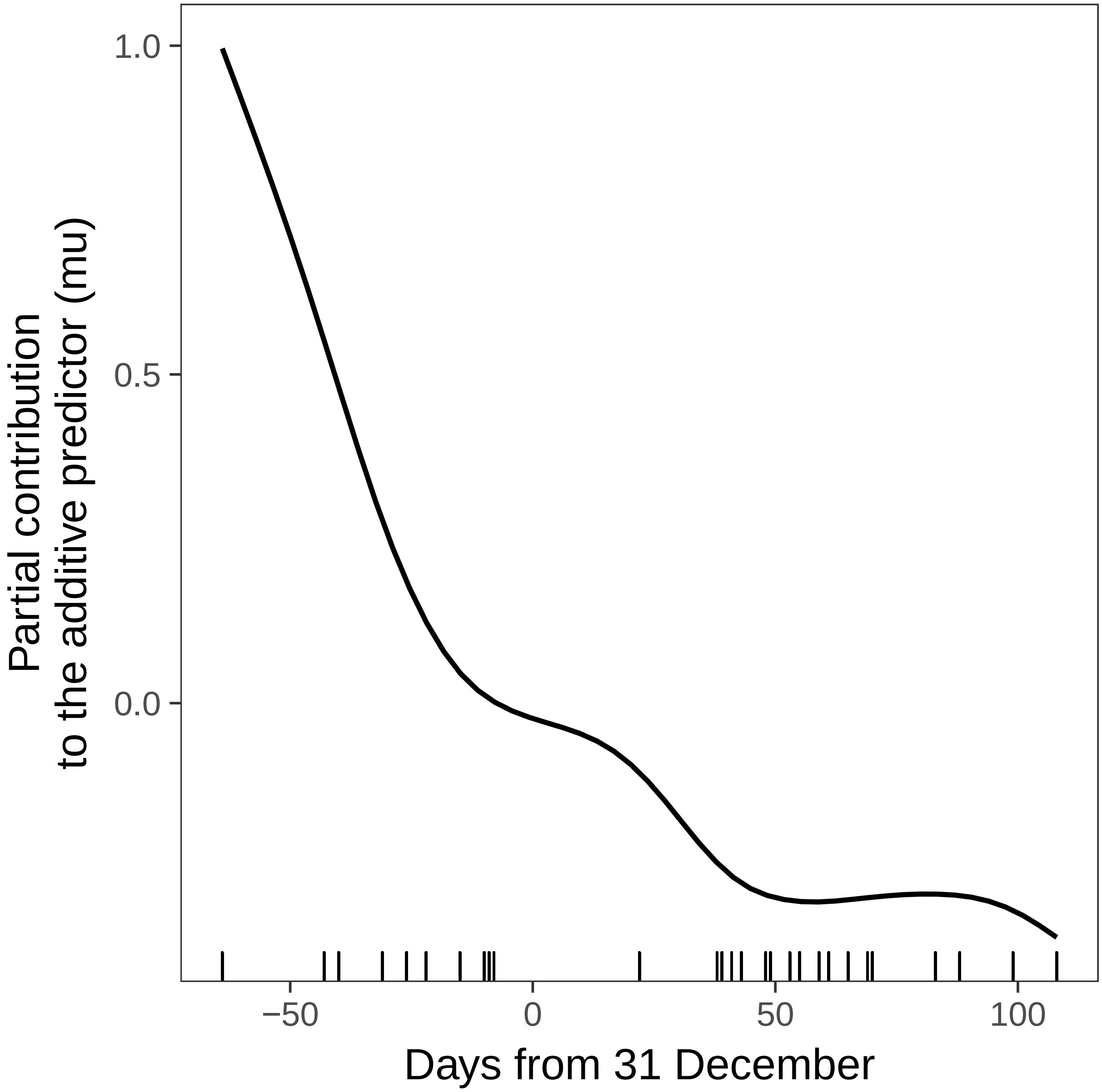

1.0
0.5
0.0
Partial contribution
to the additive predictor (mu)
−50
0
50
100
Days from 31 December

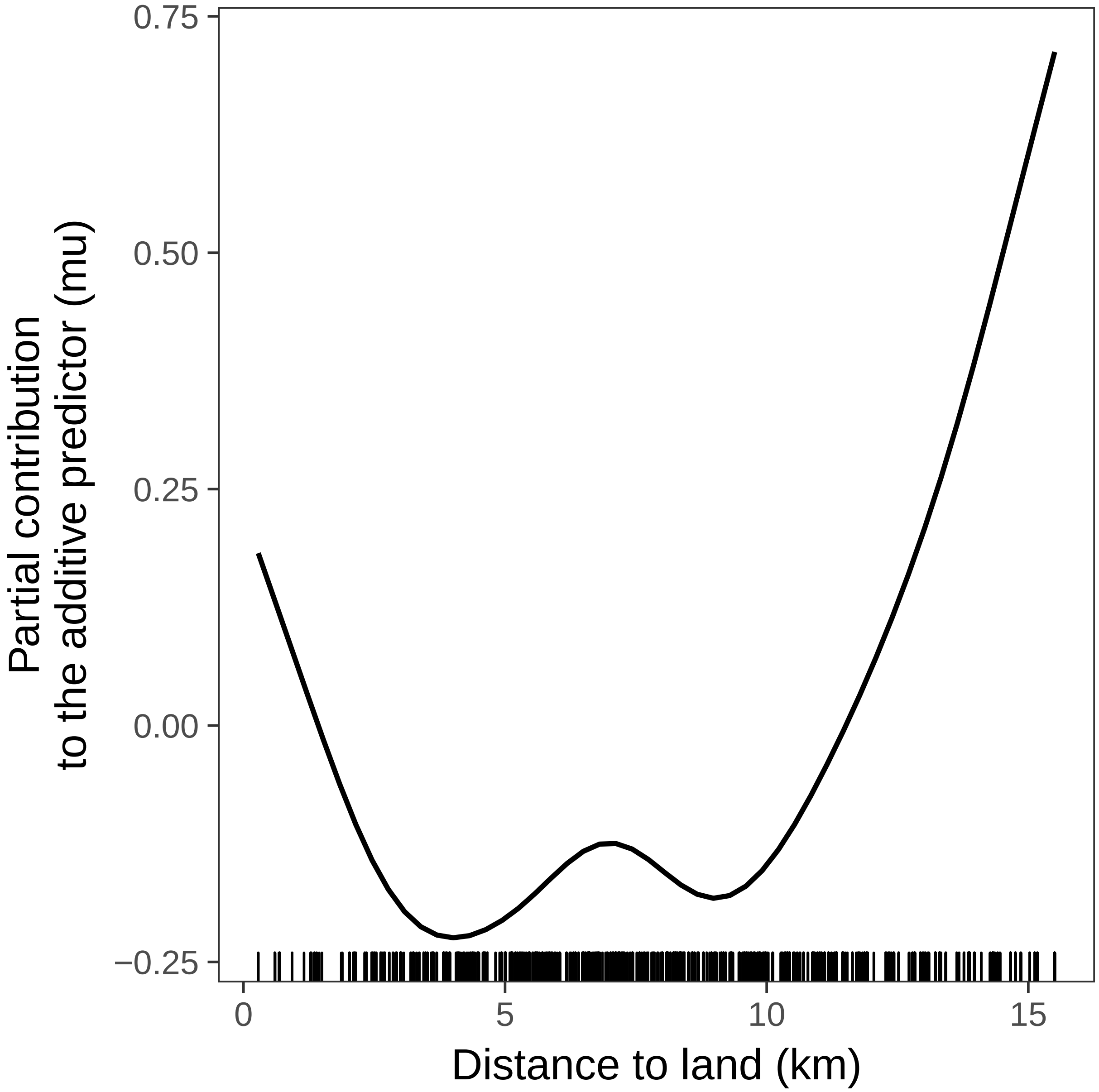

0.75
0.50
0.25
0.00
−0.25
Partial contribution
to the additive predictor (mu)
0
5
10
15
Distance to land (km)

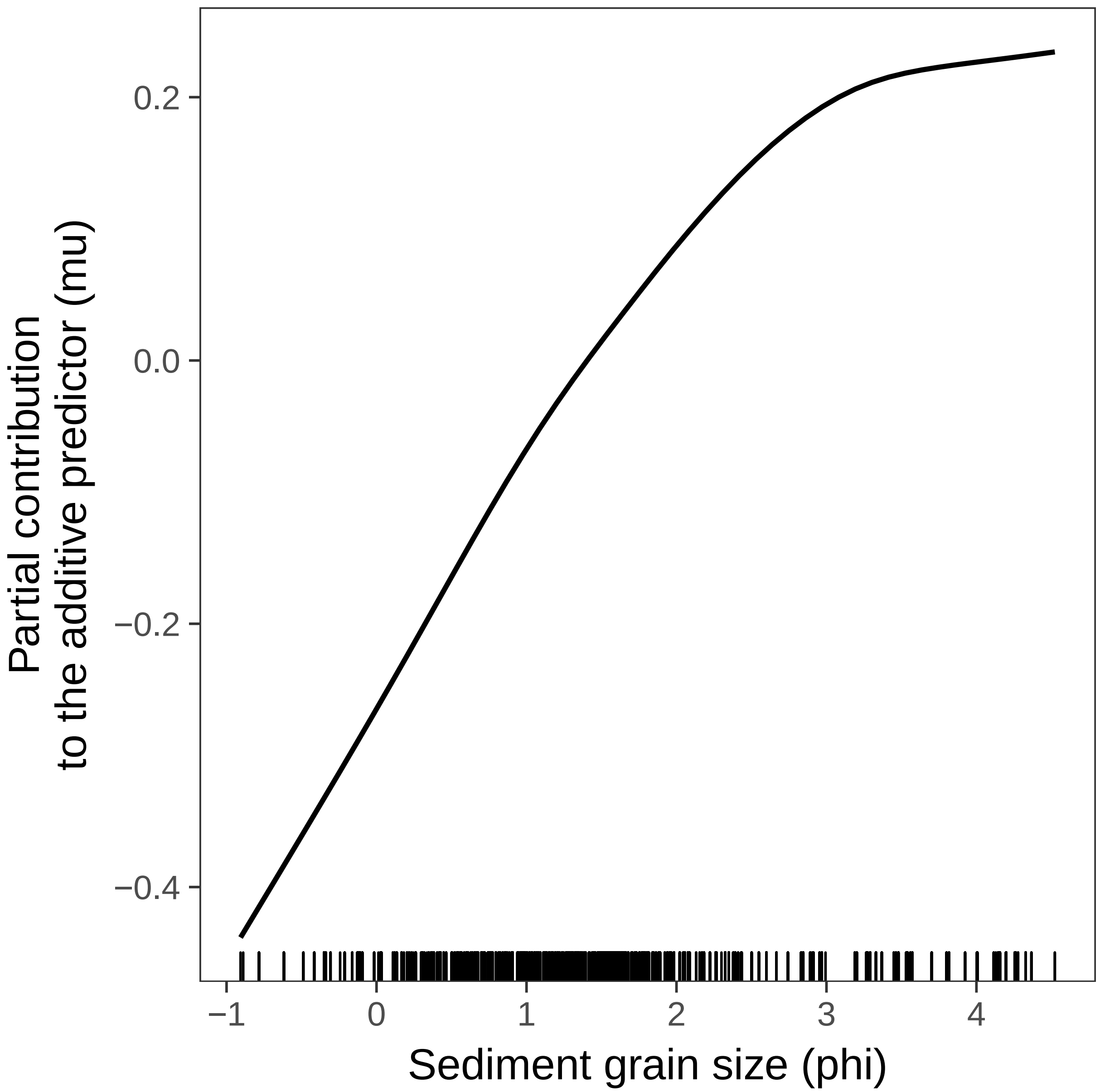

Partial contribution
to the additive predictor (mu)
0.2
0.0
−0.2
−0.4
−1
0
1
2
3
4
Sediment grain size (phi)

**Conditional overdispersion**

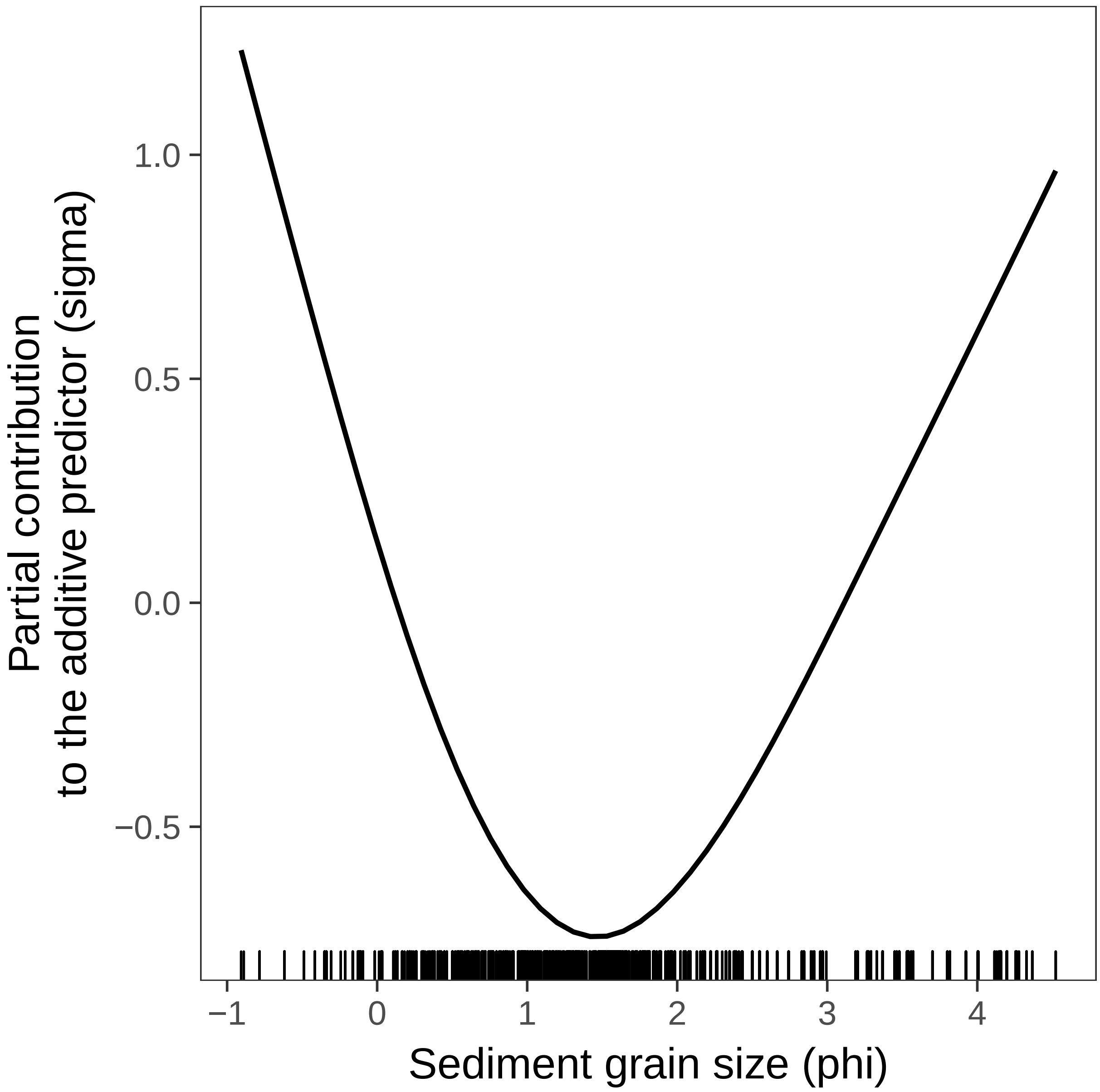

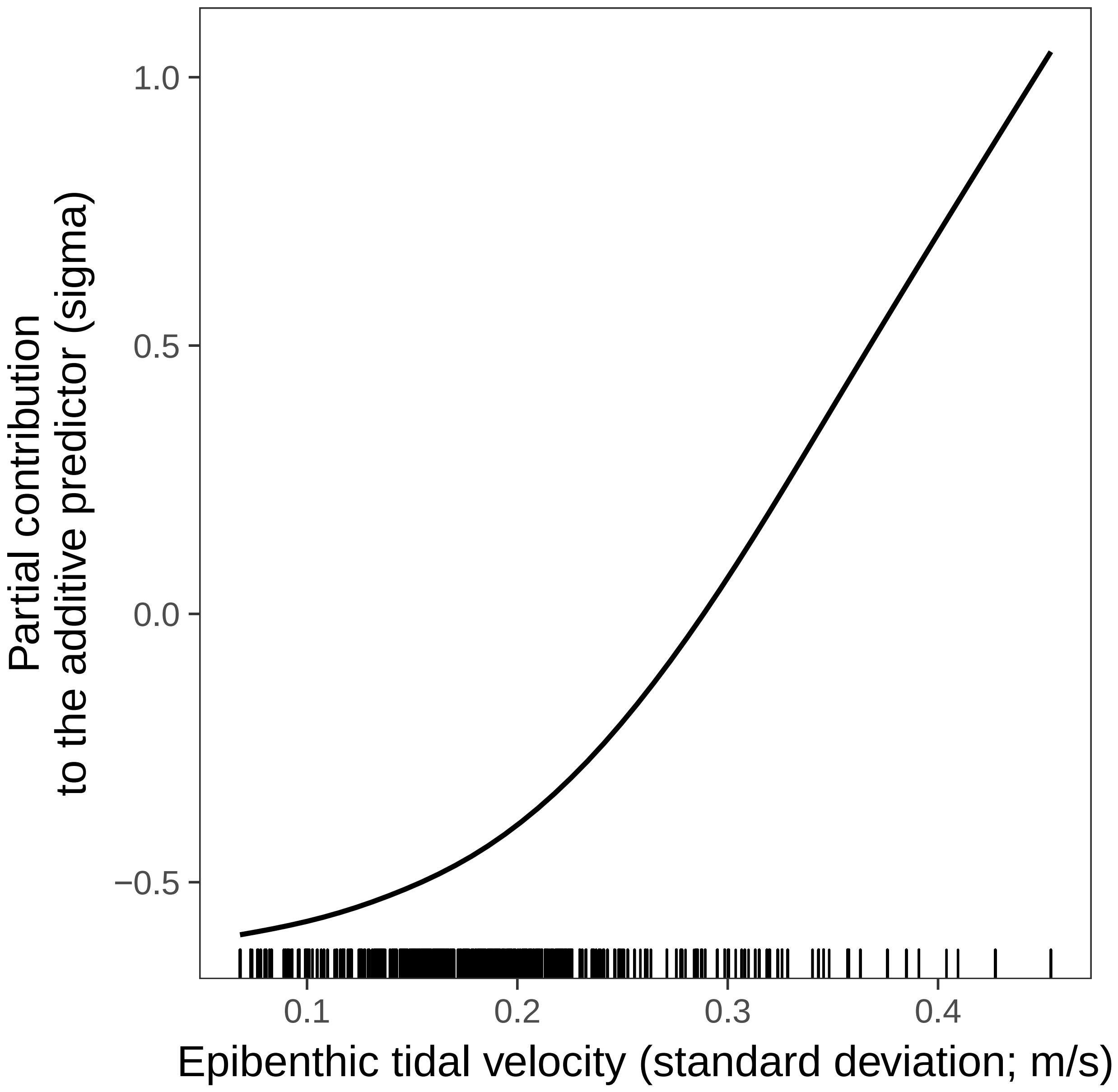

Partial contribution
to the additive predictor (sigma)
1.0
0.5
0.0
−0.5
0.1
0.2
0.3
0.4
Epibenthic tidal velocity (standard deviation; m/s)

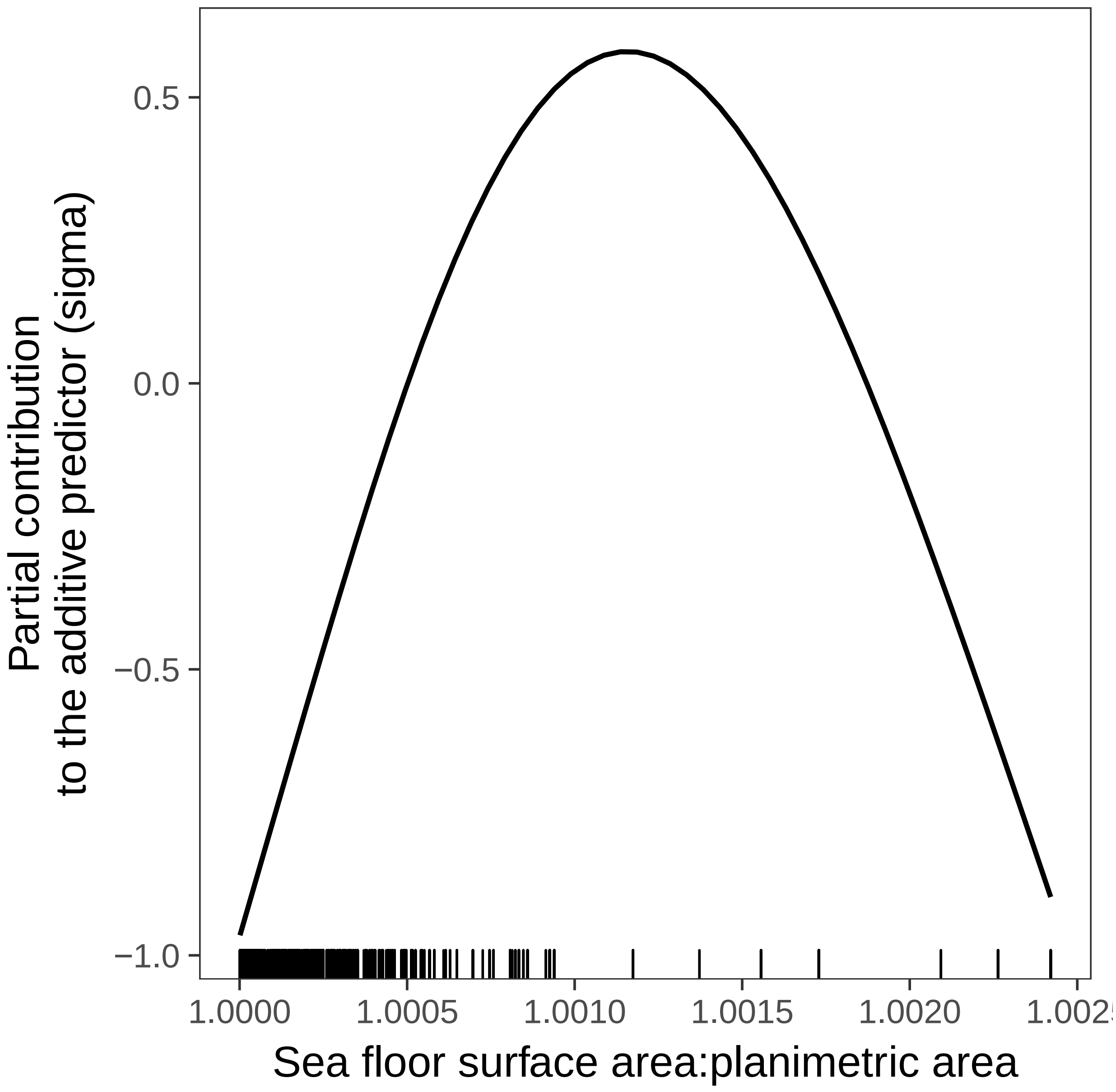

Partial contribution
to the additive predictor (sigma)
0.5
0.0
−0.5
−1.0
1.0000
1.0005
1.0010
1.0015
1.0020
Sea floor surface area:planimetric area

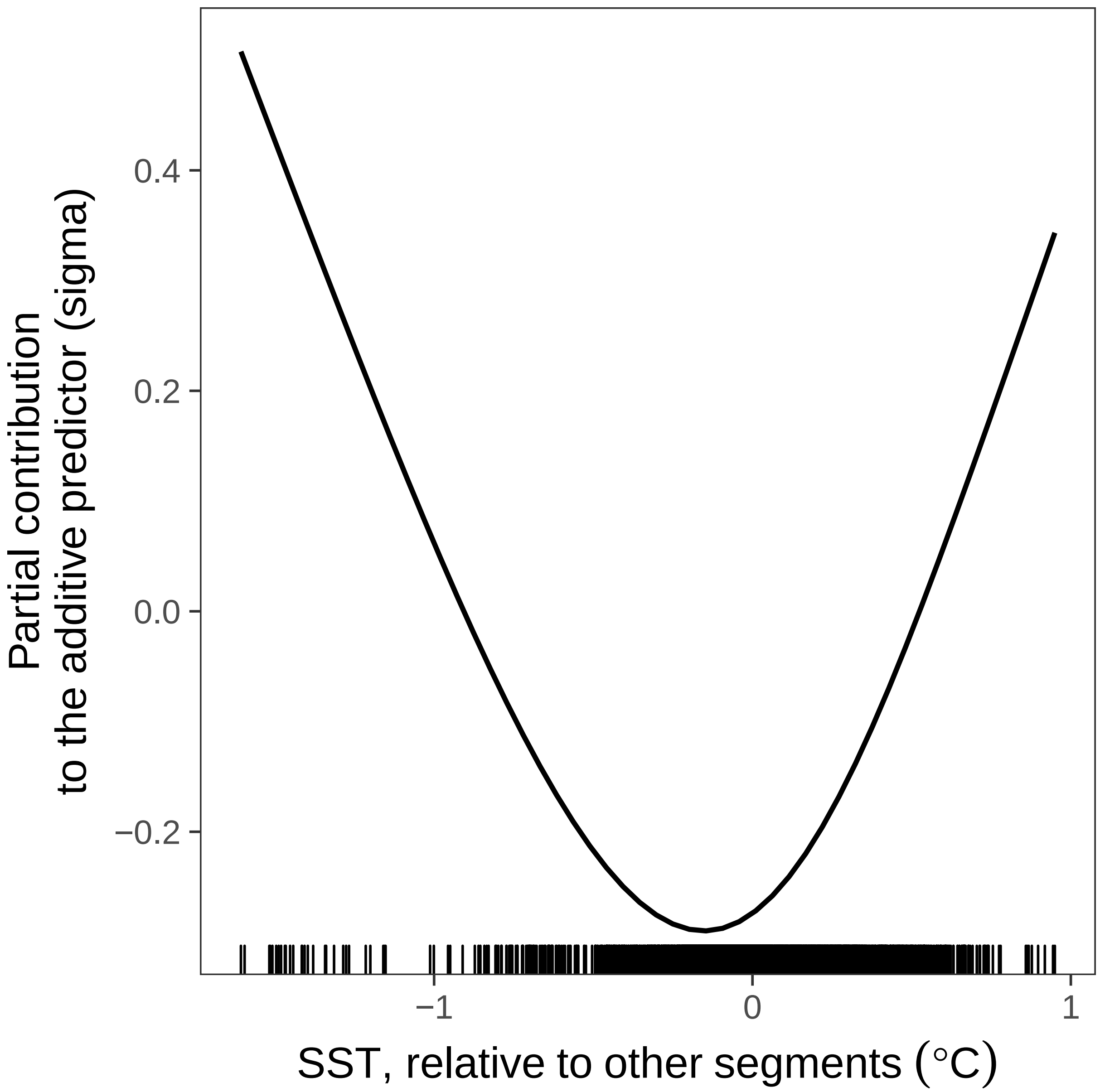

Partial contribution
to the additive predictor (sigma)
0.4
0.2
0.0
−0.2
−1
0
1
SST, relative to other segments (°C)

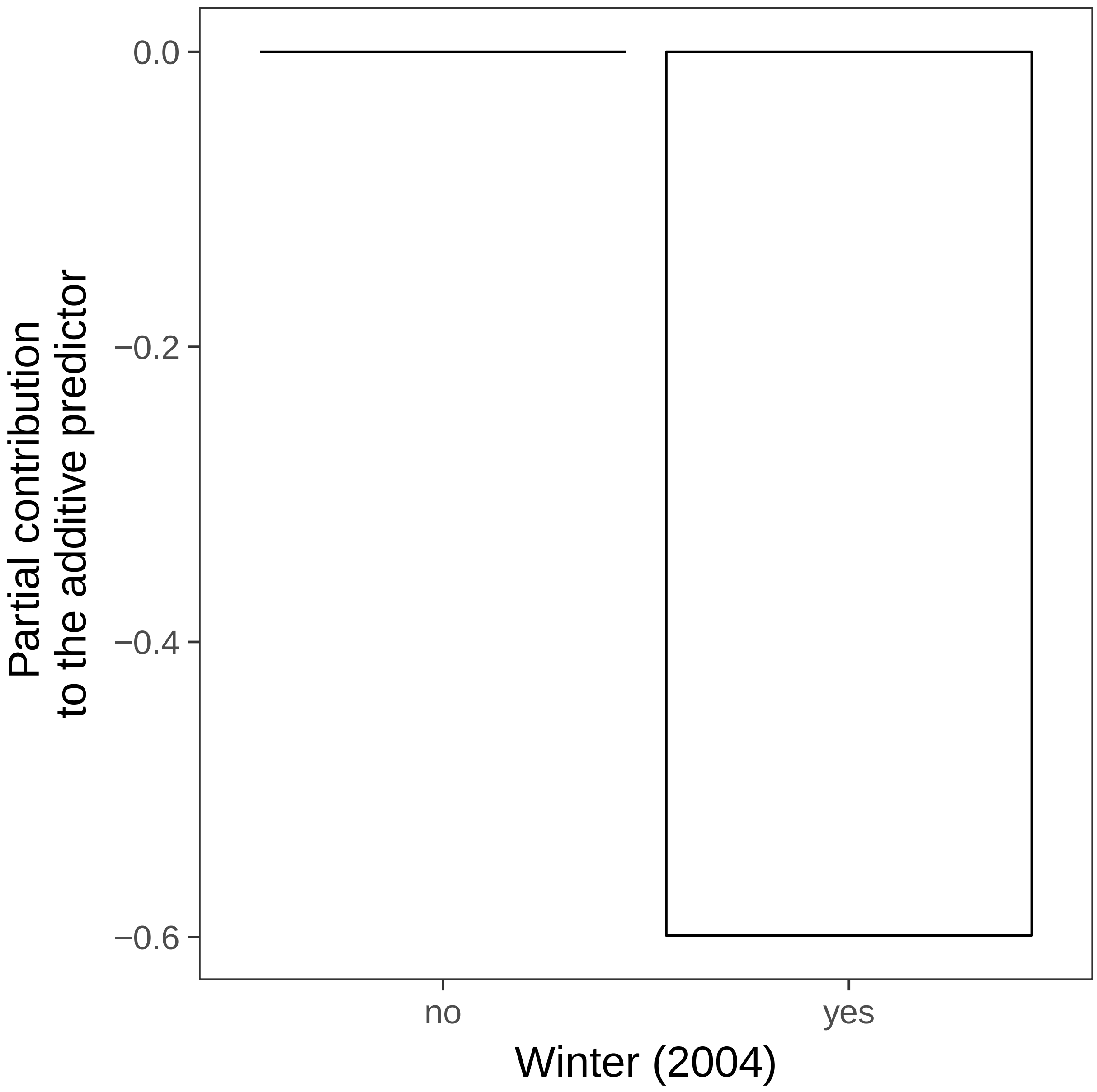
0.0
−0.2
−0.4
−0.6
Partial contribution
to the additive predictor
no
yes
Winter (2004)

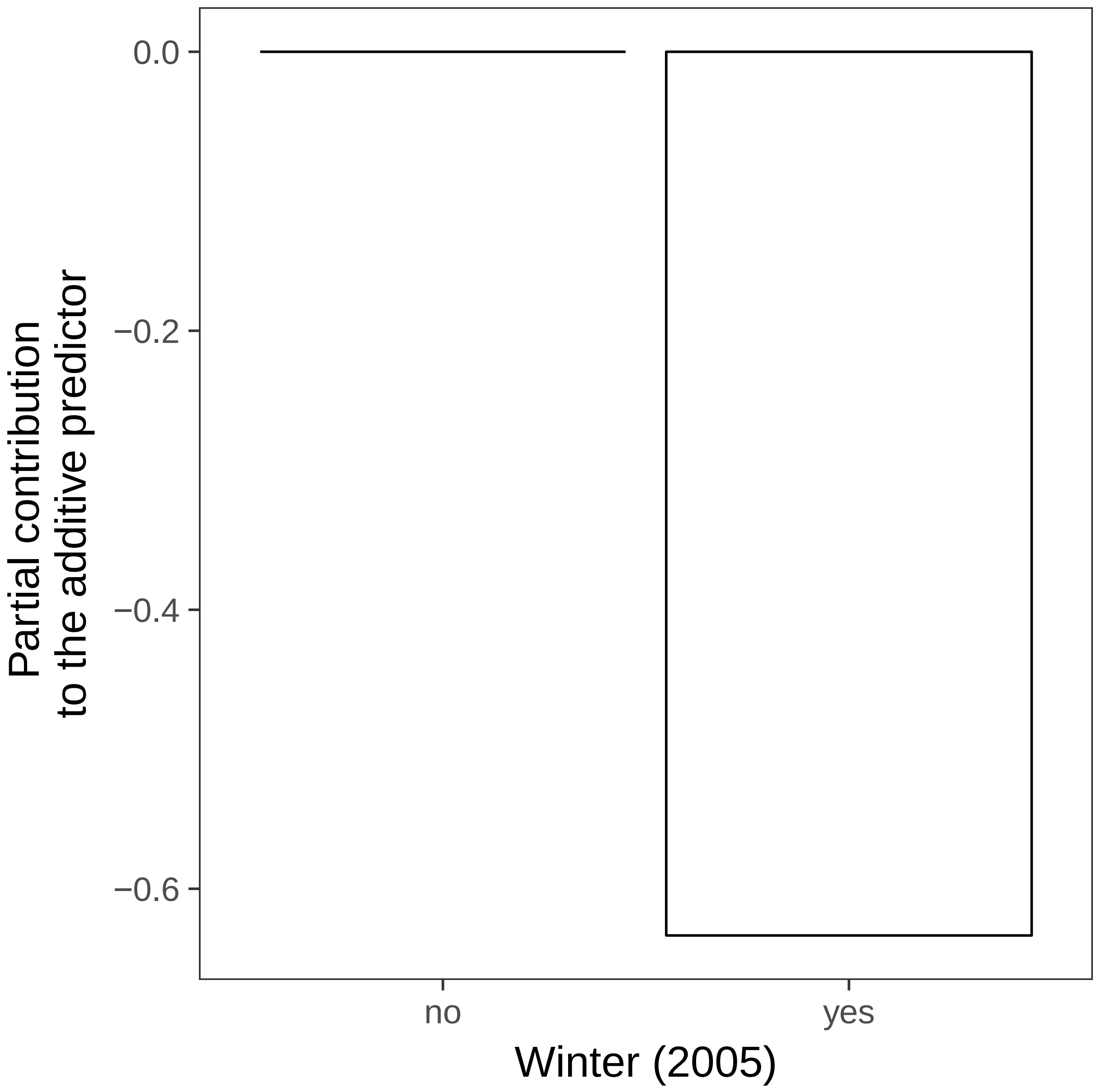

0.0
−0.2
−0.4
−0.6
Partial contribution
to the additive predictor
no
yes
Winter (2005)

## Long-tailed Duck

### Occupancy

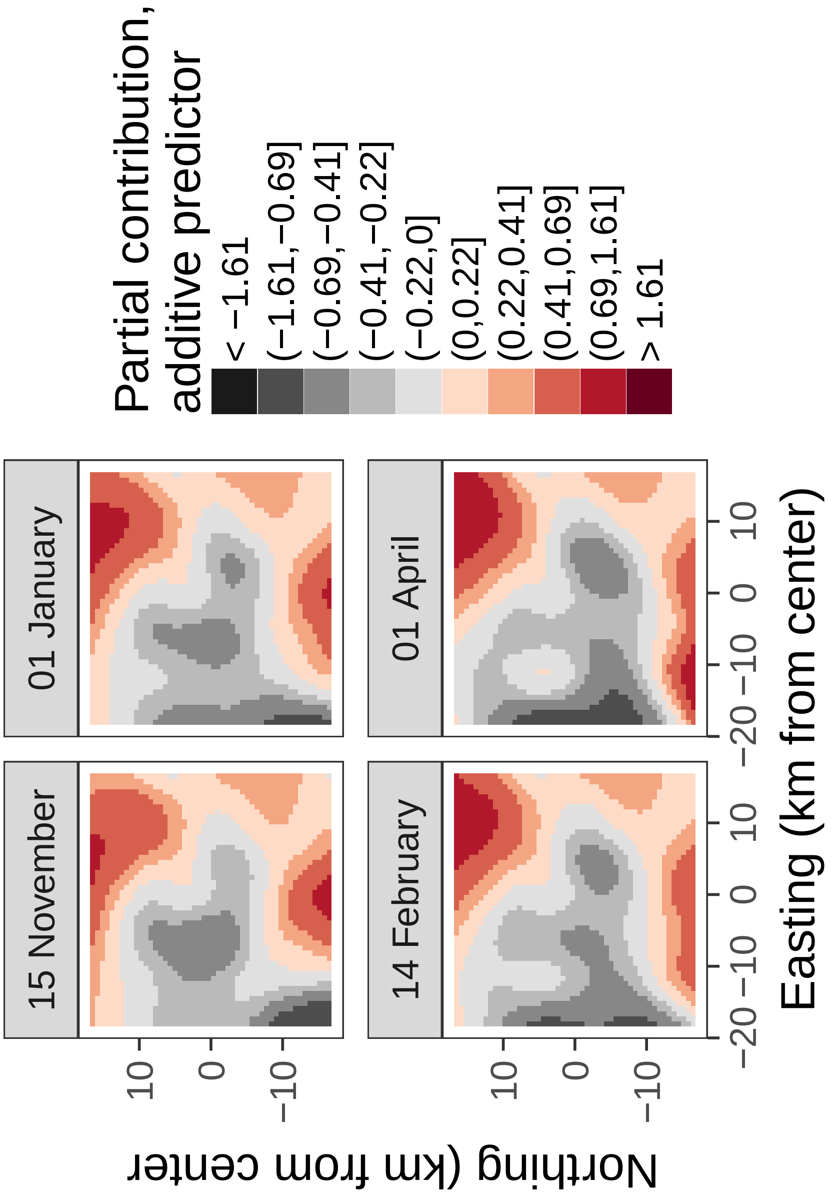

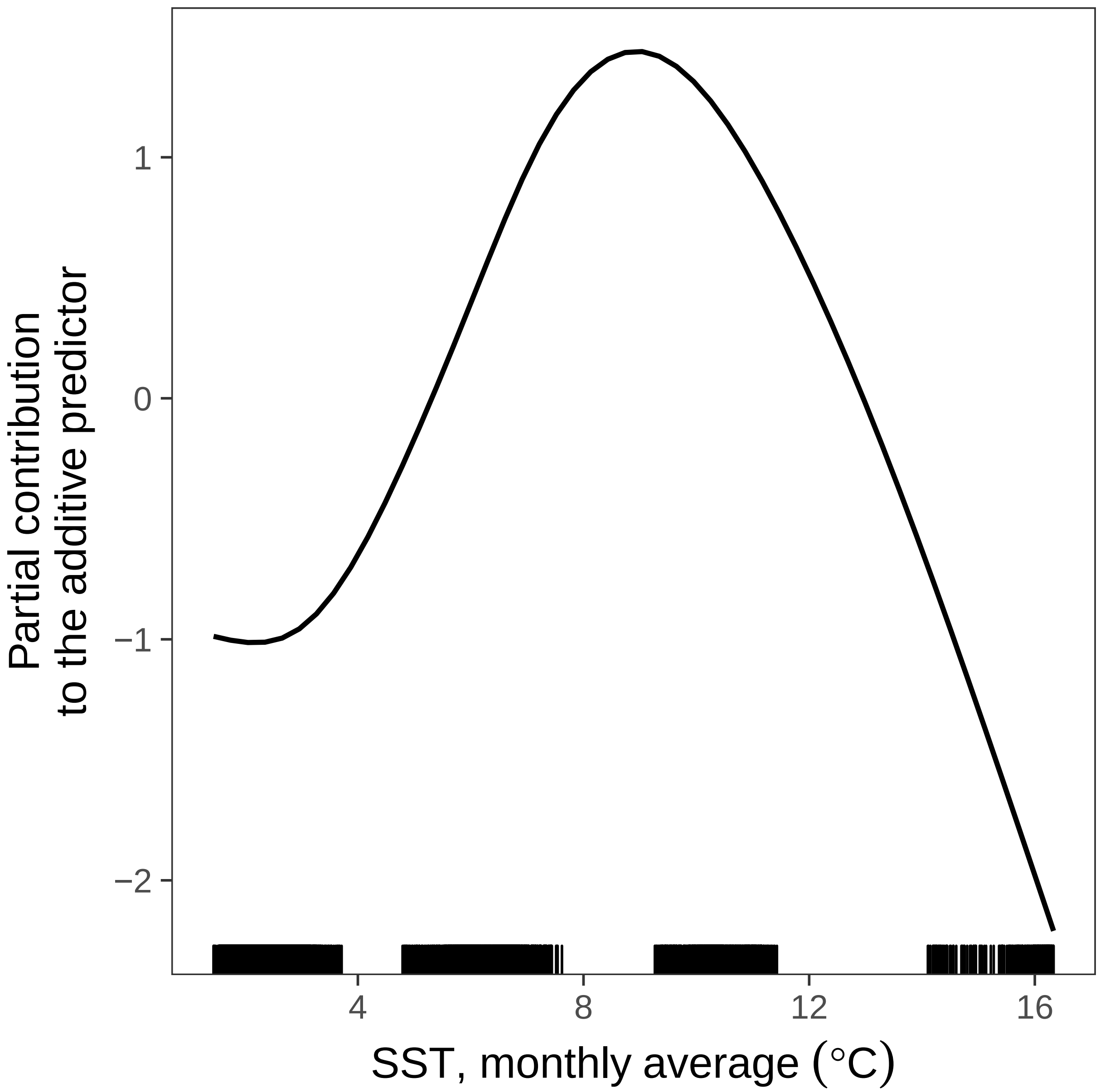
Partial contribution
to the additive predictor
1
0
−1
−2
4
8
12
16
SST, monthly average (°C)

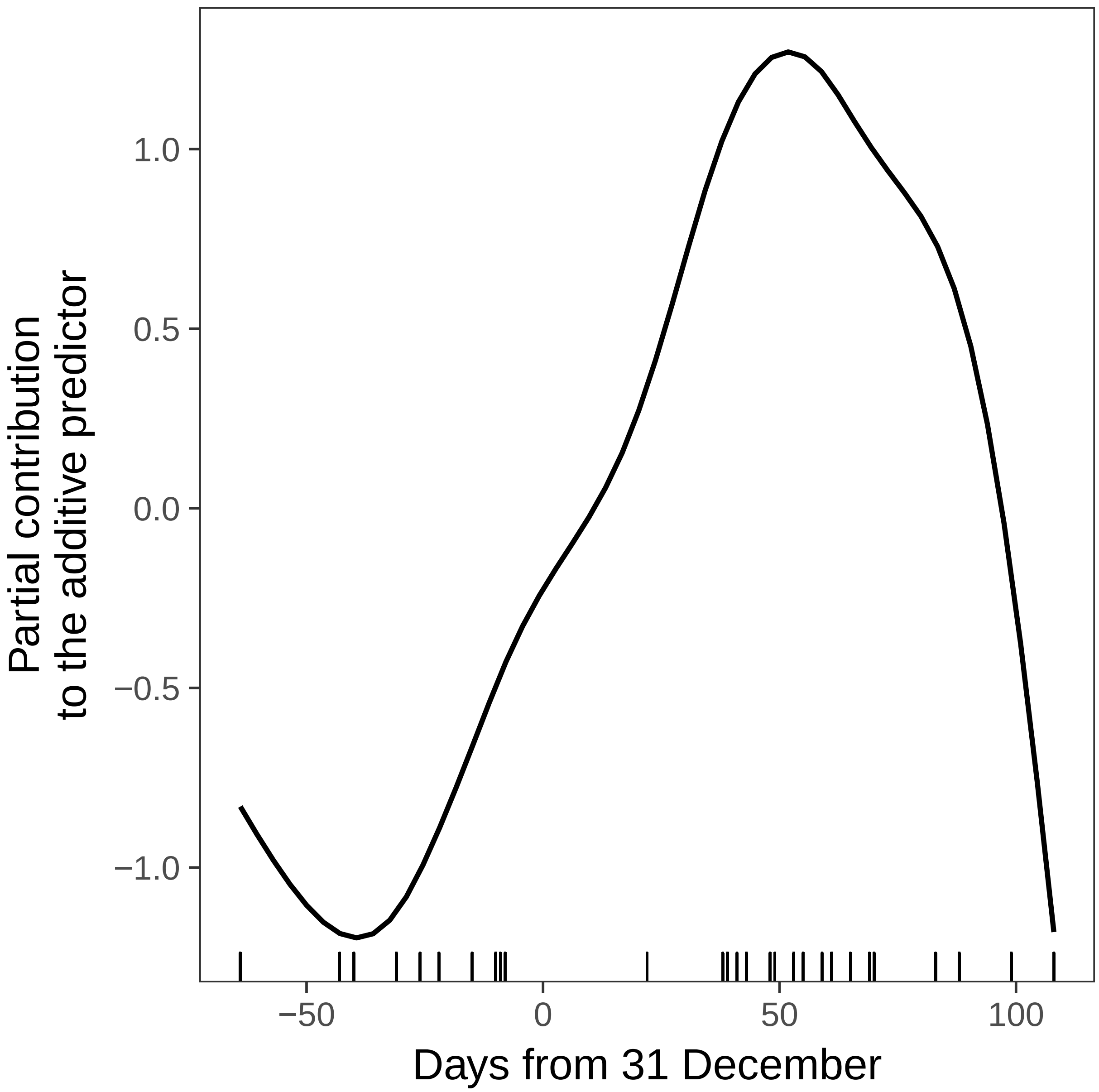

Partial contribution
to the additive predictor
1.0
0.5
0.0
−0.5
−1.0
−50
0
50
100
Days from 31 December

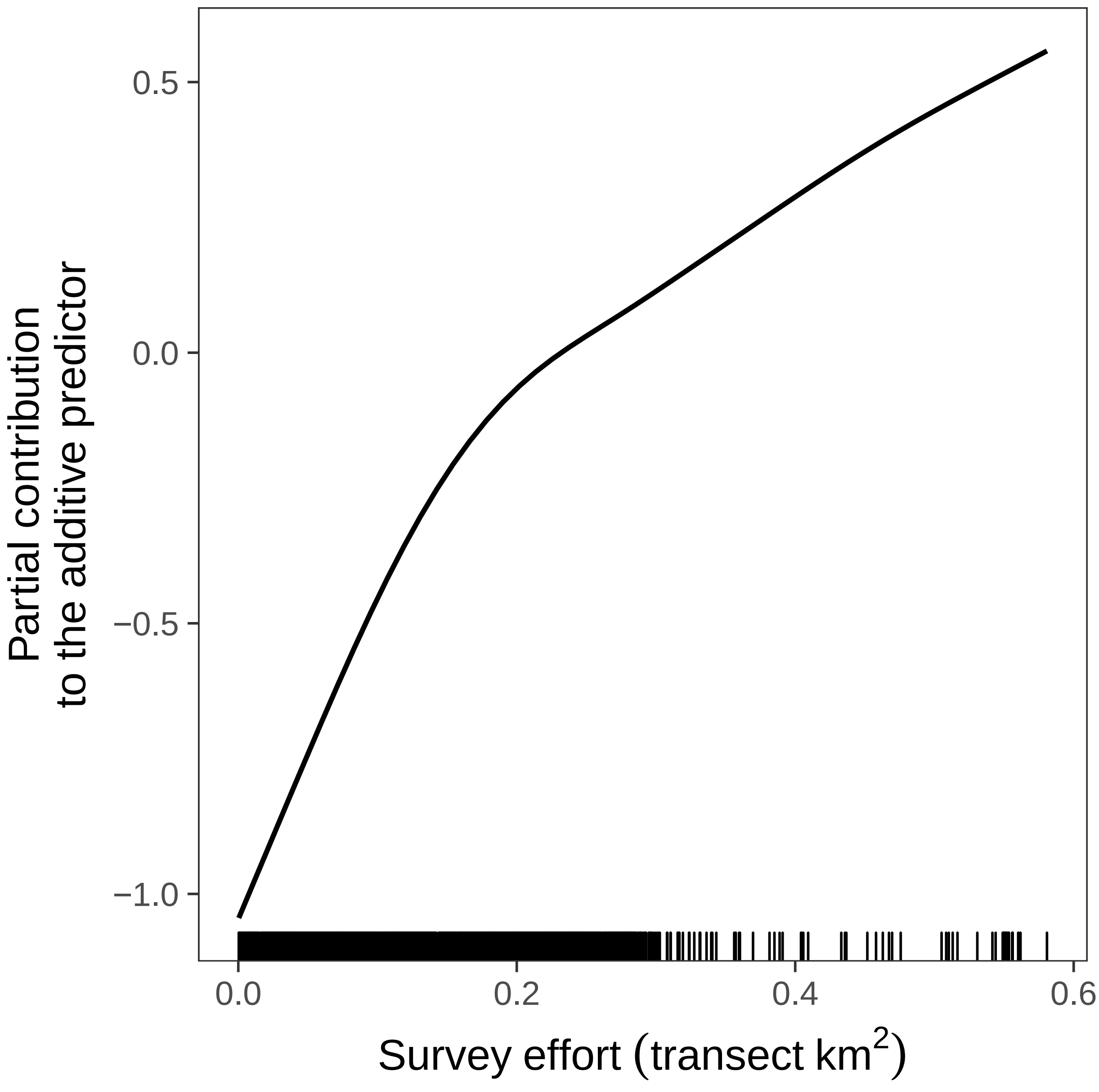
0.5
0.0
−0.5
−1.0
Partial contribution
to the additive predictor
0.0
0.2
0.4
0.6
Survey effort (transect km$^2$)

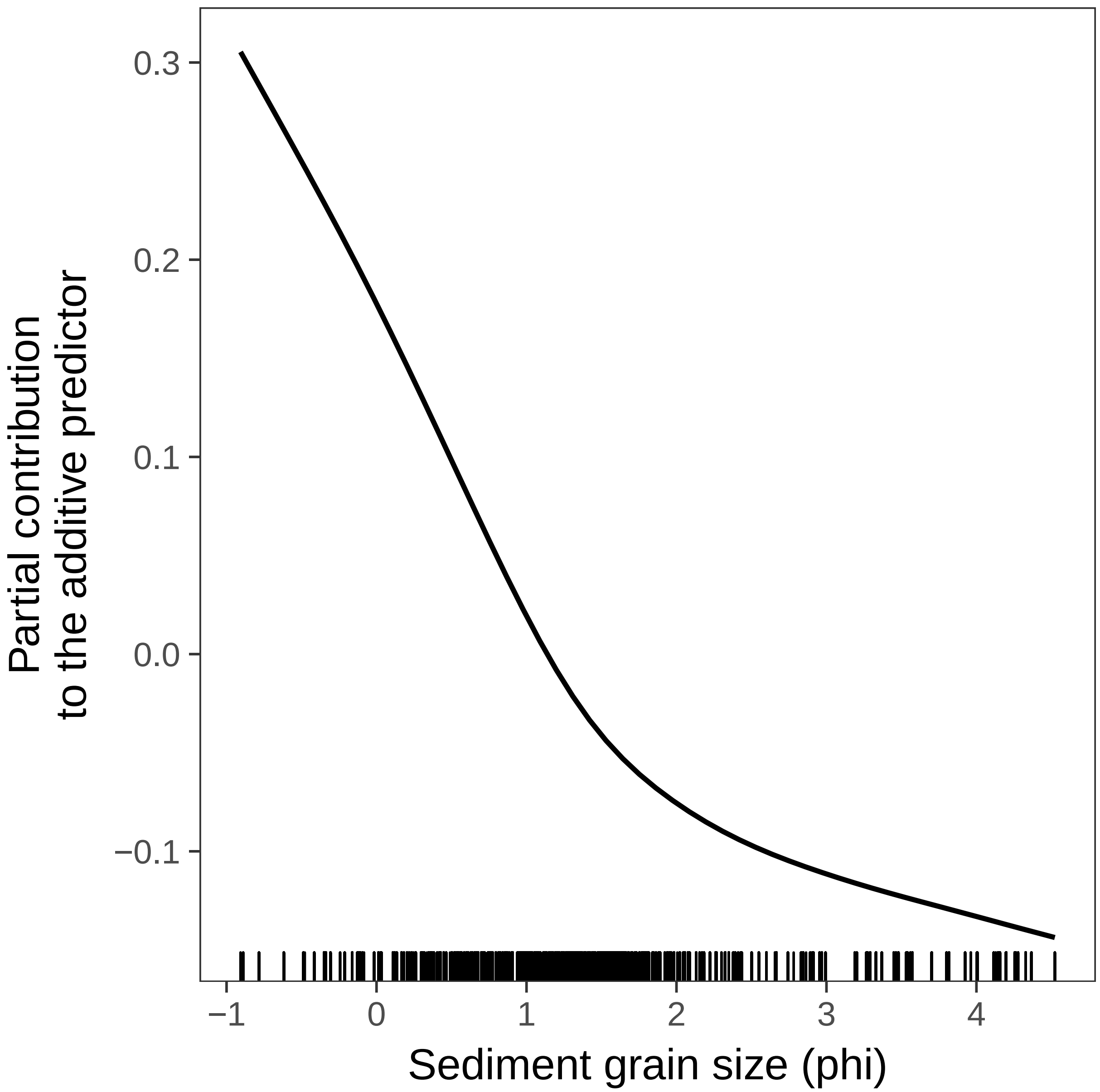
Partial contribution
to the additive predictor
0.3
0.2
0.1
0.0
−0.1
−1
0
1
2
3
4
Sediment grain size (phi)

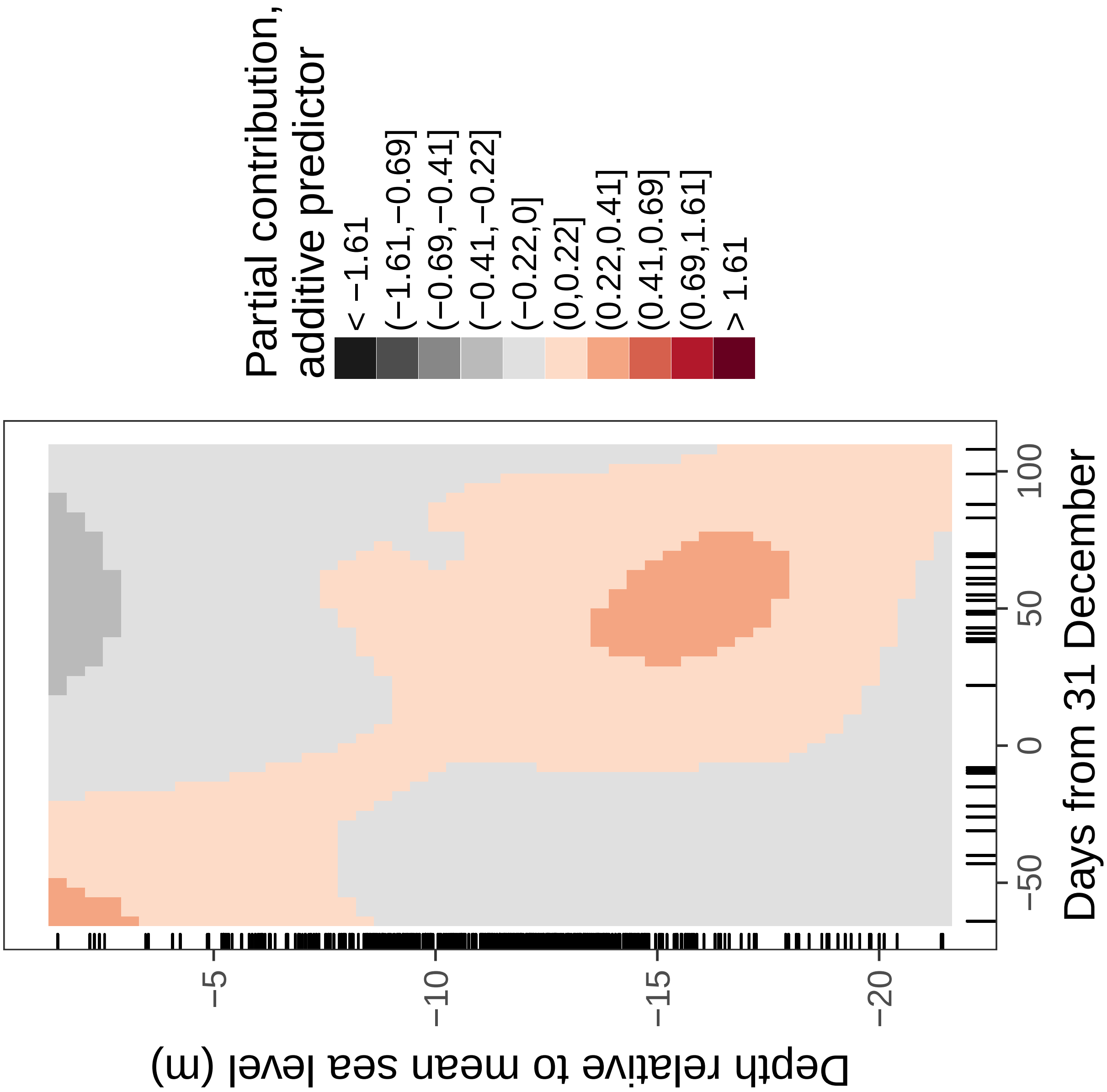

Partial contribution,
additive predictor
< −1.61
(−1.61,−0.69]
(−0.69,−0.41]
(−0.41,−0.22]
(−0.22,0]
(0,0.22]
(0.22,0.41]
(0.41,0.69]
(0.69,1.61]
> 1.61
Depth relative to mean sea level (m)
−5
−10
−15
−20
Days from 31 December
−50
0
50
100

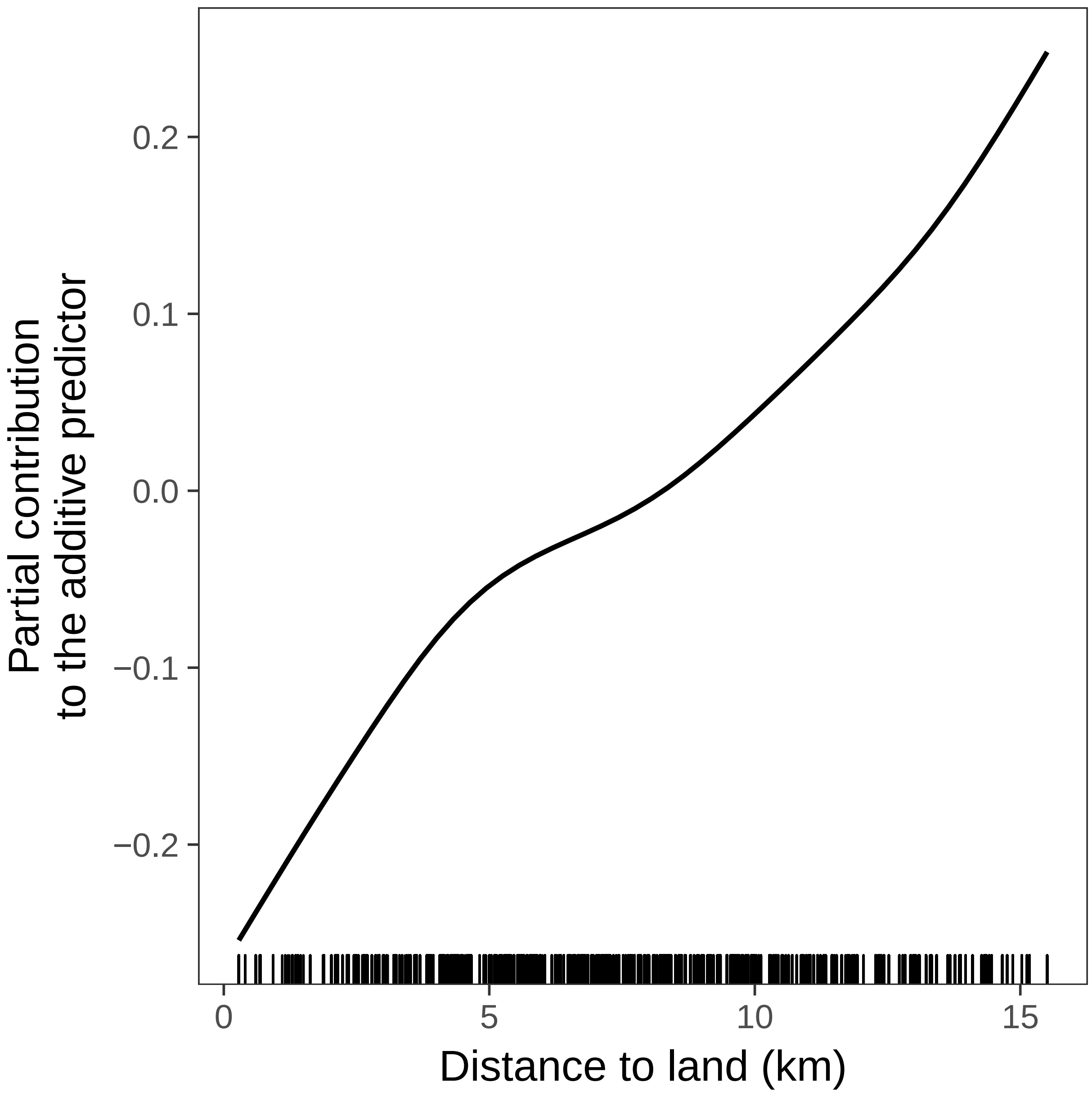
Partial contribution
to the additive predictor
0.2
0.1
0.0
−0.1
−0.2
0
5
10
15
Distance to land (km)

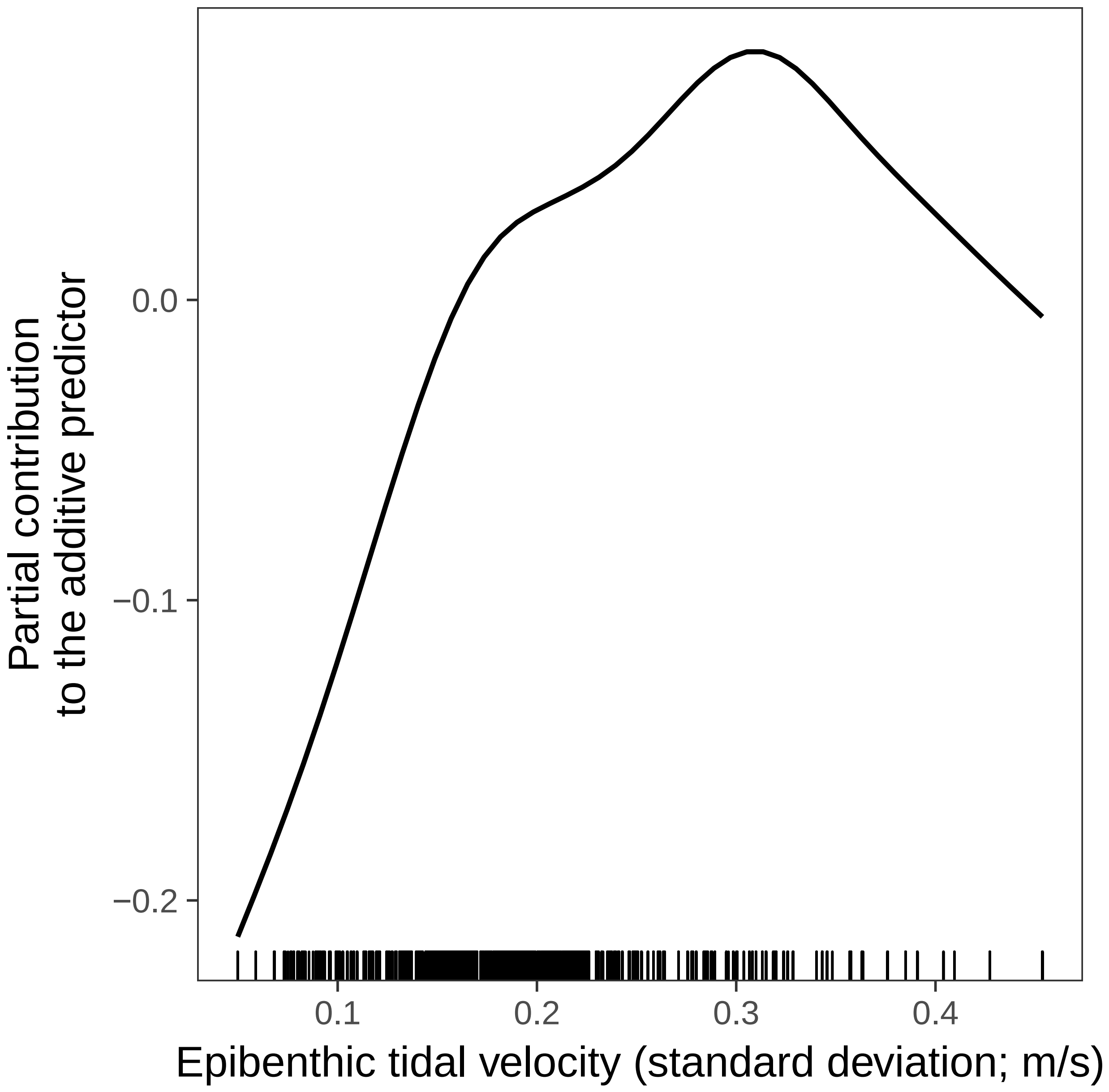
Partial contribution
to the additive predictor
0.0
−0.1
−0.2
0.1
0.2
0.3
0.4
Epibenthic tidal velocity (standard deviation; m/s)

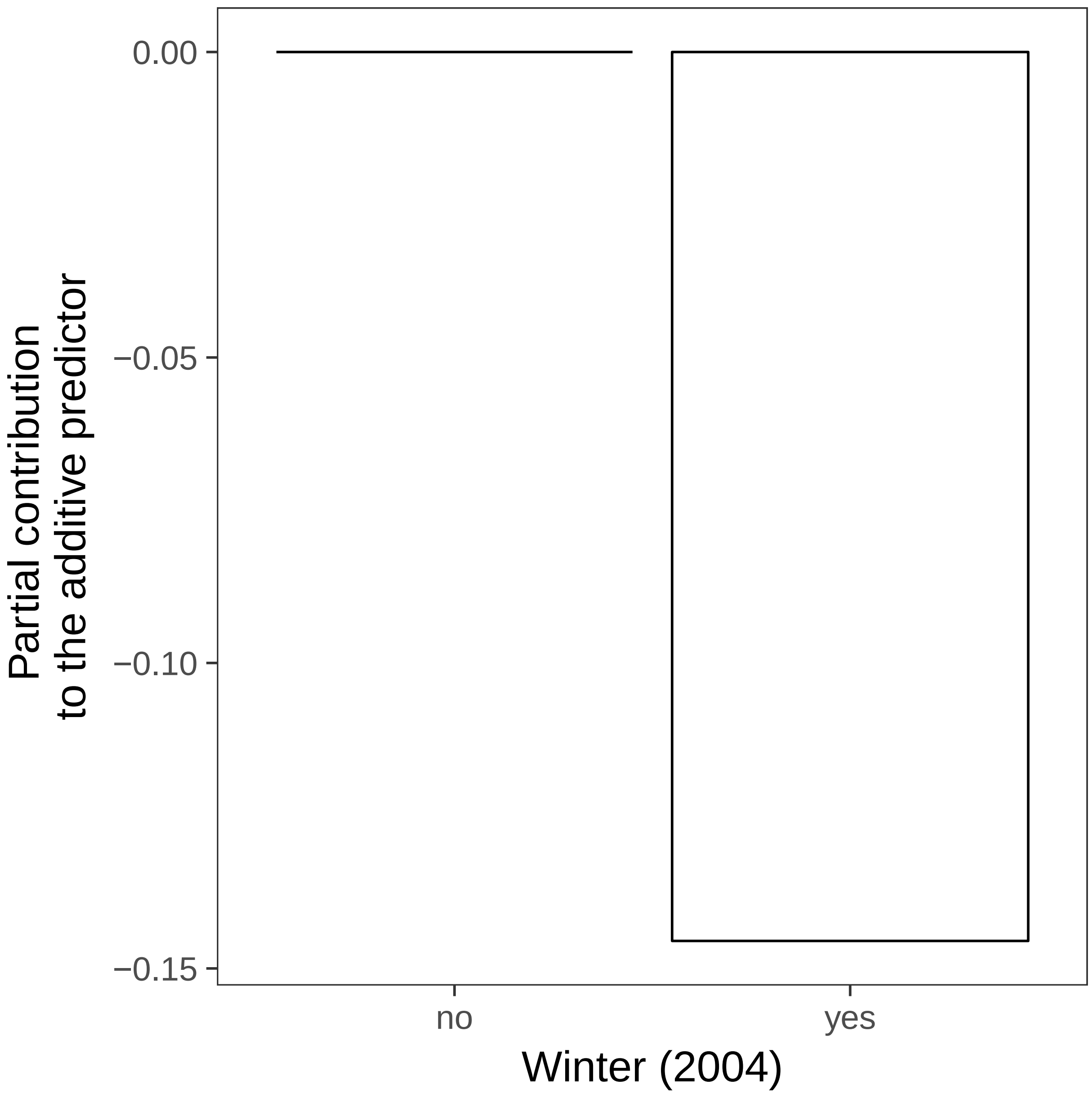
0.00
−0.05
−0.10
−0.15
Partial contribution
to the additive predictor
no
yes
Winter (2004)

**Conditional mean**

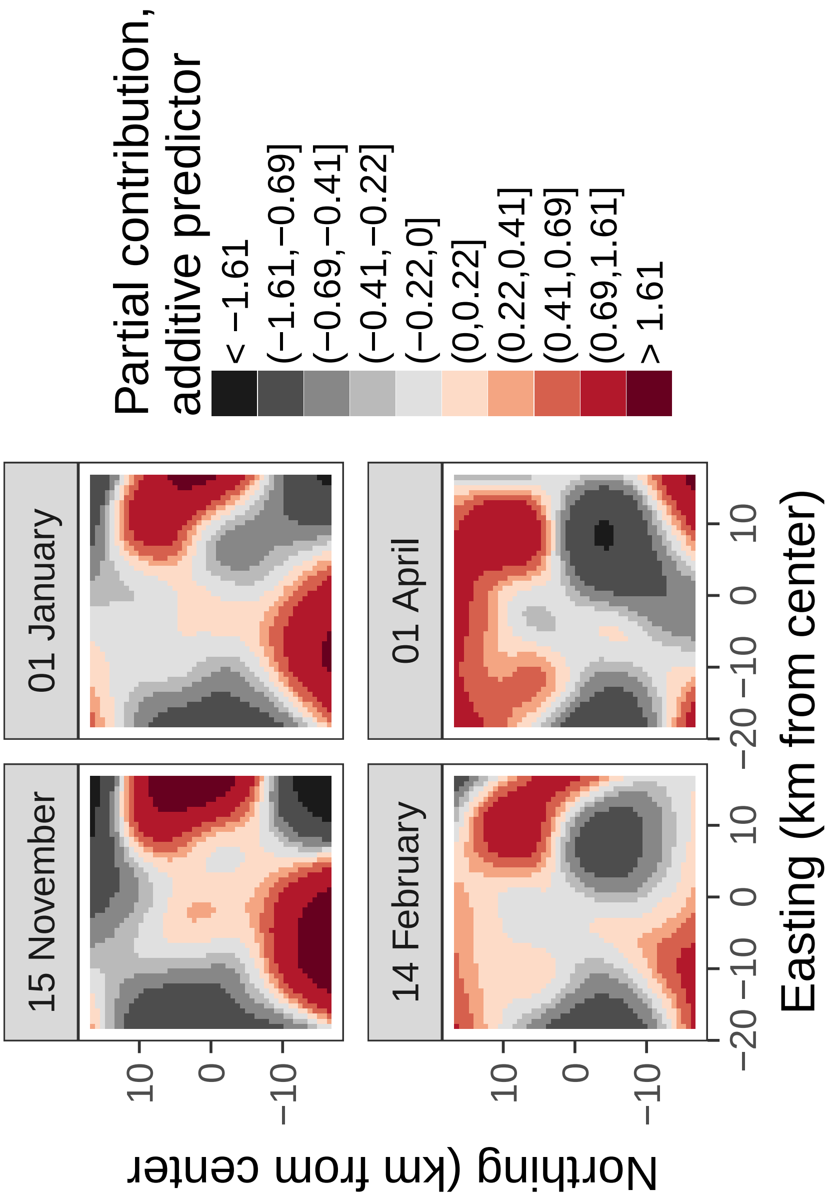

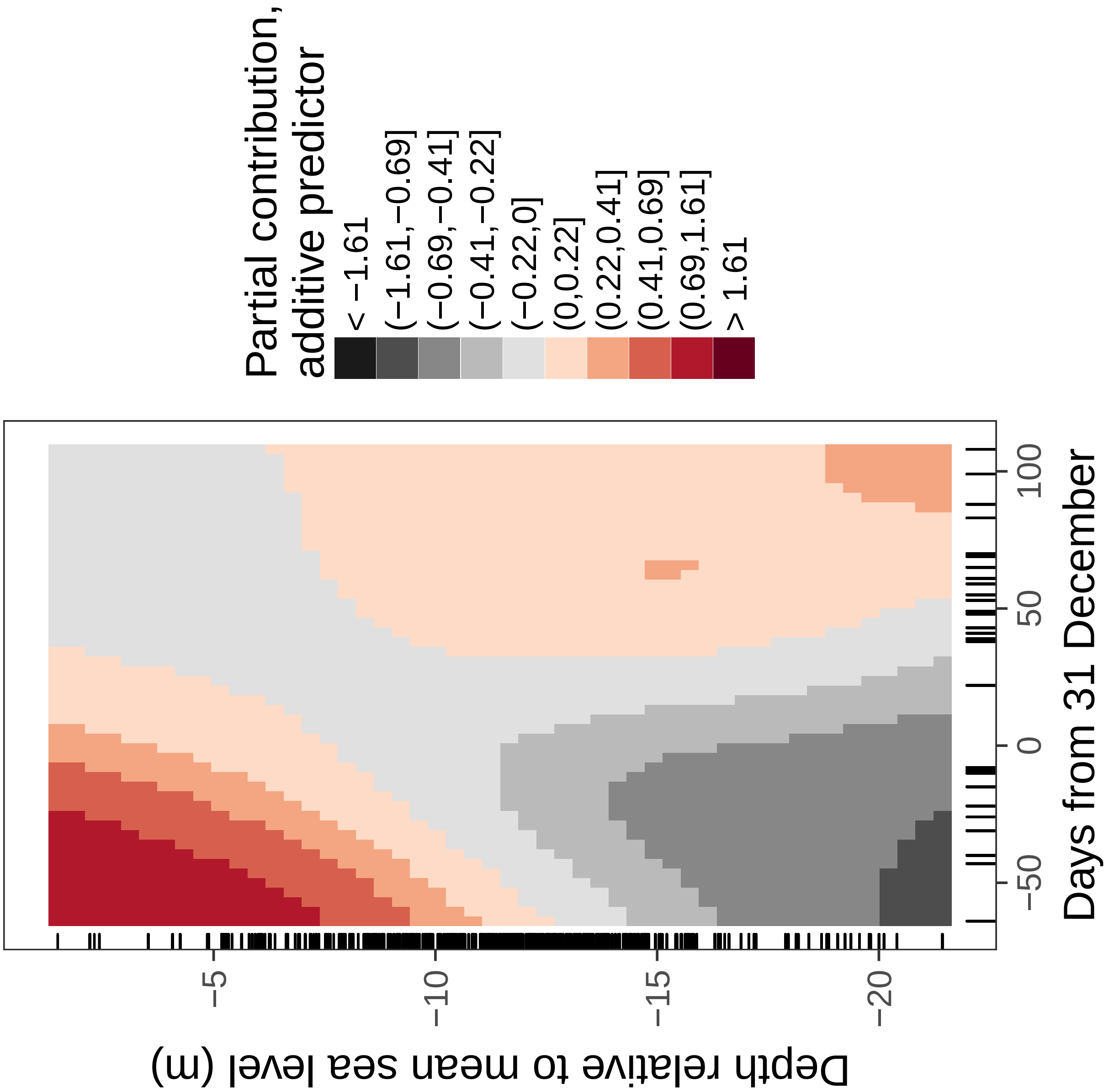

Partial contribution,
additive predictor
< −1.61
(−1.61,−0.69]
(−0.69,−0.41]
(−0.41,−0.22]
(−0.22,0]
(0,0.22]
(0.22,0.41]
(0.41,0.69]
(0.69,1.61]
> 1.61
Depth relative to mean sea level (m)
−5
−10
−15
−20
Days from 31 December
−50
0
50
100

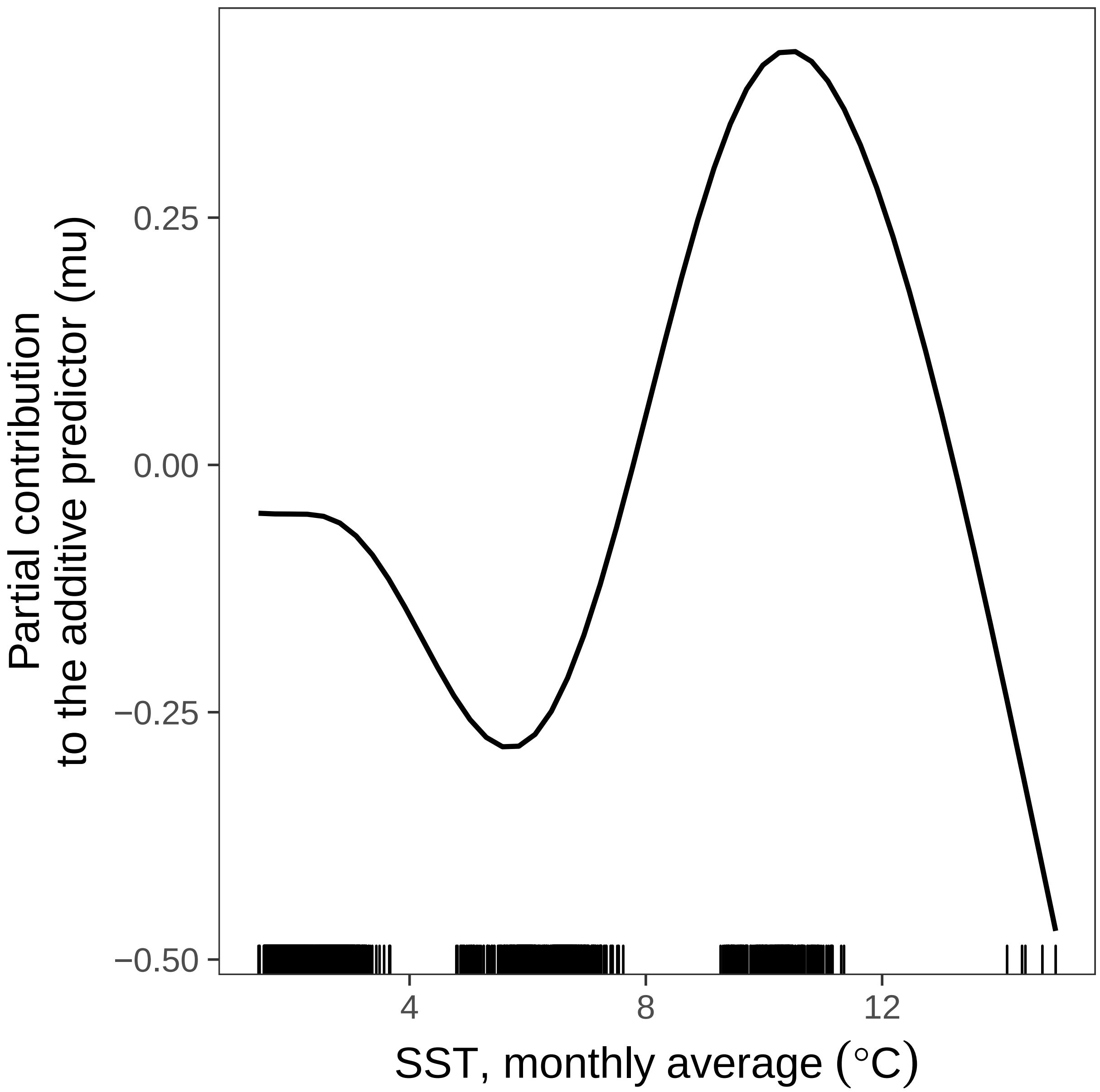
Partial contribution
to the additive predictor (mu)
0.25
0.00
−0.25
−0.50
4
8
12
SST, monthly average (°C)

**Conditional overdispersion**

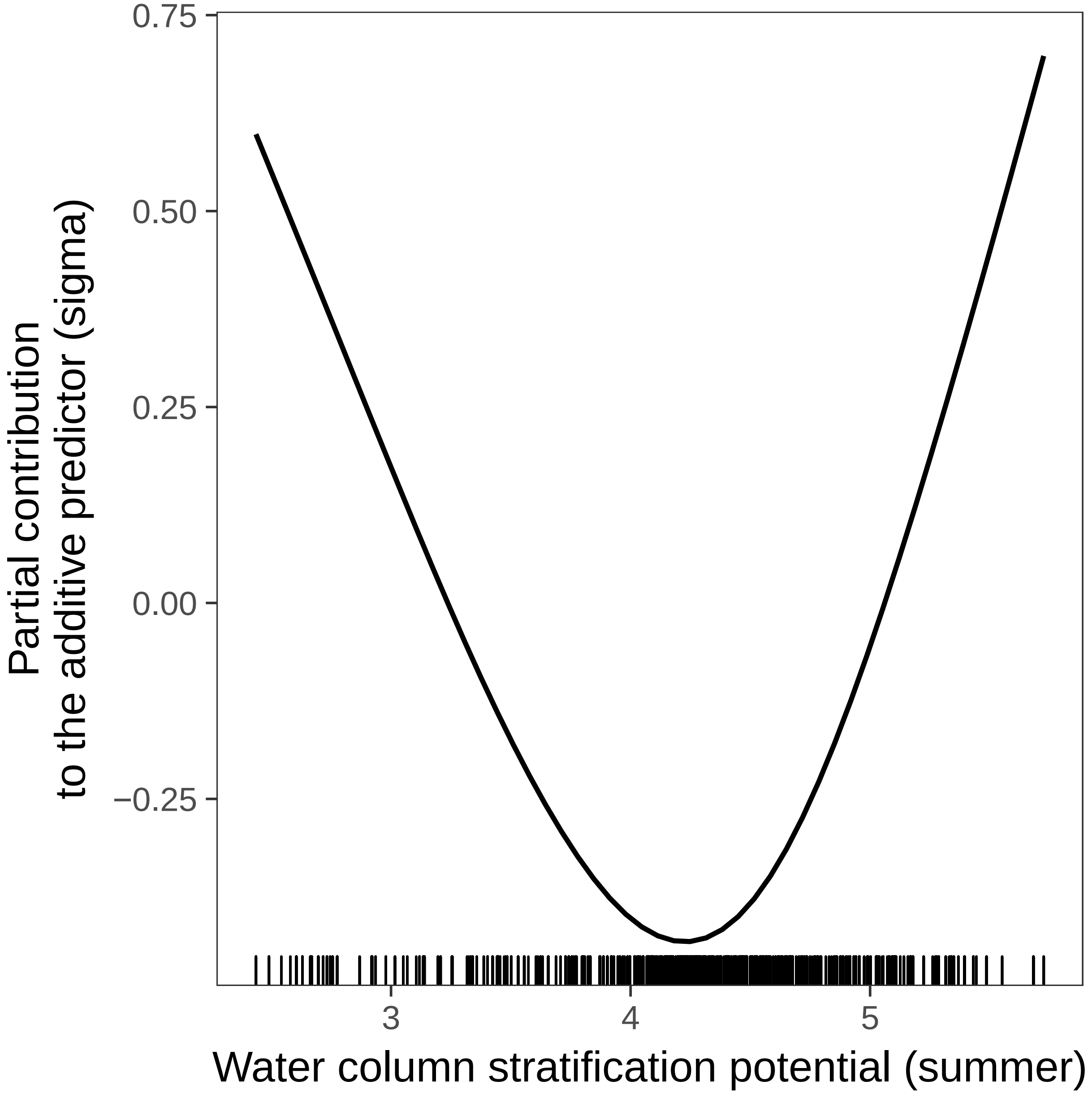

Supplement: Supplementary file 3 [file ECE3-9-2346-s003.pdf]
