# Supplementary material for: Modeling spatiotemporal abundance of mobile wildlife in highly variable environments using boosted GAMLSS hurdle models
Source: Ecol Evol. 2019 Feb 14;9(5):2346–64. doi: 10.1002/ece3.4738 (PMC6405508; doi:10.1002/ece3.4738)
Supplement: Supplementary file 4 [file ECE3-9-2346-s004.pdf]

S.4 Conditional overdispersion in sea duck abundance

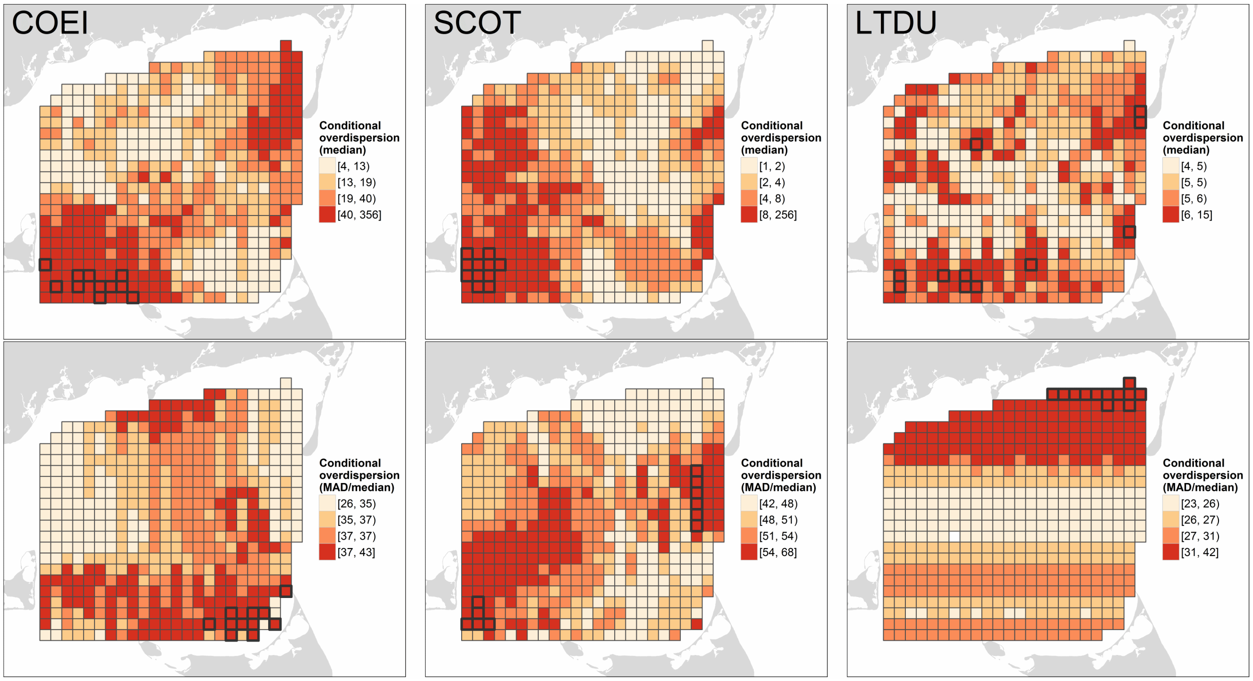

Figure S5 Overdispersion in the conditional abundance of Common Eider (COEI), scoter (SCOT), and Long-tailed Duck (LTDU) in Nantucket Sound during three winters, 2003 - 2005. Higher median conditional overdispersion values (top row) indicate increased variance in excess of the mean in the negative binomial model in counts of sea ducks, assuming their presence, in a 1.5 km x ca. 180 m transect in each segment predicted on 10 evenly-spaced dates from 15 November through 1 April in each winter. Spatiotemporal variation in conditional overdispersion (%; bottom row) is indicated by the median absolute deviation, MAD, relative to the median. Predicted values are categorized based on their quartiles; segments with highest overdispersion or variability(values $\geq$ 98th percentile) are outlined in black.
