# Supplementary material for: Modeling spatiotemporal abundance of mobile wildlife in highly variable environments using boosted GAMLSS hurdle models
Source: Ecol Evol. 2019 Feb 14;9(5):2346–64. doi: 10.1002/ece3.4738 (PMC6405508; doi:10.1002/ece3.4738)
Supplement: Supplementary file 5 [file ECE3-9-2346-s005.pdf]

S.5 Seasonal animation of predicted scoter occupancy and abundance

To provide an example of the seasonal dynamics that can characterize sea duck occupancy and abundance in Nantucket Sound, we animate predicted scoter occupancy (Figure S6) and overall abundance (Figure S7; scoter per 1.5 km x ca. 180 m) for every week between 1 November 2005 and 31 March 2006.

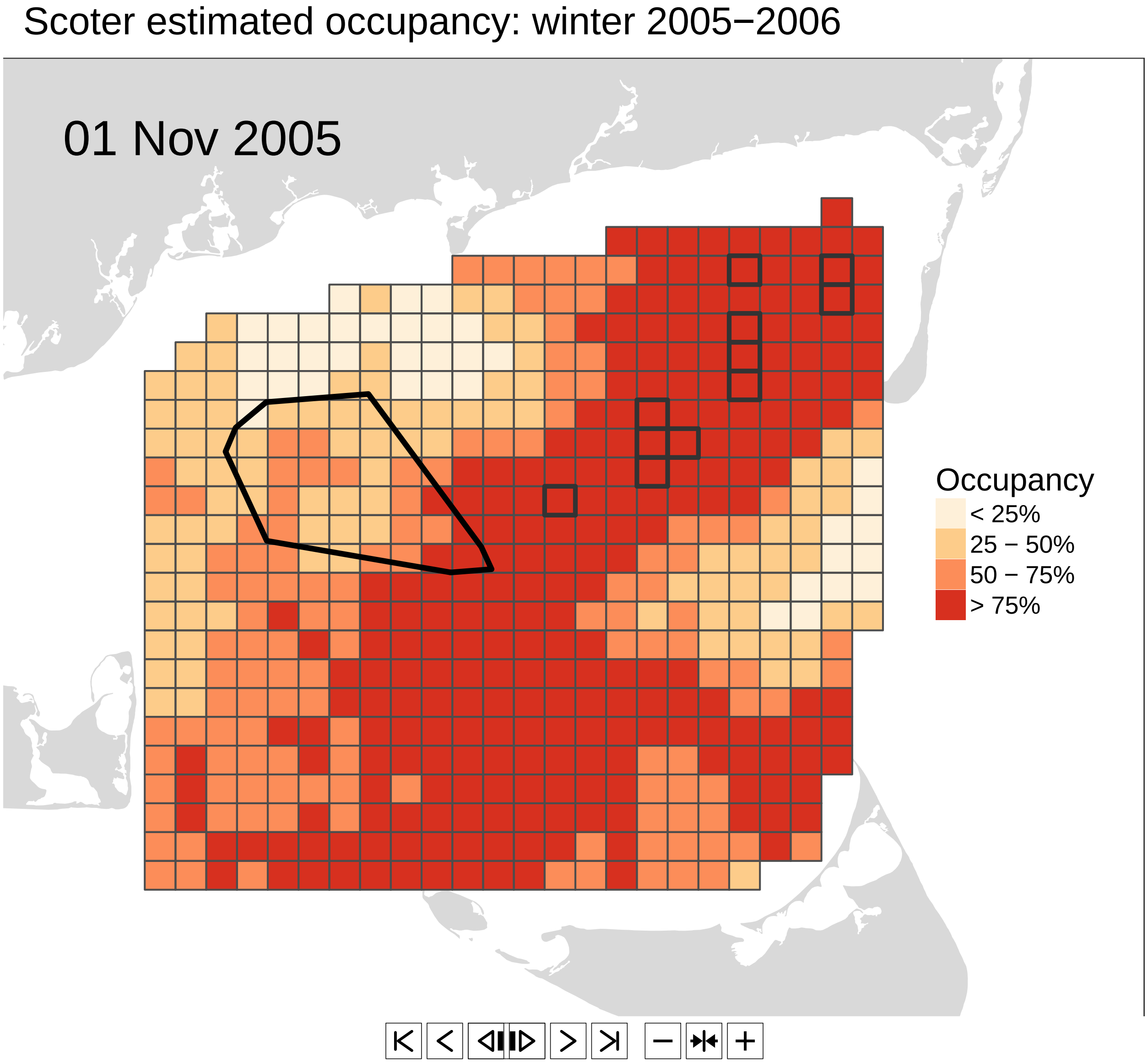

Figure S6

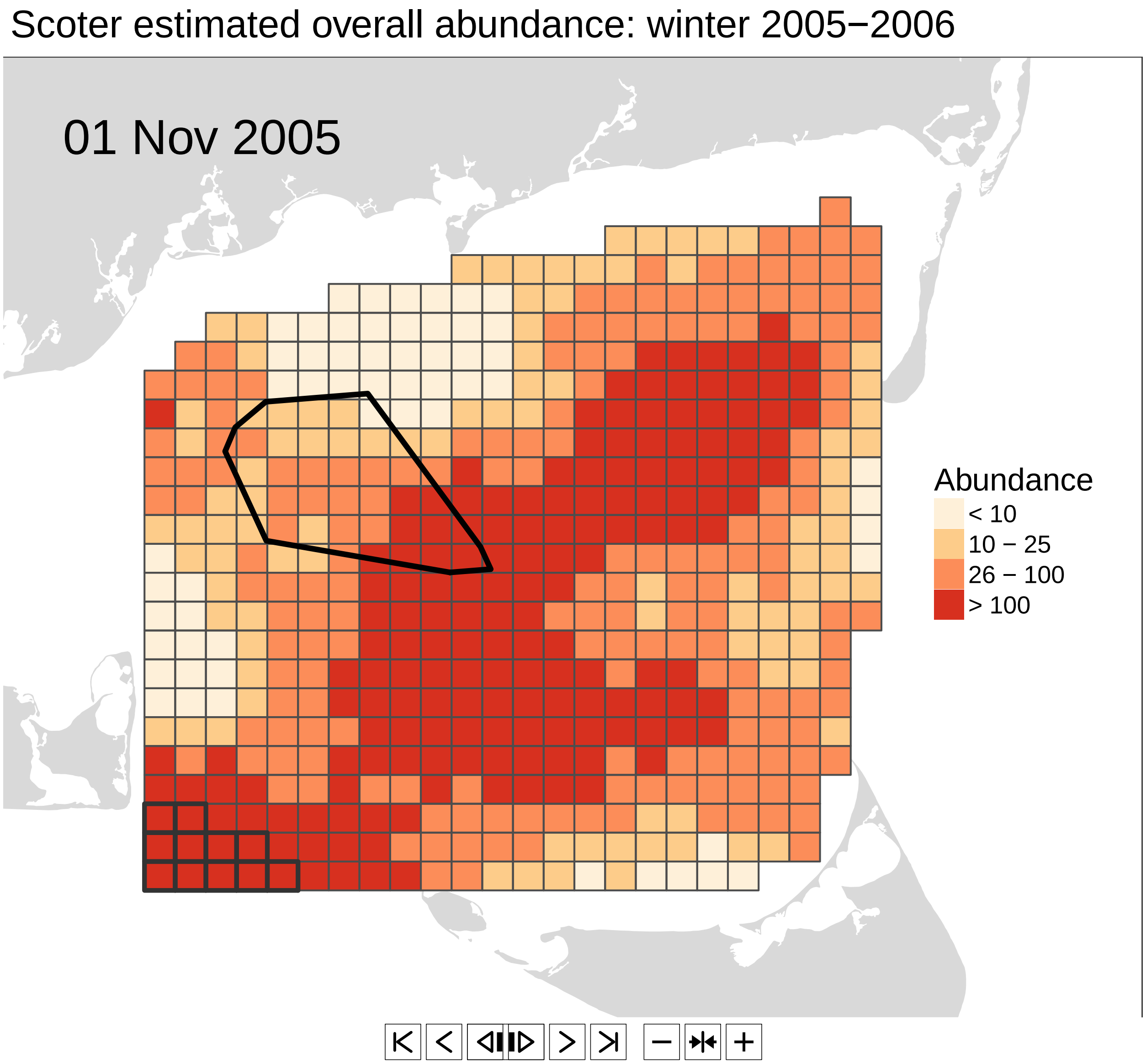
Scoter estimated overall abundance: winter 2005−2006
01 Nov 2005
Abundance
< 10
10 − 25
26 − 100
> 100

Figure S7
